# Behavioural nudges increase COVID-19 vaccinations

**DOI:** 10.1038/s41586-021-03843-2

**Published:** 2021-08-02

**Authors:** Hengchen Dai, Silvia Saccardo, Maria A. Han, Lily Roh, Naveen Raja, Sitaram Vangala, Hardikkumar Modi, Shital Pandya, Michael Sloyan, Daniel M. Croymans

**Affiliations:** 1 Anderson School of Management, University of California, Los Angeles, Los Angeles, CA USA; 2 Department of Social and Decision Sciences, Carnegie Mellon University, Pittsburgh, PA USA; 3 Department of Medicine, David Geffen School of Medicine, University of California, Los Angeles, Los Angeles, CA USA; 4 Office of Population Health and Accountable Care, University of California, Los Angeles, Los Angeles, CA USA; 5 Department of Medicine Statistics Core, David Geffen School of Medicine, University of California, Los Angeles, Los Angeles, CA USA; 6 Office of Health Informatics and Analytics, University of California, Los Angeles, Los Angeles, CA USA; 7 Department of Information Services and Solutions, UCLA Health System, Los Angeles, CA USA

**Keywords:** Decision making, Human behaviour

## Abstract

Enhancing vaccine uptake is a critical public health challenge^1^. Overcoming vaccine hesitancy^2,3^ and failure to follow through on vaccination intentions^3^ requires effective communication strategies^3,4^. Here we present two sequential randomized controlled trials to test the effect of behavioural interventions on the uptake of COVID-19 vaccines. We designed text-based reminders that make vaccination salient and easy, and delivered them to participants drawn from a healthcare system one day (first randomized controlled trial) (*n* = 93,354 participants; clinicaltrials number NCT04800965) and eight days (second randomized controlled trial) (*n* = 67,092 individuals; clinicaltrials number NCT04801524) after they received a notification of vaccine eligibility. The first reminder boosted appointment and vaccination rates within the healthcare system by 6.07 (84%) and 3.57 (26%) percentage points, respectively; the second reminder increased those outcomes by 1.65 and 1.06 percentage points, respectively. The first reminder had a greater effect when it was designed to make participants feel ownership of the vaccine dose. However, we found no evidence that combining the first reminder with a video-based information intervention designed to address vaccine hesitancy heightened its effect. We performed online studies (*n* = 3,181 participants) to examine vaccination intentions, which revealed patterns that diverged from those of the first randomized controlled trial; this underscores the importance of pilot-testing interventions in the field. Our findings inform the design of behavioural nudges for promoting health decisions^5^, and highlight the value of making vaccination easy and inducing feelings of ownership over vaccines.

## Abstract (web summary)

Two randomized controlled trials demonstrate the ability of text-based behavioural ‘nudges’ to improve the uptake of COVID-19 vaccines, especially when designed to make participants feel ownership over their vaccine dose.

## Main

Vaccines have been crucial for eradicating or controlling several deadly infectious diseases^1^. However, mobilizing people to get vaccines remains a challenge. Low or delayed vaccination uptake continues to threaten global health, and can lead to outbreaks of vaccine-preventable diseases^6^. Developing evidence-based communication strategies to enhance voluntary vaccine uptake is therefore critical^4^. Previous work suggests two major approaches to increasing vaccinations^3^. The first aims to boost vaccine uptake intentions among those who are uncertain about vaccination. Given that changing intentions is insufficient^7^, the second approach involves helping people to follow through on their vaccination intentions and overcome sources of friction, such as forgetfulness^8^, hassle costs^9^ and procrastination^10,11^.

These approaches could help to increase vaccination rates in the context of the current COVID-19 pandemic^12^, which has had unprecedented costs^13^. Despite the growing availability of COVID-19 vaccines, 30% of US adults were still either unwilling or uncertain about getting the COVID-19 vaccine in late June 2021, and the hesitancy rate was similarly high in several other countries that had vaccines available^14^. Barriers to action may further lower vaccination rates among those who intend to get inoculated.

Nudges, defined as interventions that alter ‘people’s behavior in a predictable way without forbidding any options or significantly changing economic incentives’^15^, could improve the uptake of COVID-19 vaccines^16^. Low-cost behavioural interventions such as these have been effectively applied to other health-related decisions^5^, such as healthy eating^17^, exercising^18^ and influenza vaccinations^19–21^. To maximize vaccine uptake, it is critical to understand how to best design behavioural interventions to boost intentions to get vaccinated, remove barriers to following through on good intentions or both^3^.

Here we report data from two sequential large-scale randomized controlled trials (RCTs) that investigate whether nudging people to get vaccinated, using reminders that are carefully designed to reduce barriers to following through, can improve the uptake of COVID-19 vaccines. Reminders are a popular nudge^22^ and have proven effective across policy-relevant domains^8,20,23,24^. We further examine the benefits of combining our reminders with additional interventions, including (1) behaviourally informed messaging designed to amplify individuals’ desire to get vaccinated and (2) a traditional information-provision intervention aimed at correcting the misconceptions that drive vaccine hesitancy^25,26^. Testing the effects of interventions on actual uptake of COVID-19 vaccines extends previous work that has studied hypothetical interventions^27,28^.

## Promoting vaccine uptake

**Fig. 1:**
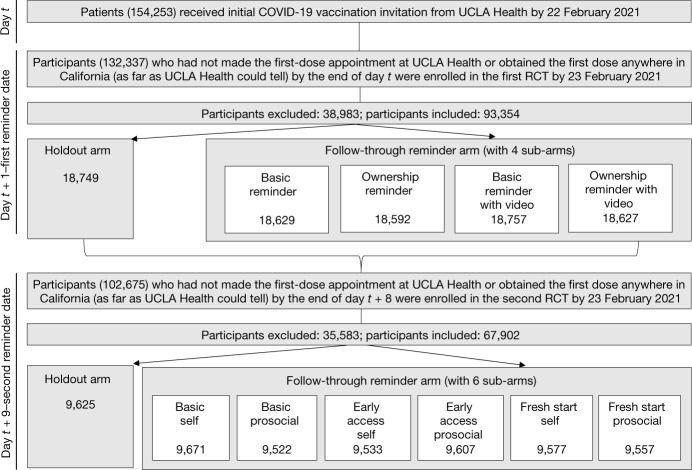
Timeline, assessment for eligibility and randomization of two sequential RCTs. Timeline, eligibility for enrolment, the total number of participants excluded from the analysis, the total number of participants included in the analysis, and the number of participants who were randomized into each condition and included in the analysis are displayed here for the first and second RCTs. *t* is the date on which participants received the initial invitation to take up a COVID-19 vaccine from UCLA Health. The first reminder date fell on the first weekday after the initial invitation was sent, and the second reminder date fell on the first weekday after the eighth day following the initial invitation. Exceptions were that participants who received the initial invitation during 19–29 January 2021 were enrolled in the first RCT on 1 February 2021 and the second RCT on 9 February 2021, owing to the delay in setting up the infrastructure needed to run the RCTs. In the first RCT, 38,983 participants were sequentially excluded from the analysis, including (1) 33,533 individuals who obtained the first dose before the first reminder date according to the vaccination records UCLA Health could access on 25 May 2021; (2) 5,392 individuals who made the first-dose appointment at UCLA Health before 15:00 h on the first reminder date; and (3) 58 individuals who were under 18 years old. In the second RCT, 35,583 participants were sequentially excluded from the analysis, including (1) 35,127 individuals who obtained the first dose before the second reminder date according to the vaccination records UCLA Health could access on 25 May 2021; (2) 408 individuals who made the first-dose appointment at UCLA Health before 15:00 h on the second reminder date; and (3) 48 individuals who were under 18 years old.

We conducted two preregistered RCTs at University of California, Los Angeles (UCLA) Health (‘Data availability’ in Methods). Participants in these RCTs were drawn from the UCLA Health primary and speciality care attributed patient list. Starting from 29 January 2021, once patients became eligible for the COVID-19 vaccine, UCLA Health sent them an initial invitation to schedule their vaccination appointment. On the first weekday after the initial invitation (hereafter, the ‘first reminder date’), we enrolled eligible patients (hereafter, ‘participants’﻿) in the first RCT. On the first weekday after the eighth day following the initial invitation (hereafter, the ‘second reminder date’), we enrolled participants eligible for the second RCT into it. The timeline and eligibility criteria are provided in ‘Enrolment and eligibility for RCTs’ in Methods; Fig. 1 shows the timeline, eligibility and randomization of the two RCTs.

In both RCTs, we randomized whether participants received text-message-based reminders or not. All reminders shared two elements that were intended to address two barriers to action. First, all reminders made vaccination top of mind to curb forgetfulness and prompt people to adopt the target behaviour^8, 22^. Second, all reminders sought to reduce inconvenience as a potential source of friction^22^ by including a link to the appointment-scheduling website and allowing participants to easily book their appointment immediately.

Our primary outcome was whether participants scheduled their first-dose appointment at UCLA Health within six days of receiving a text reminder. Our secondary outcome was whether participants obtained the first dose at UCLA Health within four weeks of the reminder; the reasoning behind these time windows is given in ‘Outcome measures for RCTs’ in Methods.

We focus our data reporting on participants who were enrolled in the RCTs by 23 February 2021, as specified in our preregistration. All exclusion criteria and analyses were preregistered (‘Enrolment and eligibility for RCTs’ in Methods, Supplementary Information sections 1.1 and 1.3).

### First-reminder RCT

On the first reminder date, we randomly assigned participants enrolled in the first RCT at a 4:1 ratio to the ‘follow-through reminder’ arm, in which they received a text reminder at 15:00 h that encouraged them to schedule a vaccination appointment, or to the ‘holdout’ arm, in which they did not get a reminder.

We nested a 2 × 2 factorial design within the follow-through reminder arm to test whether reminders become more effective when combined with another behaviourally informed intervention to motivate action and/or with an information intervention that aims at shifting vaccination intentions.

The first factor varied whether the reminder attempted to further amplify people’s desire to act by inducing feelings of psychological ownership over the vaccine^29,30^. Reminders containing the ownership intervention (designated ‘ownership reminder’ and ‘ownership reminder with video’) indicated the vaccine had ‘just been made available for you’ and encouraged participants to ‘claim your dose’. We used online experiments to confirm that such language would make participants feel more strongly that the vaccine was already theirs (ordinary least squares (OLS) regressions, *B* = 0.376, s.e. = 0.084, *P* < 0.001, *n* = 1,987; *B* = 0.389, s.e. = 0.116, *P* < 0.001, *n* = 1,168) (Supplementary Tables 26, 36). Previous research has shown that similar language—such as ‘the flu vaccine is reserved for you’—increased uptake of influenza vaccinations^20^; psychological ownership could be one of the mechanisms at play.

The second factor manipulated whether the reminder contained a link to a 2-min video that provided information on COVID-19 and vaccine effectiveness, with the goal of correcting common misconceptions and boosting vaccination intentions. The video intervention was used in the ‘basic reminder with video’ and ‘ownership reminder with video’ sub-arms. We based the video on a literature review of vaccine hesitancy^3,31,32^ and our January 2021 survey of residents of California (USA) (*n* = 515) (‘Vaccination intention survey’ in Methods), which allowed us to identify common misconceptions about COVID-19 and authorized vaccines. A similar video intervention was used in previous work to increase influenza vaccinations^20^.

Our analysis includes 93,354 participants (43.3% male, 53.5% white (excluding Hispanic or Latino) (all racial demographic data use self-reported terms), average age = 72.8, s.d. = 10.3). Study arms were well-balanced on demographic characteristics (Extended Data Table 1). All reported effect sizes come from OLS regressions (or, precisely, a linear probability model^33^, given our binary outcome measures) with heteroscedasticity-robust standard errors that control for participant gender, age, race, ethnicity, preferred language, social vulnerability index, COVID-19 risk score and fixed effects of initial invitation dates. The results are robust to removing control variables, using logistic regressions and conducting intent-to-treat analyses with all participants enrolled in our RCTs by 23 February 2021 (Supplementary Information section 1.5).

**Fig. 2:**
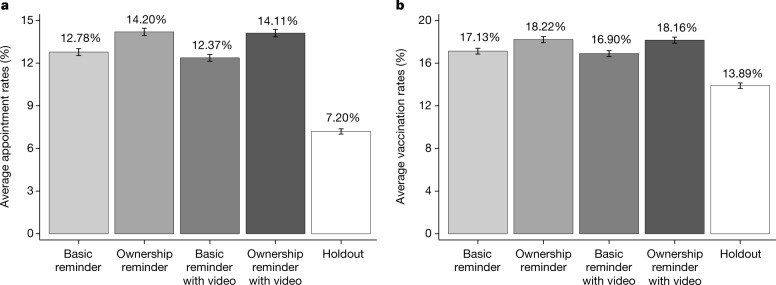
Appointment and vaccination rates at UCLA Health by condition for the first RCT. **a**, **b**, Proportion of participants in each condition who scheduled an appointment for the first dose of the COVID-19 vaccine at UCLA Health between 15:00 h on the first reminder date and 23:59 h on the fifth day following the first reminder date (**a**) and the proportion of participants in each condition who obtained the first dose of the COVID-19 vaccine at UCLA Health within four weeks of the first reminder date (**b**). Error bars represent ± 1 s.e.m. The number of participants in each condition (from left to right in each panel) is 18,629, 18,592, 18,757, 18,627 and 18,749.

In the holdout arm, 7.20% of participants made the first-dose appointment within six days of the first reminder date, and 13.89% received the first dose at UCLA Health within four weeks (Fig. 2). Our OLS regressions estimate that receiving a text reminder boosted appointment rates within six days by 6.07 percentage points and vaccination rates within four weeks by 3.57 percentage points (Extended Data Table 2), amounting to a relative increase of 84.33% and 25.71%, respectively. All reminder types outperformed the holdout arm (Extended Data Table 2). The top-performing reminder type contained the ownership language, and boosted appointment and vaccination rates at UCLA Health by 6.83 (94.84%) and 4.12 (29.63%) percentage points, respectively, relative to the holdout arm.

**Fig. 3:**
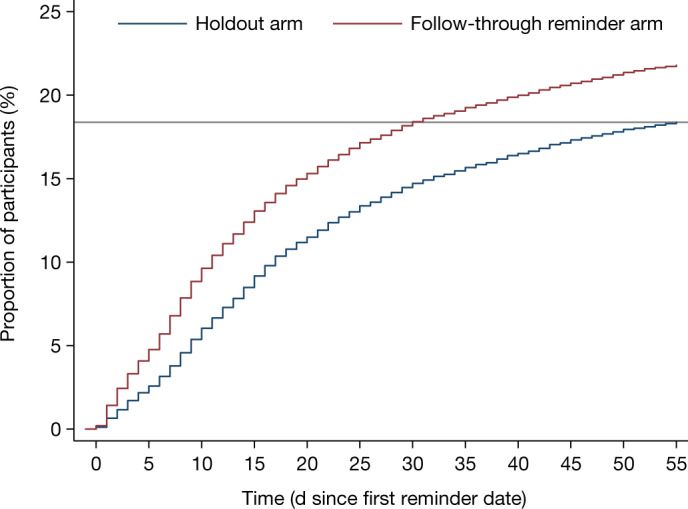
Kaplan–Meier curves reflecting the proportion of participants who had obtained the first dose at UCLA Health by a given day after the first reminder date in the first RCT. Kaplan–Meier curves tracking the percentage of participants in the holdout arm (blue) (*n* = 18,749) versus the follow-through reminder arm (red) *(n* = 74,605) of the first RCT who had obtained the first dose of COVID-19 vaccine at UCLA Health by a given day from the first reminder date (0 on the *x* axis) onward. All participants were right-censored at 55 days after the first reminder date. The solid horizontal line indicates that 18.38% of participants in the holdout arm had obtained the first dose at UCLA Health by the end of 55 days after the first reminder date.

The gap between the follow-through reminder and holdout arms in vaccinations at UCLA Health persisted for eight weeks (Fig. 3), which suggests that reminders increased the number of vaccinated participants for as long as we observed (rather than only accelerating vaccinations). Notably, even if the holdout arm eventually caught up after the two months we observed, accelerating vaccination still benefits society^34^.

Within the follow-through reminder arm, adding the ownership language to the reminder further increased appointment and vaccination rates at UCLA Health by 1.51 and 1.09 percentage points, respectively (Extended Data Table 2), compared to the 12.58% appointment rates and 17.01% vaccination rates among people who received a reminder without such language. By contrast, we found no evidence that inviting participants to watch the video improved either outcome variable, relative to reminders without a video (Extended Data Table 2).

**Fig. 4:**
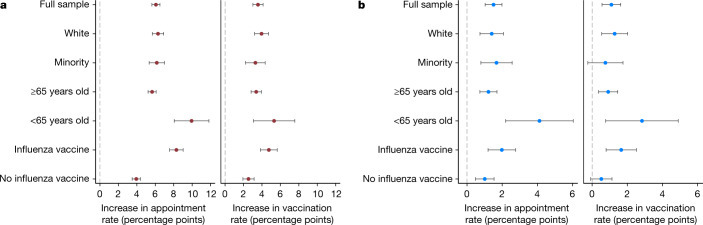
Regression-estimated increase in appointments and vaccinations induced by reminders. **a**, **b**, Regression-estimated increase in appointment rates at UCLA Health within six days of the first reminder date (left panel in **a**, **b**) and vaccination rates at UCLA Health within four weeks of the first reminder date (right panel in **a**, **b**), induced by receiving a reminder (versus holdout) (**a**) and by receiving a reminder with ownership language (versus one without) (**b**) across participant subgroups in the first RCT. The full sample referred to 93,354 participants included in the analysis of the first RCT. ‘White’, subsample including 49,909 participants who identified as white (excluding Hispanic or Latino individuals); ‘minority’, subsample includes 29,784 participants who identified as Asian, Black, American Indian or Alaska Native, Native Hawaiian or Pacific Islander, other race (excluding participants whose race was unknown), and/or Hispanic or Latino. The ‘≥65 years old’ subgroup includes 84,075 participants who were at least 65 years old; the ‘<65 years old’ subgroup includes 9,279 participants under 65 years old. The ‘influenza vaccine’ subgroup includes 46,757 participants who received the influenza vaccine in either of two recent influenza seasons; the ‘no influenza vaccine’ subgroup includes 46,597 participants who did not receive an influenza vaccine in two recent influenza seasons. Extended Data Table 2, Supplementary Tables 3, 5, 6, 10, 11, 13 provide complete OLS regression results graphed here and the corresponding sample sizes. Error bars represent 95% confidence intervals of estimated increases.

The average effect of a reminder held for both participants who received the influenza shot in either of the two recent seasons (*n* = 46,757) and those who did not (*n* = 46,597) (Fig. 4) but was larger among the former than the latter group, by 4.4 percentage points for appointments (OLS regression, *B* = 0.044, s.e. = 0.004, *P* < 0.001 for the interaction) and 2.3 percentage points for vaccinations at UCLA Health (OLS regression, *B* = 0.023, s.e. = 0.006, *P* < 0.001 for the interaction) (Supplementary Table 6).

Because our sample consists of predominantly elderly and white participants, we confirmed (Fig. 4) that the effects of follow-through reminders and ownership language largely held for racial and ethnic minorities as defined in Fig. 4 (*n* = 29,784) and participants under 65 years old (*n* = 9,279). Notably, the average effects of follow-through reminders on both appointments and vaccinations were comparable across white (*n* = 49,909), Hispanic (*n* = 10,624), Black (*n* = 5,109) and Asian (*n* = 7,553) participants (Extended Data Table 2). Identifying solutions to improving vaccine uptake among racial and ethnic minority groups is critical, as these groups have been disproportionately hurt by the COVID-19 pandemic^35^ and tend to experience increased vaccine hesitancy^36^.

### Second-reminder RCT

Participants who did not schedule their vaccine appointment a few days after the first reminder may have forgotten about it, been procrastinating or been more hesitant than those who got vaccinated. We conducted the second RCT to study the effect of sending these participants a second text reminder. On the second reminder date, we randomized eligible participants at a 6:1 ratio to receive another text message at 15:00 h that reminded them of vaccine availability and providing easy access to the scheduling website (the follow-through reminder arm) or to not receive the text message (the holdout arm).

To harness other psychological principles to motivate people to act, we randomized participants within the follow-through reminder arm to receive one of six messages that leveraged additional behavioural insights (‘Design of the second-reminder RCT’ in Methods). Following the preregistration, we present only the average effect of all text reminders combined relative to the holdout arm.

Our analysis includes 67,092 participants (43.5% male, 52.6% white (excluding Hispanic or Latino), average age = 73.7, s.d. = 10.0). Study arms were well-balanced on demographic characteristics (Extended Data Table 3).

Getting a second reminder increased participants’ likelihood of scheduling the first-dose appointment within six days by 1.65 percentage points (53.36%) and obtaining the first dose at UCLA Health within four weeks by 1.06 percentage points (17.23%), relative to the 3.10% appointment rates and 6.16% vaccination rates in the holdout arm (Extended Data Table 4). All reminder types boosted appointments and vaccinations (Extended Data Table 4). Although small, these effects are noteworthy, as they are documented within a more hesitant population (as participants in the second RCT had not scheduled an appointment after two notifications and had been eligible for COVID-19 vaccines in California for some time).

## Effect on vaccination anywhere

Because the text reminders made eligibility at UCLA Health salient and reduced barriers to appointment scheduling at UCLA Health, we have focused on appointments and vaccinations at UCLA Health as our outcome measures. We also investigated the effect of receiving a text reminder on whether participants received the first dose inside or outside UCLA Health (hereafter, ‘anywhere’) within four weeks of getting a reminder (Supplementary Information section 1.5).

For the first RCT, we find that reminders increased vaccinations anywhere by 2.1 percentage points, relative to a baseline of 31.85% in the holdout arm (OLS regression, *B* = 0.021, s.e. = 0.004, *P* < 0.001, *n* = 93,354) (Supplementary Table 22). In addition, adding (versus not) the ownership language increased vaccinations anywhere by an additional 0.9 percentage points (OLS regression, *B* = 0.009, s.e. = 0.003, *P* = 0.010 without multiple comparison adjustment and *P* = 0.020 with a Holm–Bonferroni correction^37^, *n* = 74,605) (Supplementary Table 22). The fact that the effect of receiving one reminder on vaccinations at any location could last one month is notable, considering that participants may have been exposed to numerous sources of communication about the vaccine during this period.

Receiving a second reminder increased vaccination rates anywhere by 1.0 percentage points two weeks after the second reminder date (OLS regression, *B* = 0.010, s.e. = 0.004, *P* = 0.008, *n* = 67,092) (Supplementary Table 24), relative to a baseline of 12.04% in the holdout arm. Although this effect was not statistically significant at four weeks (OLS regression, *B* = 0.007, s.e. = 0.004, *P* = 0.127, *n* = 67,092) (Supplementary Table 23), sending a second text reminder can still contribute to accelerating vaccinations and avoiding unnecessary infections. It is also worth noting that, had we designed the reminders to remove barriers to getting vaccinated at a broad set of locations (rather than focusing on UCLA Health), our reminders might have exhibited larger effects on vaccination anywhere.

## Vaccination intentions versus actual uptake

To inform policy, researchers often use surveys of intentions to evaluate the effectiveness of interventions aimed at encouraging vaccine uptake^3,27,28^. Given that intentions do not always reflect real behaviours^7^, we tested how the interventions deployed in our first RCT affected vaccination intentions and explored whether hypothetical responses would match actual behavioural responses.

We ran three preregistered experiments on Amazon’s Mechanical Turk and Prolific Academic: two concurrently to the first RCT in February 2021 and one as a replication in April 2021 (total *n* = 3,181). We randomized participants to receive one of the four reminders from the first RCT, asking about their intentions to get vaccinated using different questions on a seven-point scale (‘Procedures for online experiments’ in Methods). In contrast to the patterns observed in the first RCT, the video intervention resulted in a small—but statistically significant—increase in people’s self-reported interest in getting the vaccine; however, we found no evidence that adding ownership language increased vaccination intentions (Extended Data Table 5).

The discrepancy between laboratory and field data (Extended Data Table 6) is unlikely to be driven by differences in political attitudes between samples^38^, as the aforementioned findings about video and ownership interventions generally held both for those who self-identified as ‘Democrat’ and as ‘Republican’ online (Extended Data Table 5). One potential explanation for these discrepant findings is that, although we could require all online participants to watch the video, less than 21% of the participants in the first RCT opted to watch it (Supplementary Information section 1.3.4), possibly because of being too busy or active avoidance of information^39^. Another possibility is that COVID-19 vaccine intentions were harder to change outside of a controlled online experiment, where various sources of information compete for people’s attention. As for the lack of evidence that ownership language affected vaccination intentions, it could be that individuals did not anticipate the motivating power of such language in hypothetical settings. Whereas the differences in sample characteristics and measurement (Extended Data Table 6) do not allow us to pinpoint the drivers of the discrepancy between our online studies and the first RCT, these results suggest that hypothetical responses to behavioural nudges should be taken with caution.

## Discussion

Our research highlights that behavioural science insights can increase and speed up COVID-19 vaccinations at close-to-zero marginal cost. Text-based reminders designed to overcome barriers to scheduling can effectively encourage vaccinations across different demographic groups, with effects persisting for at least eight weeks. These effects are heightened when follow-through reminders leverage psychological ownership, making people feel that a dose of the vaccine belongs to them. However, we find no evidence that combining reminders with a video-based information intervention further increases vaccination, which suggests that more work is needed to uncover when information interventions can help to overcome vaccine hesitancy. Additional analyses of our RCT sample reveal that only about 10% of participants did not keep or show up for their first-dose appointment, and approximately 90% of participants who received the first dose at UCLA Health scheduled their second dose (Supplementary Information section 1.6). Thus, the biggest barrier to increasing COVID-19 vaccinations is getting participants to schedule the first-dose appointment.

Our research has implications for enhancing the uptake of life-saving vaccines in general, as it highlights the power of making vaccination easy and eliciting feelings of ownership over the vaccine. Although promoting vaccinations at scale requires a multifaceted approach, our findings suggest that behavioural nudges could be an important strategy to consider. If sent to all 263 million adults in the USA^40^, and assuming the same absolute effect size observed in our first RCT would hold for the 60% of US adults who did not immediately obtain the vaccine^41^, our follow-through reminders could result in 3.31–5.68 million extra people getting vaccinated within a month of the reminder. This estimated range is based on the average effect of receiving the first reminder on vaccination rates anywhere (that is, 60% × 263 million × 2.1 percentage points) versus at UCLA Health (60% × 263 million × 3.6 percentage points). Similarly, reminders with the ownership framing would motivate 1.42–1.74 million extra people to get vaccinated than reminders without such framing (that is, 60% × 263 million × 0.9 percentage points–60% × 263 million × 1.1 percentage points).

The insights from this work could inform strategies to motivate health-related behaviours more broadly, such as scheduling preventive care tests or participating in health-related programs. To that end, the discrepancy observed between our RCTs and online studies highlights the value of pilot-testing interventions in the field before deploying them at scale. As policymakers, public health experts and organizations strive to develop communication strategies to promote health-related behaviours, we hope that the effective interventions documented in our research—and behavioural science more generally—can become part of their toolbox.

## Methods

For RCTs, we predetermined the end date of enrolment for analyses reported herein, but we could not predetermine sample size by the enrolment deadline owing to uncertainty about how many UCLA Health participants would satisfy inclusion and exclusion criteria. We preregistered data-analysis plans contingent on the actual sample size on the basis of power analysis. We used power analysis to predetermine sample sizes for online experiments. RCTs and online experiments were randomized, and investigators were blinded to allocation during experiments.

### Ethics approval

This research was deemed to comply with all relevant ethical regulations. The Institutional Review Board at the UCLA approved the protocols of our randomized controlled trials (reference number 21-000268) and determined that a waiver of informed consent was appropriate. All online experiments and the vaccination intention survey were conducted under approval of the Institutional Review Board at Carnegie Mellon University (reference number IRBSTUDY2015_00000482), and informed consent was obtained from all online study participants as part of the enrolment process.

### Setting for the RCTs

We conducted the RCTs in partnership with UCLA Health, a large integrated academic health system in California. Extended Data Table 7 provides a comparison of demographic characteristics and vaccination rates between our RCT sample, UCLA Health primary and specialty care attributed patient population, Los Angeles County and California.

### Enrolment and eligibility for RCTs

Starting from 19 January 2021, UCLA Health invited primary and speciality care attributed patients who were eligible for the COVID-19 vaccine at the time to get vaccinated. UCLA Health followed the national Advisory Committee on Immunization Practices as well as state and county guidelines to determine patient COVID-19 vaccine eligibility phasing. Considering the large volumes of eligible patients in each phase, UCLA Health developed a risk model that incorporates clinical and social risk to subprioritize within each phase. According to this model, UCLA Health sent invitations to eligible patients in batches over time to guarantee enough vaccine supply for invited patients. The size of the batch was decided daily on the basis of (1) available doses, (2) available appointment slots and (3) expected appointment rate. If UCLA Health identified a patient as having already obtained the vaccine inside or outside UCLA Health when it was their turn to be invited, the health system did not send the invitation to that patient.

On the first reminder date, patients were automatically enrolled into the first RCT and became participants if they (1) had a SMS-capable telephone number, (2) had not scheduled the first-dose COVID-19 vaccination appointment at UCLA Health and (3) had not obtained the first dose anywhere by the end of the day before the first reminder date, according to the latest California Immunization Registry (CAIR) records UCLA Health could access as well as UCLA Health’s internal records. The earliest first reminder date was 1 February 2021.

On the second reminder date, patients were automatically enrolled in the second RCT and became participants if they (1) had a SMS-capable telephone number, (2) had not scheduled the first-dose COVID-19 vaccination appointment at UCLA Health and (3) had not obtained the first dose anywhere by the end of the day before the second reminder date. The earliest second reminder date was 9 February 2021.

Figure 1 shows the timeline, eligibility and randomization of the two RCTs. For both RCTs, participants within each batch were randomized at the individual level to treatments according to the design detailed in ‘Design of the first-reminder RCT’ and ‘Design of the second-reminder RCT’. Enrolment was conducted by the UCLA Health Office of Population Health and Accountable Care. Random assignment to interventions was performed by UCLA Health statisticians blind to the hypotheses and interventions using a computerized random number generator.

### Design of the first-reminder RCT

We randomly assigned participants following simple randomized procedures at a 4:1 ratio to either the follow-through reminder arm, in which they received a reminder at 15:00 h on the first reminder date, or the holdout arm, in which they received no reminders. All reminders were designed to nudge individuals to schedule their vaccination appointments by (1) making vaccination top of mind to curb forgetfulness, and (2) providing the direct link to the scheduling website to reduce friction and increase convenience. The basic reminder read ‘UCLA Health: [participant’s first name], you can get the COVID-19 vaccine at UCLA Health. Make a vaccination appointment here: uclahealth.org/schedule.’

We nested a 2 × 2 factorial design within the follow-through reminder arm. The first factor was whether or not the reminder sought to enhance participants’ feelings of psychological ownership over the vaccine to amplify their desire to obtain their vaccine (ownership intervention). The ownership intervention added language to the reminder to make participants feel as if the vaccine was already theirs. The ownership reminder read ‘UCLA Health: [participant’s first name], a COVID-19 vaccine has just been made available to you at UCLA Health. Claim your dose today by making a vaccination appointment here: uclahealth.org/schedule.’

The second factor was whether or not the reminder linked to a video that was designed to shift vaccination intentions by providing information about COVID-19 and the authorized vaccines (video intervention). The video intervention was based on a survey of the vaccine hesitancy literature^3,31,32,36^ as well as a survey that we conducted in January 2021 with California residents (as described in ‘Vaccination intention survey’). The video (Supplementary Video 1) first highlighted the pandemic as a challenge, providing statistics on infections and ease of transmission. It then proposed the vaccine as an easy and safe solution, providing information about its effectiveness. The basic reminder with video read ‘UCLA Health: [participant’s first name], you can get the COVID-19 vaccine at UCLA Health. Please watch this important 2 min video: [link]. Make a vaccination appointment here: uclahealth.org/schedule.’

In the ownership reminder with video sub-arm, the reminder contained both the ownership and video interventions and read: ‘UCLA Health: [participant’s first name], a COVID-19 vaccine has just been made available to you at UCLA Health. Please take 2 simple steps: 1. Watch this important 2 min video: [link]. 2. Claim your dose today by making a vaccination appointment here: uclahealth.org/schedule.’

In all sub-arms, participants whose preferred language was Spanish received the text reminder (and the video (Supplementary Video 2), in the relevant cases) in Spanish. Participants within the follow-through reminder arm were randomly assigned following simple randomization procedures to one of these four sub-arms with an equal probability.

### Design of the second-reminder RCT

Eight days after the initial notification, eligible participants were enrolled in the second RCT. They were randomized following simple randomization procedures at a 6:1 ratio to the follow-through reminder arm, in which another text reminder was sent at 15:00 h on the second reminder date, or the holdout arm with no reminders. Randomization was independent between the first and second RCTs (Supplementary Information section 1.1). Similar to the first RCT, all text reminders in the second RCT heightened the salience of vaccine availability (so as to combat forgetfulness) and provided the direct link to the appointment scheduling website (so as to increase convenience).

We nested a 2 × 3 factorial design within the follow-through reminder arm, in which we leveraged behavioural insights to motivate people to schedule a vaccination appointment via different messaging. The first factor varied whether the reminder emphasized prosocial (versus personal) benefits of getting vaccinated^42,43^. The second factor manipulated whether the reminder highlighted the exclusivity of having early access to the vaccine (early access framing), whether it framed the act of obtaining the vaccine as an opportunity to chart a new path forward (fresh start framing) or neither. The early access framing sought to leverage the principle of scarcity to increase vaccine demand^44,45^, as vaccination was still exclusive at the early stage of distribution (January–February 2021). The fresh start framing was inspired by previous work showing that people are motivated to take actions at new beginnings^46,47^. Here, we tested whether framing getting the vaccine as an opportunity to chart a new path forward for participants themselves or society could mobilize participants to get inoculated.

Specifically, the basic self/prosocial reminders read ‘UCLA Health: [participant’s name], to protect (yourself/your family, friends, and community), make your COVID-19 vaccine appointment here today: uclahealth.org/schedule.” The early access self/prosocial reminders read ‘UCLA Health: [participant’s name], you are one of few Americans who have early access to the COVID-19 vaccine based on national guidelines. Take this opportunity to protect (yourself/your family, friends, and community who may not have this access yet). Make your vaccine appointment here today: uclahealth.org/schedule.’ The fresh start self/prosocial reminder read ‘UCLA Health: [participant’s name], (the past year has been tough/the past year has been tough for many). Now, the COVID-19 vaccine can offer the promise of a fresh start. Take this opportunity to protect (yourself/your family, friends, and community) and (chart a new path forward/help our nation chart a new path forward). Make your vaccine appointment here today: uclahealth.org/schedule.’ The content in parentheses differed between the personal and prosocial messaging conditions. Participants within the follow-through reminder arm were randomly assigned following simple randomization procedures to one of these six sub-arms with an equal probability.

### Analyses and exclusion criteria of RCTs

All analyses and exclusion criteria follow the preregistrations. We focus on participants enrolled in either RCT by 23 February 2021. This sample consists of participants eligible to get vaccinated at UCLA Health from 19 January to 22 February 2021, including participants at or above 65 years old, participants with any transplant and high-risk participants with qualifying pre-existing conditions. We report results using data extracted on 25 May 2021. We excluded participants who were enrolled in the first (second) RCT but either scheduled a vaccination appointment at UCLA Health by 15:00 h on their corresponding first (second) reminder date or obtained a COVID-19 vaccine somewhere before their corresponding first (second) reminder date according to the latest appointment and vaccination records UCLA Health could access on 25 May 2021. These participants could not have been motivated to schedule or obtain the first dose by our text reminders; thus, excluding them allows us to more accurately estimate the effect of our interventions on participants who could benefit from receiving our interventions. We additionally excluded participants under 18 years old as we only applied for the permission of the Institutional Review Board to analyse data about adult participants. The proportion of participants who were excluded in the analysis stage did not statistically significantly differ across conditions (Supplementary Table 1), and our results are robust if we conduct intent-to-treat analyses involving all participants who were enrolled in the RCTs by 23 February 2021 (Supplementary Information section 1.5).

For the first RCT, our preregistered analysis about participants enrolled by 23 February 2021 aimed to investigate (1) the average effect of sending a follow-through reminder; (2) whether all reminder types would outperform the holdout arm; (3) the effect of adding the video intervention to the reminder; (4) the effect of adding the ownership intervention; and (5) whether the aforementioned effects would differ between participants who received versus did not receive the influenza vaccine in either of two recent influenza seasons.

For the second RCT, our preregistered analysis about participants enrolled by 23 February 2021 aimed to investigate (1) the average effect of sending a second follow-through reminder and (2) whether all reminder types outperformed the holdout arm. Because we were uncertain about how many people would be enrolled in the second RCT by 23 February 2021, we preregistered to not compare sub-arms to each other with this data. Supplementary Information sections 1.3 and 1.4 describe for the scope of analyses we plan to conduct once the full data collection has been completed about all participants ever enrolled in our RCTs from the beginning of the trials until UCLA Health stops sending out COVID-19 vaccine invitations.

### Outcome measures for RCTs

Our preregistered primary outcome measure indicates whether participants scheduled a vaccination appointment for the first dose of COVID-19 vaccine at UCLA Health within six days of the first (second) reminder date (specifically, from 15:00 h on the first (second) reminder date to 23:59 h on the fifth day following the first (second) reminder date.). We preregistered this time window because UCLA Health targeted additional outreach efforts to participants who had not scheduled their vaccination appointment six days after the second reminder date and we wanted to use a consistent time window for the two RCTs. The results are robust to extending the time window to one month (Supplementary Tables 22 and 23). Our secondary outcome measure in this Article is whether participants obtained the first dose of COVID-19 vaccine at UCLA Health within four weeks of the first (second) reminder date. We chose this window because UCLA Health generally only allowed participants to schedule an appointment for less than four weeks ahead. Consistent with this practice, 96.25% of the first-dose appointments made by participants in the analysis sample of the first RCT occurred within four weeks from the day they were scheduled. In the preregistrations, we listed additional secondary outcome variables; we explain why we did not focus on these in this Article in Supplementary Information section 1.2.

### Procedures for online experiments

We ran two preregistered online experiments in February 2021, concurrently with the randomized controlled trials. In addition, we ran a preregistered replication experiment online in April 2021, when all US adults had become eligible to receive the vaccine.

In the February 2021 experiments, we instructed participants to imagine becoming eligible for the COVID-19 vaccine and receiving a text message from their healthcare provider encouraging them to get vaccinated. We randomly assigned participants to read one of the four reminders from the first RCT. Participants in the ‘video’ conditions were also instructed to watch the video. After reading the message, participants indicated their likelihood of scheduling a vaccination appointment: ‘How likely would you be to schedule a vaccination appointment after receiving this message from your healthcare provider?’ (1, not at all likely, to 7, very likely). They also rated the persuasiveness of the text message (1, not at all persuasive, to 7, very persuasive). To check whether the messages containing ownership language increased feelings of psychological ownership over the vaccine as we intended, we asked participants, ‘To what extent does the text message make you feel that the COVID-19 vaccine is already yours?’ (1, not at all, to 7, very much)^30^. To understand how the video may have changed viewers’ perceptions and beliefs, we measured participants’ beliefs about the prevalence of COVID-19, worry about spreading the virus, perceived vaccine effectiveness, anticipated regret for not getting the vaccine and trust in the vaccine (Supplementary Information section 2 for questions and results).

The April 2021 experiment used identical procedures but adopted additional measures of vaccination intentions to test whether findings in our February 2021 studies are robust to different ways of soliciting intentions. For this purpose, we randomized whether participants answered questions in the same hypothetical manner as in the February studies, or responded to questions with a less hypothetical framing. Participants randomized to answer the hypothetical version were asked ‘How likely would you be to schedule a vaccination appointment after receiving this message from your healthcare provider?’ (1, not at all likely, to 7, very likely) and ‘How much would you want the vaccine after receiving this message from your healthcare provider?’ (1, not at all, to 7, very much). These two questions were highly correlated (*r* = 0.94, *P* < 0.001) and aggregated into a composite. Participants randomized to answer the less hypothetical version of the intention questions were asked ‘How likely are you to schedule a vaccination appointment today after receiving this message from your healthcare provider?’ (1, not at all likely, to 7, very likely) and ‘How much do you want the vaccine now, after receiving this message from your healthcare provider?’ (1, not at all, to 7, very much). These two measures were highly correlated (*r* = 0.93, *P* < 0.001) and averaged into a composite. All participants also rated the persuasiveness of the message they read, using the same measure used in the February studies (Supplementary Information section 3).

### Sample for online experiments

We recruited participants from Amazon’s Mechanical Turk (MTurk) and Prolific Academic (Prolific) who had not received a COVID-19 vaccine or scheduled a first-dose vaccination appointment at the time of the study. To be assigned to treatment, participants had to first pass a Captcha and an attention check question. To be included in the analysis, participants had to complete our preregistered dependent variables and not report having technical problems with the video. Considering these criteria, our first February 2021 online experiment consists of 1,163 participants. Our second February 2021 online experiment consists of 840 participants recruited from Prolific who satisfied similar criteria as those in the first online experiment, except that we additionally required that they did not report having taken a similar survey on MTurk. In both experiments, we attempted to recruit a balanced sample of individuals who self-reported as Democrat or Republican to test the generalizability of our findings (Supplementary Information section 2.1 for recruitment detail). Participants received US$0.90 on MTurk and US$1.10 on Prolific for completing our 6-min survey. Across the two February online experiments, our sample consists of 2,003 participants (47.1% male, 71.8% white (excluding Hispanic or Latino), 51.8% Democrat, average age = 37.9, s.d. = 13.4).

For our April 2021 online experiment, we recruited participants on MTurk and Prolific using the same eligibility criteria as the second online experiment. Participants on MTurk received US$0.90 or US$1.00 (we boosted the pay to US$1.00 on the third day of data collection to attract more respondents) and those on Prolific received US$1.10 for completing our 6-min survey. Our sample consists of 1,178 participants (44.9% male, 71.6% white (excluding Hispanic or Latino), 40.8% Democrat, average age = 36.7, s.d. = 12.0).

### Vaccination intention survey

To design the video used in our first RCT, we ran a survey in January 2021 involving 515 residents of California recruited on MTurk and Prolific (49.3% male, 42.9% white (excluding Hispanic or Latino), 70.9% Democrat, average age = 33.9, s.d. = 12.7). Participants received US$1.00 on MTurk or US$1.20 on Prolific for completing our 9-min survey. We asked participants to consider the authorized vaccines (Pfizer and Moderna) when taking the survey. We elicited their vaccination intentions by asking ‘If one of the COVID-19 vaccines were available to you today, would you get the vaccine?’^41^. Participants chose one from four options: ‘Definitely would get the vaccine’, ‘Probably would get the vaccine’, ‘Probably would not get the vaccine’ and ‘Definitely would not get the vaccine’. We then elicited participants’ beliefs and perceptions about COVID-19 and the vaccines. Specifically, we measured beliefs about infection likelihood with and without the vaccine and the severity of COVID-19. We collected feelings of vulnerability to COVID-19, fear of infection, worry of transmitting COVID-19 to others, anticipated regret for not getting the vaccine and trust in the vaccine. We compared answers to these questions among people who reported that they definitely would get the vaccine versus those feeling more uncertain. Supplementary Information section 5 describes all variables and results.

### Methods of investigating intentions versus actual uptake

In Extended Data Table 6, we report statistics about the estimated effects of adding ownership language and a video-based information intervention to a reminder on vaccination intentions (based on online experiments) versus actual vaccine uptake (based on the first RCT). The statistics we report include the 95% confidence interval, the absolute value of Cohen’s *d* or *h*, and *η*_*p*_^2^ of each estimated effect. To calculate Cohen’s *h* for the binary outcomes measured in the first RCT, we use 2 × arcsine √*P*_with an intervention_ − 2 × arcsine √*P*_without an intervention_^48^ in which √*P*_with an intervention_ represents the percentage of participants who scheduled an appointment for (or obtained) the first dose at UCLA Health within six days (or within four weeks) of the first reminder date among those who received a text reminder containing a given intervention and √*P*_without an intervention_ represents the percentage among participants who received a text reminder without that intervention. To calculate *η*_p_^2^ for the online experiments and the first RCT, we use *η*_p_^2^ = *F* × *df*_numerator_/(*F* × *df*_numerator_ + *df*_denominator_)^49^ in which the *F* value and numerator and denominator degrees of freedom came from the OLS regressions reported in Supplementary Tables 5, 39.

### Reporting summary

Further information on research design is available in the Nature Research Reporting Summary linked to this paper.

## Online content

Any methods, additional references, Nature Research reporting summaries, source data, extended data, supplementary information, acknowledgements, peer review information; details of author contributions and competing interests; and statements of data and code availability are available at 10.1038/s41586-021-03843-2.

### Supplementary information

Supplementary Information This file contains Supplementary text, Supplementary Methods, Supplementary Figures 1 - 5, Supplementary Tables 1 – 41 and Supplementary References.

Reporting Summary

Supplementary Video 1 **Video Addressing Vaccine Hesitancy (English Version)**. This is the English version of the video we developed to address COVID-19 vaccine hesitancy. It was used in the first RCT (presented to patients who did not indicate a preference for Spanish) and online experiments. We based this video on a literature review of vaccine hesitancy and our January 2021 survey of California residents (*N*=515; 'Vaccination Intention Survey' in Methods), which allowed us to identify common misconceptions about COVID-19 and authorized vaccines.

Supplementary Video 2 **Video Addressing Vaccine Hesitancy (Spanish Version)**. This is the Spanish version of the video we developed to address COVID-19 vaccine hesitancy. It was used in the first RCT (presented to patients who indicated a preference for Spanish). We based this video on a literature review of vaccine hesitancy and our January 2021 survey of California residents (*N*=515; `Vaccination Intention Survey' in Methods), which allowed us to identify common misconceptions about COVID-19 and authorized vaccines. This video is identical to Supplementary Video 1 except that the voice and text here are in Spanish. 

## Figures and Tables

**Extended Data Table 1:**
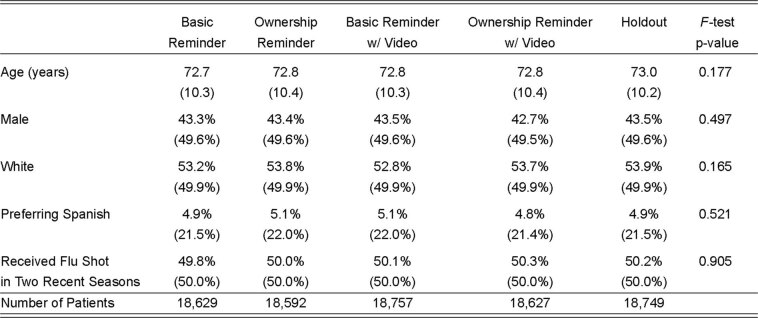
Randomization check of the first RCT

Average and s.d. (in parentheses) of participant demographic variables in each condition of the first RCT, the *P* value of a two-sided *F*-test for joint significance across five conditions for each variable, and the number of participants in each condition. The demographic variables reported here include participant age (‘Age (years)’), whether a participant was a man (versus a woman, other or unknown; ‘Male’), whether a participant was white (excluding Hispanic or Latino; ‘white’), whether Spanish was a participant’s preferred language (‘Preferring Spanish’), and whether a participant received the influenza vaccine shot in either of two recent influenza seasons (‘Received Flu Shot in Two Recent Seasons’). We predicted each demographic variable using an OLS regression as a function of indicators for the four conditions within the follow-through reminder arm (the holdout arm as the reference group) with robust standard errors to correct for heteroscedasticity. A two-sided *F*-test was then conducted for the *β* coefficients from each regression to compare the overall significance across conditions for a given demographic variable. If a given *P* value is greater than 0.05, it means the corresponding *F*-test does not allow us to reject the null hypothesis that all five conditions in the first RCT have the same value for the corresponding demographic variable.

**Extended Data Table 2:**
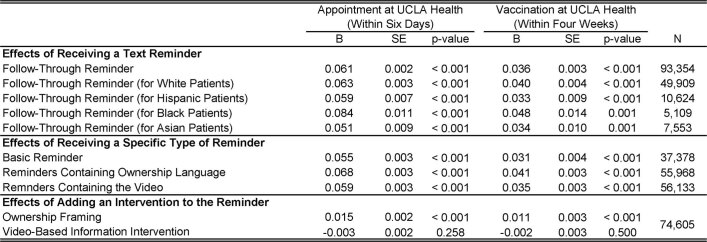
Regression-estimated effects of text reminders on appointments and vaccinations at UCLA Health in the first RCT

Regression-estimated effects of receiving a text reminder (altogether or broken down by reminder type) as well as the effect of adding an intervention (either ownership framing or video-based information intervention) to the reminder on appointments and vaccinations at UCLA Health in the first RCT. The two outcome measures are whether participants scheduled an appointment to get the first dose of the COVID-19 vaccine at UCLA Health from 15:00 h on the first reminder date to 23:59 h on the fifth day following the first reminder date  (‘Appointment at UCLA (Within Six Days)’) and whether participants obtained the first dose of the COVID-19 vaccine at UCLA Health within four weeks of the first reminder date (‘Vaccinated at UCLA (Within Four Weeks)’). Supplementary Tables 3–5, 11, 12 provide results of complete OLS regressions that are used to estimate the effects reported here. All *P* values are two-sided without multiple comparison adjustments. For the effect of adding ownership framing to the reminder, the *P* value with a Holm–Bonferroni correction is < 0.002 for both outcome measures. Supplementary Information section 1.3 describes how we handle multiple comparisons. ‘N’ refers to the number of observations in each regression.

**Extended Data Table 3:**
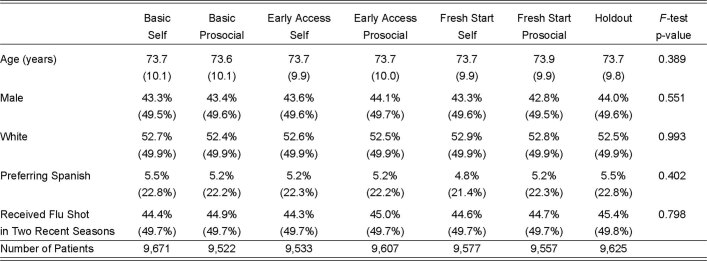
Randomization check of the second RCT

Average and s.d. (in parentheses) of participant demographic variables in each condition of the second RCT, the *P* value of a two-sided *F*-test for joint significance across seven conditions for each variable, and the number of participants in each condition. The demographic variables reported here include participant age (‘Age (years)’), whether a participant was a man (versus a woman, other or unknown; ‘Male’), whether a participant was white (excluding Hispanic or Latino; ‘White’), whether Spanish was a participant’s preferred language (‘Preferring Spanish’), and whether a participant received the influenza vaccine in either of two recent influenza seasons (‘Received Flu Shot in Two Recent Seasons’). We predicted each demographic variable using an OLS regression as a function of indicators for the six conditions within the follow-through reminder arm (the holdout arm as the reference group) with robust standard errors to correct for heteroscedasticity. A two-sided *F*-test was then conducted for the *β* coefficients from each regression to compare the overall significance across conditions for a given demographic variable. If a given *P* value is greater than 0.05, it means the corresponding *F*-test does not allow us to reject the null hypothesis that all seven conditions in the second RCT have the same value for the corresponding demographic variable.

**Extended Data Table 4:**
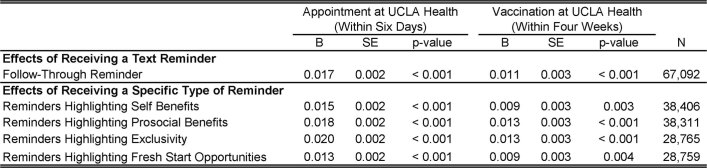
Regression-estimated effects of text reminders on appointments and vaccinations at UCLA Health in the second RCT

Regression-estimated effects of receiving a text reminder (altogether or broken down by reminder type) on appointments and vaccinations at UCLA Health in the second RCT. The two outcome measures are whether participants scheduled an appointment to get the first dose of the COVID-19 vaccine at UCLA Health from 15:00 h on the second reminder date to 23:59 h on the fifth day following the second reminder date  (‘Appointment at UCLA (Within Six Days)’) and whether participants obtained the first dose of the COVID-19 vaccine at UCLA Health within four weeks of the second reminder date (‘Vaccinated at UCLA (Within Four Weeks)’). Supplementary Tables 14, 15 provide results of complete OLS regressions that are used to estimate the effects reported here. All *P* values are two-sided without multiple comparison adjustments. ‘N’ refers to the number of observations in each regression.

**Extended Data Table 5:**
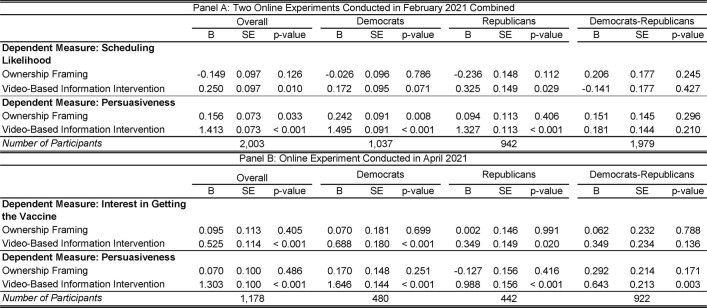
Regression-estimated effects of ownership framing and video-based information intervention in online experiments

Regression-estimated effects of adding ownership framing and a video-based information intervention to a text reminder in online experiments, across all participants as well as broken down by political party. **a**, Combined results for the two online experiments conducted in February 2021. These experiments assessed participants’ self-reported likelihood of scheduling an appointment for the COVID-19 vaccine after getting a text message from their healthcare provider (‘Scheduling Likelihood’) as well as how persuasive participants found the message to be (‘Persuasiveness). **b**, Results for the online experiment conducted in April 2021. This experiment assessed participants’ interest in getting the COVID-19 vaccine after getting a text message from their healthcare provider (‘Interest in Getting the Vaccine’) as well as how persuasive participants found the message to be (‘Persuasiveness’). For **a**, **b**, we separately report the effects for all participants included in the sample, participants who identified themselves as Democrat, and participants who identified themselves as Republican, as well as the differences in the effects between Democrats and Republicans. Supplementary Tables 25, 28, 35,38 provide results of complete OLS regressions that are used to estimate the effects reported here. All *P* values are two-sided without multiple comparison adjustments.

**Extended Data Table 6:**
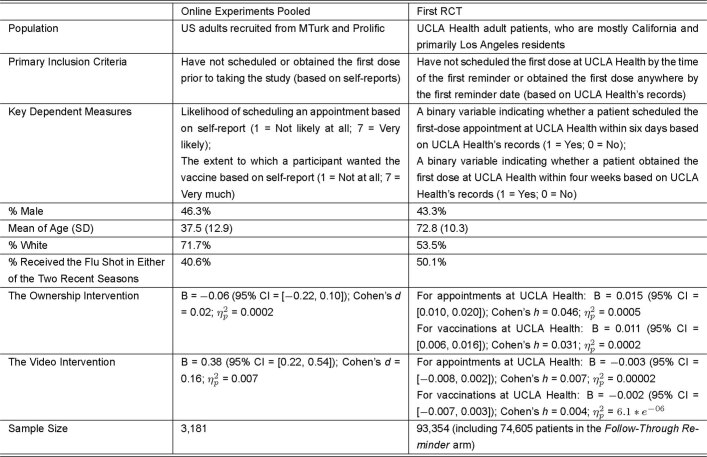
Comparison between the first RCT and online experiments

Comparison of the first RCT and online experiments (pooled across three experiments) in their primary inclusion criteria, key dependent measures, sample characteristics (including the percentage of men, the average and s.d. of participant age, the percentage of participants who were white (excluding Hispanic or Latino), and the percentage of participants who received the influenza shot in either of two recent influenza seasons), and statistics about the estimated effects of adding ownership language and a video-based information intervention to a reminder (including the 95% confidence interval, the absolute value of Cohen’s *d* or *h*, and *η*_*p*_^2^ of each estimated effect). The extent to which a participant wanted the vaccine was only measured in the online experiment conducted in April 2021; for participants in this experiment, the outcome measure was the average of their responses to the two questions listed above. The methods for calculating Cohen’s *h* and *η*_p_^*2*^ are described in ‘Methods of investigating intentions versus actual uptake’ in Methods. The values used to calculate *η*_p_^2^ came from the OLS regressions reported in Supplementary Tables 5, 39. Supplementary Information section 4 provides a discussion about this table.

**Extended Data Table 7:**
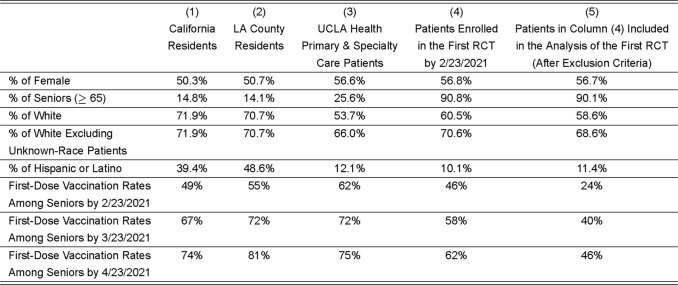
Comparison between UCLA Health participants, California residents and Los Angeles County residents

Comparison of demographic characteristics and vaccination rates across five populations: (1) California residents, (2) Los Angeles (LA) County residents, (3) all UCLA Health primary care and speciality care attributed patients (about 600,000 in total as of 25 May 2021) regardless of whether they were invited to get vaccinated at UCLA Health, (4) participants enrolled in the first RCT by 23 February 2021 (*n* = 132,337), and (5) participants in our analysis of the first RCT (that is, participants who were enrolled in the first RCT by 23 February 2021 and were not further excluded from analyses based on our exclusion criteria) (*n* = 93,354). Demographic data of California residents and Los Angeles County residents were accessed from https://www.census.gov/quickfacts/CA and https://www.census.gov/quickfacts/losangelescountycalifornia, respectively, on 21 May 2021. The first-dose vaccination rates of California residents were accessed from https://covid19.ca.gov/vaccination-progress-data/\#progress-by-group on 21 May 2021. To calculate the first-dose vaccination rates of Los Angeles County residents, we divided the number of senior residents who had received at least one dose as of a given day by the number of senior residents in Los Angeles County. Both sets of statistics were accessed from http://publichealth.lacounty.gov/media/coronavirus/vaccine/vaccine-dashboard.htm on 21 May 2021. For UCLA Health participants, first-dose vaccination rates consider first-dose vaccinations obtained inside and outside UCLA Health. ‘% of White’ refers to the percentage of white alone (excluding a ‘mixed race’ self-identification) for California and Los Angeles County residents but includes this ‘mixed race’ identification for UCLA Health participants. Because some UCLA Health participants had an unknown race, we also report the percentage of white participants after excluding participants with an unknown race, to be more comparable to California and Los Angeles Country statistics (which do not have an unknown race category). Supplementary Information section 1.6 provides a discussion about this table.

## Data Availability

The two RCTs were pre-registered at clinicaltrials.gov (first-reminder RCT, https://clinicaltrials.gov/ct2/show/NCT04800965; second-reminder RCT, https://clinicaltrials.gov/ct2/show/NCT04801524). The three online experiments were preregistered at aspredicted.org (online experiment 1, https://aspredicted.org/blind.php?x=u2ng5c; online experiment 2, https://aspredicted.org/blind.php?x=ae3ci5; and online experiment 3, https://aspredicted.org/blind.php?x=7wf9er and https://aspredicted.org/blind.php?x=u82hy5). The data analysed in this Article about randomized controlled trials were provided by UCLA Health and contain protected health information. To protect participant privacy, we cannot publicly post individual-level data. Qualified researchers with a valuable research question and relevant approvals including ethical approval can request access to the de-identified data about these trials from the corresponding author. A formal contract will be signed and an independent data protection agency should oversee the sharing process to ensure the safety of the data. Data about our online experiments and vaccination intention survey are available at: https://osf.io/qn8hr/?view_only=cf7b2bc590054aee8c4a2bae99ef20c5.

## Code availability

The code to replicate the analyses and figures in the Article and its Supplementary Information is available at https://osf.io/qn8hr/?view_only=cf7b2bc590054aee8c4a2bae99ef20c5.
