# Supplementary material for: Behavioural nudges increase COVID-19 vaccinations
Source: Nature. 2021 Aug 2;597(7876):404–9. doi: 10.1038/s41586-021-03843-2 (PMC8443442; doi:10.1038/s41586-021-03843-2)
Supplement: Supplementary file 1 — This file contains Supplementary text, Supplementary Methods, Supplementary Figures 1 - 5, Supplementary Tables 1 – 41 and Supplementary References. [file 41586_2021_3843_MOESM1_ESM.pdf]

nature portfolio

https://doi.org/10.1038/s41586-021-03843-2

Supplementary information

Behavioural nudges increase COVID-19 vaccinations

In the format provided by the authors and unedited

Behavioral Nudges Increase COVID-19 Vaccinations
Supplementary Information

June 2021

Hengchen Dai[1*], Silvia Saccardo[2*], Maria A Han[3], Lily Roh[4], Naveen Raja[4], Sitaram Vangala[5], Hardikkumar Modi[6], Shital Pandya[6], Michael Sloyan[7], Daniel M Croymans[3]

1 Anderson School of Management, University of California, Los Angeles, Los Angeles, California, 90095. USA.

2 Department of Social and Decision Sciences, Carnegie Mellon University, Pittsburgh, Pennsylvania, 15213. USA

3 Department of Medicine, David Geffen School of Medicine, University of California, Los Angeles, Los Angeles, California, 90095. USA.

4 Office of Population Health and Accountable Care, University of California, Los Angeles, Los Angeles, California, 90095. USA.

5 Department of Medicine Statistics Core, David Geffen School of Medicine, University of California, Los Angeles, Los Angeles, California, 90024. USA.

6 Office of Health Informatics and Analytics, University of California, Los Angeles, Los Angeles, California, 90095. USA

7 Department of Information Services and Solutions, UCLA Health System, Los Angeles, California, 90095. USA

*The first two lead authors contributed equally to this work.
Correspondence and requests for materials should be addressed to Daniel M Croymans at dcroymans@mednet.ucla.edu.

Supplementary Information Table of Contents

1 Randomized Controlled Trials **6**
1.1 Additional Information About the Interventions and Sample 6
Table 1 - Sample Sizes Across Arms in Two RCTs 7
Table 2 - Randomization Independence Between Two RCTs 8
1.2 Additional Information About Outcome Measures and Control Variables 9
1.3 Analyses and Results About the First RCT 10
1.3.1 Pre-registered Scope of Analyses About Patients Enrolled by February 23, 2021 10
1.3.2 The Average Effect of Receiving a Text Reminder 11
Figure 1 - Kaplan-Meier Curves Reflecting the Proportion of Patients Who Had Scheduled the First Dose at UCLA Health by a Given Day After the First Reminder Date 12
Table 3 - Effects of Receiving a Follow-Through Text Reminder on Appointments and Vaccinations at UCLA Health 13
1.3.3 Comparing Different Types of Text Reminders with Holdout 14
Table 4 - Effects of Different Types of Reminders on Appointments and Vaccinations at UCLA Health 15
1.3.4 The Additive Effects of Ownership Language and Video-based Information Intervention 16
Table 5 - The Additive Effects of Ownership Language and Video on Appointments and Vaccinations at UCLA Health 17
1.3.5 The Heterogeneous Treatment Effect by Flu Vaccination Status 18
Table 6 - Effects of Receiving a Text Reminder, Broken Down by Prior Flu Vaccination Status 19
Table 7 - Effects of Basic Reminder (vs. Holdout), Broken Down by Prior Flu Vaccination Status 20
Table 8 - Effects of Ownership Messages (vs. Holdout), Broken Down by Prior Flu Vaccination Status 21
Table 9 - Effects of Video Messages (vs. Holdout), Broken Down by Prior Flu Vaccination Status 22
Table 10 - The Additive Effects of Ownership Language and Video, Broken Down by Prior Flu Vaccination Status 23
1.3.6 Generalizability Across Racial/Ethnic and Age Groups 24
Table 11 - Effects of Receiving a Text Reminder and Adding Ownership Language Among White Patients vs. Racial/Ethnic Minority Patients 25
Table 12 - Effects of Receiving a Text Reminder, Broken Down by the Three Most Common Racial/Ethnic Minority Subgroups 26
Table 13 - Effects of Receiving a Text Reminder and Adding Ownership Language, Broken Down by Whether or Not Patients Were at Least 65 Years Old 27
1.3.7 Pre-registered Scope of Analyses After Data Collection Has Been Completed 28
1.4 Analyses and Results about the Second RCT 29
1.4.1 Pre-registered Scope of Analyses about Patients Enrolled by February 23, 2021 29
1.4.2 The Average Effect of Receiving the Second Text Reminder 29
Table 14 - Effects of Receiving the Second Text Reminder on Appointments and Vaccinations at UCLA Health 30
1.4.3 Comparing Different Types of Second Text Reminders with Holdout 31

Table 15 - Effects of Different Types of Second Text Reminders on Appointments and Vaccinations at UCLA Health . . . 32
Figure 2 - Appointment and Vaccination Rates by Condition (Second RCT) . . . 33
1.4.4 Pre-registered Scope of Analyses After Data Collection Has Been Completed . . . 33
1.5 Robustness Checks . . . 35
Table 16 - Effects of Receiving a Text Reminder and Adding Ownership Language (Intent-to-Treat Analysis) . . . 36
Table 17 - Effects of Receiving the Second Text Reminder (Intent-to-Treat Analysis) . . . 37
Table 18 - Effects of Receiving a Text Reminder and Adding Ownership Language (Without Controls) . . . 38
Table 19 - Effects of Receiving the Second Text Reminder (Without Controls) . . . 38
Table 20 - Effects of Receiving a Text Reminder and Adding Ownership Language (Logistic Regressions) . . . 39
Table 21 - Effects of Receiving the Second Text Reminder (Logistic Regressions) . . . 40
Table 22 - Effects of Receiving a Text Reminder and Adding Ownership Language on Appointments at UCLA Health and Vaccinations Anywhere Within Four Weeks . . . 41
Table 23 - Effects of Receiving the Second Text Reminder on Appointments at UCLA Health and Vaccinations Anywhere Within Four Weeks . . . 42
Figure 3 - Kaplan-Meier Curves Reflecting the Proportion of Patients Who Had Obtained the First Dose Anywhere by a Given Day After the Second Reminder Date . . . 43
Table 24 - Effects of Receiving the Second Text Reminder on Vaccinations Anywhere Within Two Weeks . . . 44
1.6 Additional Information About UCLA Health Patients and Vaccination Patterns . . . 45
1.6.1 Representativeness of Our Study Sample . . . 45
1.6.2 Cancellation and No-Show Rates . . . 46
1.6.3 Scheduling Rates of the Second Dose . . . 46

2 Online Experiments Assessing Intentions and Mechanisms - February 2021 . . . 47
2.1 Method . . . 47
2.1.1 Participants . . . 47
2.1.2 Procedure . . . 48
2.1.3 Measures . . . 49
2.2 Results . . . 51
2.2.1 Scheduling Likelihood . . . 52
2.2.2 Message Persuasiveness . . . 52
Figure 4 - Likelihood of Scheduling and Persuasiveness by Message . . . 53
Table 25 - Effects of Text Messages on Likelihood of Scheduling and Persuasiveness . . . 54
2.2.3 Mechanism for the Ownership Language . . . 55
Table 26 - Effects of Ownership Language on Psychological Ownership and Ease of Getting Vaccinated . . . 55
2.2.4 Mechanisms for the Video . . . 56

Table 27 - Effects of Video on Beliefs and Perceptions . . . 57
2.2.5 Heterogeneous Treatment Effects . . . 58
Table 28 - Main Effects of Text Message on Scheduling Likelihood and Persuasiveness for Democrats and Republicans . . . 59
2.2.6 Separate Results for the First and Second Online Experiments Conducted in February 2021 . . . 60
Table 29 - Experiment 1: Effects of Text Messages on Likelihood of Scheduling and Persuasiveness 60
Table 30 - Experiment 1: Effects of Ownership Language on Psychological Ownership and Ease of Getting Vaccinated . . . 61
Table 31 - Experiment 1: Effects of Video on Beliefs and Perceptions . . . 62
Table 32 - Experiment 2: Effects of Text Messages on Likelihood of Scheduling and Persuasiveness 63
Table 33 - Experiment 2: Effects of Ownership Language on Psychological Ownership and Ease of Getting Vaccinated . . . 64
Table 34 - Experiment 2: Effects of Video on Beliefs and Perceptions . . . 65

3 Replication Online Experiment Assessing Intentions and Mechanisms in April 2021 66
3.1 Method . . . 66
3.1.1 Participants . . . 66
3.1.2 Procedure and Measures . . . 67
3.2 Results . . . 67
3.2.1 Interest in Getting the Vaccine . . . 68
3.2.2 Message Persuasiveness . . . 68
Figure 5 - Interest in Getting the Vaccine and Persuasiveness by Message and Measurement Methods . . . 70
Table 35 - Effects of Text Messages on Interest in Getting the Vaccine and Persuasiveness . . . 71
3.2.3 Mechanism for the Ownership Language . . . 72
Table 36 - Effects of Ownership Language on Psychological Ownership . . . 72
3.2.4 Mechanisms for the Video . . . 73
Table 37 - Effects of Video on Beliefs and Perceptions . . . 74
3.2.5 Heterogeneous Treatment Effects . . . 75
Table 38 - Effects of Text Messages on Interest in Getting the Vaccine and Persuasiveness for Democrats and Republicans . . . 76

4 Comparing the First RCT with Online Experiments 77
Table 39 - Effects of Text Messages on Vaccination Intentions Pooled Across Online Experiments 78

5 Online Survey Comparing Beliefs and Perceptions Across People with Different Vaccination Intentions 79
5.1 Method . . . 79
5.1.1 Participants . . . 79
5.1.2 Procedure and Measures . . . 79
5.2 Results . . . 82
Table 40 - Vaccination Intentions Overall and by Political Affiliation and Race/Ethnicity . . . 83

Table 41 - Comparisons of COVID-19-related Beliefs and Perceptions by Vaccination Intentions 84

1 Randomized Controlled Trials

1.1 Additional Information About the Interventions and Sample

In the *Basic Reminder with Video* condition, the link to the video directed patients to a survey where we embedded the video. A sample of the survey can be accessed here: https://tinyurl.com/f3hfwwsh. Patients could start and stop the video at any time. In the *Ownership Reminder with Video* condition, the first link also directed patients to a survey where we embedded the video. Readers can experience it here: https://tinyurl.com/nv9wktsf. Different from the *Basic Reminder with Video* condition, the survey in the *Ownership Reminder with Video* condition displayed a button below the video 60 seconds after patients landed in the survey. The button reiterated the first sentence and the second step in the text message, reading, *"A COVID-19 vaccine is waiting for you at UCLA Health. Claim your dose today by scheduling your appointment here."* The button redirected patients to https://uclahealth.org/schedule. The button was intended to make it easier for patients to take action and schedule an appointment.

In this paper, we follow our pre-registration to analyze patients whose first or second reminder date was no later than February 23 (see pre-registrations at: https://clinicaltrials.gov/ct2/show/NCT04800965 and https://clinicaltrials.gov/ct2/show/NCT04801524). Those patients were eligible for the COVID-19 vaccine because they were at or above 65 years old, had any transplant, or had high-risk conditions stipulated by the Centers for Disease Control and Prevention.

Per our pre-registration, we excluded patients who were enrolled in the first (second) RCT but either scheduled a vaccination appointment at UCLA Health by 3pm PST on their corresponding first (second) reminder date or obtained a COVID-19 vaccine somewhere before their corresponding first (second) reminder date according to the latest appointment and vaccination records UCLA Health could access on May 25, 2021. Ideally, we would have liked to completely exclude patients that satisfied our exclusion criteria at the enrollment process. However, we could not do so perfectly at the enrollment stage and had to further exclude people from analyses for two reasons. First, vaccination records, especially CAIR records about vaccinations obtained outside UCLA Health, were not up-to-date. To meet our pre-registered exclusion criteria, we excluded patients from analysis who had gotten the first dose before their first (second) reminder date according to the latest vaccination records (obtained on May 25, 2021). Second, because we had to randomize patients into conditions and send the contact list to our text-message vendor early in the morning on each reminder date, our enrollment process could only exclude patients who already scheduled the first-dose appointment at UCLA Health or obtained the first dose anywhere by 11:59pm the day before based on the records we had access to at the time of randomization. To meet our pre-registered exclusion criteria, we excluded from the analysis patients who had an outstanding first-dose appointment at UCLA Health by 3pm PST on the first (second) reminder date based on the latest appointment records (obtained on May 25, 2021). To summarize, most people that were excluded in our analysis stage are people who should have been excluded before enrollment but were not because we did not have full up-to-date data on their appointment scheduling or vaccination at time of randomization.

In addition, we also excluded a small number of patients who were were under 18 years old because, even though patients above 16 who were eligible for COVID-19 vaccine at UCLA Health were invited and enrolled in the RCTs, we only asked for an approval from the Institution of Review Board to analyze and report data based on patients who are at or above 18 years old. Note that only about 50 patients were excluded from

each RCT solely because of their age.

Importantly, the proportion of patients who were excluded in the analysis stage did not statistically significantly differ across conditions (see Supplementary Table 1).

Further, Supplementary Table 2 confirms that randomization was independent between the first and seconds RCTs. Note that some patients in the *Holdout* arm of the first RCT were assigned to the *Follow-Through Reminder* arm in the second RCT. The text message sent to these patients on their second reminder date was the first reminder they received from UCLA Health encouraging them to get the COVID-19 vaccine. But for simplicity, we refer to the text message sent on the second reminder date as the second text reminder for everyone.

Table 1: Sample Sizes Across Arms in Two RCTs

	Panel A: First RCT			
	Number of Patients Enrolled	Number of Patients Analyzed	Number of Patients Excluded	Exclusion Rate
Basic Reminder	26,497	18,629	7,868	29.69%
Ownership Reminder	26,473	18,592	7,881	29.77%
Basic Reminder w/ Video	26,472	18,757	7,715	29.14%
Ownership Reminder w/ Video	26,444	18,627	7,817	29.56%
Holdout	26,451	18,749	7,702	29.12%
	Panel B: Second RCT			
	Number of Patients Enrolled	Number of Patients Analyzed	Number of Patients Excluded	Exclusion Rate
Basic Self	14,671	9,671	5,000	34.08%
Basic Prosocial	14,669	9,522	5,147	35.09%
Early Access Self	14,669	9,533	5,136	35.01%
Early Access Prosocial	14,667	9,607	5,060	34.50%
Fresh Start Self	14,667	9,577	5,090	34.70%
Fresh Start Prosocial	14,667	9,557	5,110	34.84%
Holdout	14,665	9,625	5,040	34.37%

Legend: This table presents the number of patients who were randomly assigned to each condition upon enrollment (by February 23, 2021), the number of patients in each condition who were included in the analysis sample, the number of enrolled patients who were excluded from the analysis (see Section *RCT - Analyses and Exclusion Criteria* in Methods and Supplementary Information Section 1.1 for exclusion criteria), as well as the percentage of enrolled patients in each condition who were excluded from the analysis. Panel A displays the aforementioned statistics for the first RCT. Using an ordinary least squares regression with robust standard errors, we predicted whether each patient enrolled in the first RCT by February 23, 2021 was excluded from the analysis as a function of indicators for the four conditions within the *Follow-Through Reminder* arm (the Holdout arm as the reference group). A two-sided F-test was then conducted for the beta coefficients from this regression to compare the overall significance across conditions for exclusion rate. The p-value is 0.31, suggesting that we cannot reject the null hypothesis that all five conditions in the first RCT have the same exclusion rate. Panel B displays the aforementioned statistics for the second RCT. Using the same method to conduct a two-sided F-test for the second RCT, we obtained a p-value of 0.53, suggesting that we cannot reject the null hypothesis that all seven conditions in the second RCT have the same exclusion rate.

Table 2: Randomization Independence Between Two RCTs

	Random Assignment in the First RCT				
	Basic Reminder	Ownership Reminder	Basic Reminder w/ Video	Ownership Reminder w/ Video	Holdout
Basic Self	1,880	1,889	1,868	1,942	2,048
	14.24%	14.13%	14.38%	14.77%	14.54%
Basic Prosocial	1,912	1,869	1,835	1,906	1,968
	14.48%	13.98%	14.13%	14.50%	13.97%
Early Access Self	1,849	1,896	1,839	1,885	2,022
	14.00%	14.18%	14.16%	14.34%	14.36%
Early Access Prosocial	1,904	1,949	1,870	1,826	2,017
	14.42%	14.58%	14.40%	13.89%	14.32%
Fresh Start Self	1,923	1,933	1,852	1,831	1,994
	14.56%	14.46%	14.26%	13.93%	14.16%
Fresh Start Prosocial	1,866	1,975	1,815	1,844	2,005
	14.13%	14.78%	13.97%	14.03%	14.24%
Holdout	1,872	1,856	1,910	1,913	2,031
	14.18%	13.88%	14.70%	14.55%	14.42%

Legend: This table presents the number and percentage of patients who were assigned to a specific condition in the second RCT, among those who were in a given condition in the first RCT and were also in the second RCT's analysis sample. For example, among patients who were in the *Basic Reminder* condition of the first RCT and were included in the analysis sample of the second RCT, 1,880 (or 14.24%) were assigned to the *Basic Self* condition in the second RCT. Overall, this table shows that patients in any condition of the first RCT, if included in the analysis sample of the second RCT, had a virtually equal rate (approximately one seventh) to be assigned to one of the seven conditions in the second RCT. This confirms that group assignment in the first RCT was independent of that in the second RCT.

1.2 Additional Information About Outcome Measures and Control Variables

In the paper, we focus on two pre-registered outcome variables. *Appointment at UCLA (Within Six Days)* is a binary variable indicating whether patients scheduled a vaccination appointment for the first dose of COVID-19 vaccine at UCLA Health within six days of the first (second) reminder date (more precisely, from 3pm PST on the first (second) reminder date to the end of the fifth day later). *Vaccinated at UCLA (Within Four Weeks)* is a binary variable indicating whether patients received the first dose of COVID-19 vaccine at UCLA Health within one month (or more precisely, four full weeks) of the first (or second) reminder date. In addition to these outcome measures, we also pre-registered to eventually examine whether patients received the first dose of COVID-19 vaccine at UCLA Health or another location reporting to California Immunization Registry (CAIR) within two months (or more precisely, eight full weeks) of the first (or second) reminder date (*Vaccinated Anywhere (Within Eight Weeks)*).

In the paper, we focus on the two outcome variables tracking appointments and vaccinations at UCLA Health for two reasons. First, our text messages reminded patients of their vaccination eligibility at UCLA Health, encouraged them to schedule an appointment at UCLA Health, and provided a direct link to do so. Since our text messages facilitated patients with making appointments at UCLA Health, we expect that the effects of our interventions concentrate on appointments and vaccinations at UCLA Health. Second, our intention for reporting analyses based on patients enrolled in our RCTs by February 23, 2021 was to inform policymakers as soon as possible of the insights gleaned from our RCTs, so we did not plan to wait for several months to collect data about vaccinations obtained anywhere. We planned to use *Vaccinated Anywhere (Within Eight Weeks)* after data collection for both RCTs has been completed, so as to evaluate the comprehensive effects of our interventions on vaccinations across venues in a longer term. Nevertheless, to understand whether our effects observed at UCLA Health hold when we account for vaccinations obtained in other locations, we conducted a robustness check based on *Vaccinated Anywhere (Within Four Weeks)*, which indicates whether patients received the first dose anywhere within four weeks of the first (second) reminder date. This four-week time window exactly matches that used for *Vaccinated at UCLA (Within Four Weeks)*, so we can compare the results between these two ourcome measures. See Supplementary Tables 22 and 23 for this robustness check.

In addition to tracking *whether* patients received the COVID-19 vaccine, we also pre-registered two variables tracking *when* they got vaccinated: *Vaccination Time Lag at UCLA*—the number of days between a patient's first (or second) reminder date and the date when she received the first dose of COVID-19 vaccine at UCLA Health—and *Vaccination Time Lag Anywhere*—the number of days between a patient's first (or second) reminder date and the date when she received the first dose of COVID-19 vaccine anywhere. Since we intended to use these variables to capture how quickly patients got vaccinated, we did not plan to run specific regressions for these variables, but rather planned to plot the Kaplan Meier curve to capture how soon people got vaccinated.

Our regressions include a host of control variables, including an indicator for whether a patient was female ("Gender-Female"), an indicator for whether a patient's gender was either other or unknown ("Gender-Other"), patient age in years ("Age"), indicators for whether a patient was Hispanic/Latino ("Hispanic"), Black ("Black"), or Asian ("Asian"), an indicator for whether a patient's race was either American Indian/Alaska Native, Native Hawaiian/Pacific Islander, or other race ("Race-Other"), an indicator for whether a patient's race was unknown ("Race-Unknown"), an indicator for whether a patient's preferred language

was Spanish (“Preferring Spanish”), patient social vulnerability index (“Social Vulnerability Index (SVI)”), an indicator for whether a patient had a missing value for social vulnerability index (“Missing SVI”), patient COVID-19 risk score (“COVID-19 Risk Score”), an indicator for whether a patient had a missing value for COVID-19 risk score (“Missing Risk Score”), and batch fixed effects (indicators for the batch in which the patient received the initial invitation). “Black”, “Asian”, and “Race-Other” were coded as 0 if a patient was Hispanic or Latino.

For 19 patients with missing age information, we filled in the average age of patients in the first RCT sample. Regarding Social Vulnerability Index (SVI), to support health disparity reduction, UCLA Health incorporated the Centers for Disease Control and Prevention (CDC) SVI specific to the state of California to assign points for social risk. SVI is available at the census tract level and is applied to patients in the prioritization model. SVI incorporates the following features of each census tract: Socioeconomic status (e.g. poverty, income, education levels), household composition and disability (e.g. age distribution, single-parent households), minority status and language, and housing type and transportation (e.g. multi-unit structures, crowding, transportation access). Regarding COVID-19 risk score, UCLA used the CDC high risk conditions in the Covid-19 prioritization model. UCLA Health developed a clinical data infrastructure to identify conditions that are at increased risk or may be at increased risk for severe illness from COVID-19, based on the patients' problem list, administrative data (such as billing, encounter, admitting, discharge diagnoses, and health plan claims), lab data, and visit data. The clinical risk was then combined with social risk. The output of this COVID-19 Risk Model was the COVID-19 risk score we used as a covariate. For patients who did not have a value for SVI or COVID-19 risk score, we filled in the average value in the first RCT sample for each corresponding variable. Since a non-trivial number of patients had missing values (e.g., 6,852 patients missed SVI and 20,181 patients missed COVID-19 risk score in the first RCT sample), we also controlled for whether patients had missing values.

All of the control variables were pre-registered except for “Missing SVI” and “Missing Risk Score” because we only learned at the analysis stage that there were a non-trivial number of missing values for SVI and COVD-19 risk score. Note that all of our results about the two RCTs remain qualitatively the same if we do not include any controls (see Supplementary Tables 18-19).

1.3 Analyses and Results About the First RCT

1.3.1 Pre-registered Scope of Analyses About Patients Enrolled by February 23, 2021

As pre-registered (see the Tabular View tab at https://clinicaltrials.gov/ct2/show/record/NCT04800965), we sought to answer the following questions about patients enrolled in the first RCT by February 23, 2021.

1. What is the average effect of our follow-through text reminders?
2. Do all message types—the simplest message (i.e., Basic Reminder), the two messages containing ownership language, and the two messages containing a video—all outperform the *Holdout* arm?
3. What is the effect of including (vs. not including) a brief video-based information intervention in the text reminder?
4. What is the effect of adding (vs. not adding) ownership language to the text reminder?

5. Do the aforementioned effects vary based on whether or not patients received the flu vaccination in either the 2019-2020 season or the 2020-2021 season?

Due to uncertainty about how many people would be qualified for the first RCT's inclusion/exclusion criteria by February 23, 2021, we were concerned about not having enough power to test the interaction between the video intervention and the ownership intervention. Thus, we pre-registered to only test the interaction once data collection has been completed.

In addition to reporting regression analyses, we also pre-registered that we would show the raw data for each condition without conducting hypothesis testing. The main text of the paper contains a figure that displays the average appointment and vaccination rates at UCLA Health in each of the five conditions (four sub-arms within the *Follow-Through Reminder* arm plus the *Holdout* arm).

We employed a two-stage gatekeeping design[50] to control the familywise type I error rate for our primary analyses at the 5% level. Specifically, we planned to first compare the combined *Follow-through Reminder* arm with the *Holdout* arm for each outcome variable and, if this comparison leads to statistically significant differences, we would next evaluate the difference between each type of message (Basic Reminder, texts containing the ownership language, texts containing the video) and the *Holdout* arm to explore whether every type of message consistently outperforms the *Holdout* arm. When presenting results about the added value of ownership language and video-based information intervention, we report the p-value of an effect both with and without a Holm-Bonferroni correction for multiple comparisons[37] if it is significant at the 5% without adjustment. We take this approach because this analysis involves testing two interventions (ownership framing and video) at the same stage and is parallel to the analysis testing the average effect of receiving a text reminder. We regard all other analyses reported below across demographic groups and across patients with or without recent flu vaccinations as exploratory (or as robustness checks), and so do not perform further adjustments.

1.3.2 The Average Effect of Receiving a Text Reminder

To estimate the average effect of receiving a follow-through text reminder, we used the following ordinary least squares (OLS) regression specification:

$$Y_i = \beta_0 + \beta_1 \textit{Follow-Through Reminder}_i + \beta_2 X_i + \epsilon_i, \quad (1)$$

where Y_i represents the outcome variable of patient i, and $\textit{Follow-Through Reminder}_i$ equals 1 if patient i was assigned to the *Follow-Through Reminder* arm and 0 if patient i was assigned to the *Holdout* arm. X_i represents a host of control variables listed in Supplementary Information Section 1.2. We report robust standard errors. We report OLS regression results in Supplementary Tables 3.

To interpret why the increase in vaccination rates at UCLA Health within four weeks after the first reminder date (i.e., 3.57 percentage points) was smaller than that in appointment rates at UCLA Health within six days (6.07 percentage points), we created the Kaplan Meier curves to reflect the percentage of patients who had scheduled the first-dose appointment at UCLA Health by a given day during *four weeks* after the first reminder date (Supplementary Figure 1). We have two observations. First, though the gap between the *Holdout* arm and *Follow-through Reminder* arm reduced over four weeks, the gap did not disappear. The drop of the gap from about 6 percentage points in the first week to about 4 percentage

points in four weeks suggests that the observed effect of our text reminders on appointments within six days of the first reminder date is a combination of appointment acceleration and conversion. The 4-percentage-point gap at the end of four weeks observed in Supplementary Figure 1 is consistent with our regression that predicts whether patients made the first-dose appointment at UCLA Health within four weeks after the first reminder (Supplementary Table 22): The regression suggests that appointment rates within four weeks were significantly higher in the *Follow-Through Reminder* arm than the *Holdout* arm by 3.72 percentage points (B=0.037, SE = 0.003, p<0.001).

Our second observation is that the gap exhibited the biggest drop around eight days after the first reminder date (marked by the dashed vertical horizontal line in Supplementary Figure 1). That corresponds to the date when the second text reminder was sent to patients who had not yet scheduled an appointment or obtained the vaccine. Since we re-randomized participants in the second RCT, some patients in the *Holdout* arm of the first RCT received the second text reminder, which may have helped them catch up to the *Follow-Through Reminder* arm of the first RCT.

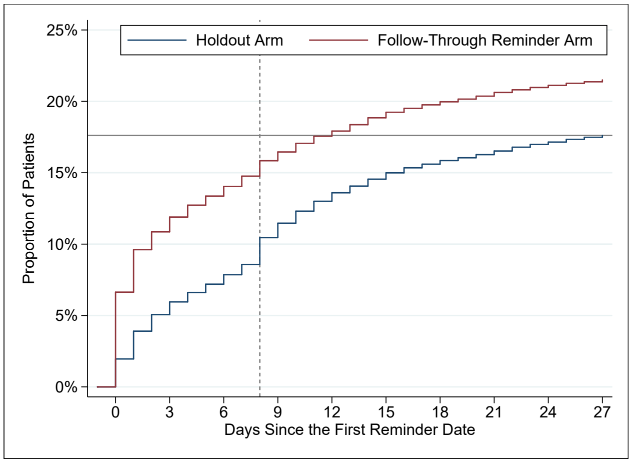

Figure 1: Kaplan-Meier Curves Reflecting the Proportion of Patients Who Had Scheduled the First Dose at UCLA Health by a Given Day After the First Reminder Date

Legend: This figure depicts the Kaplan-Meier curves tracking the percentage of patients in the *Holdout* arm (blue; N = 18,749) versus the *Follow-Through Reminder* arm (red; N = 74,605) of the first RCT who had scheduled an appointment for the first dose of COVID-19 vaccine at UCLA Health by a given day from the first reminder date (0 on the x-axis) to 27 days later. The solid horizontal line represents the proportion of patients who had scheduled an appointment by the end of 27 days after the first reminder date (i.e., 17.6%). The dashed vertical line indicates the eighth day following the first reminder date.

Table 3: Effects of Receiving a Follow-Through Text Reminder on Appointments and Vaccinations at UCLA Health

Dependent Measure	Appointment at UCLA (Within Six Days) (1)	Vaccinated at UCLA (Within Four Weeks) (2)
Follow-Through Reminder	0.061***	0.036***
	(0.002)	(0.003)
Gender-Female	-0.005*	-0.010***
	(0.002)	(0.002)
Gender-Other	-0.015	-0.035
	(0.030)	(0.030)
Age	-0.002***	-0.003***
	(0.000)	(0.000)
Hispanic	0.012**	0.027***
	(0.004)	(0.005)
Black	0.038***	0.066***
	(0.005)	(0.006)
Asian	0.015***	0.019***
	(0.004)	(0.005)
Race-Other	-0.016***	-0.014**
	(0.004)	(0.005)
Race-Unknown	-0.038***	-0.048***
	(0.003)	(0.003)
Preferring Spanish	0.009	0.025***
	(0.006)	(0.007)
Social Vulnerability Index (SVI)	-0.001***	-0.001***
	(0.000)	(0.000)
Missing SVI	-0.063***	-0.096***
	(0.003)	(0.003)
COVID-19 Risk Score	-0.000	-0.000
	(0.000)	(0.000)
Missing Risk Score	-0.054***	-0.071***
	(0.003)	(0.004)
N	93354	93354
R^2	0.045	0.051

Legend: The table reports ordinary least squares (OLS) regressions predicting whether patients scheduled an appointment to get the first dose of the COVID-19 vaccine at UCLA Health within six days of the first reminder date (Column 1) and whether patients obtained the first dose of the COVID-19 vaccine at UCLA Health within four weeks of the first reminder date (Column 2). The primary predictor, *Follow-Through Reminder*, is a dummy variable coded as 1 when participants received one of the text reminders in the first RCT. Its p-value is < 0.001 in both columns. Control variables include an indicator for whether a patient was female ("Gender-Female"), an indicator for whether a patient's gender was either other or unknown ("Gender-Other"), patient age in years ("Age"), indicators for whether a patient was Hispanic/Latino ("Hispanic"), Black ("Black"), or Asian ("Asian"), an indicator for whether a patient's race was either American Indian/Alaska Native, Native Hawaiian/Pacific Islander, or other race ("Race-Other"), an indicator for whether a patient's race was unknown ("Race-Unknown"), an indicator for whether a patient's preferred language was Spanish ("Preferring Spanish"), patient social vulnerability index ("Social Vulnerability Index (SVI)"), an indicator for whether a patient had a missing value for social vulnerability index ("Missing SVI"), patient COVID-19 risk score ("COVID-19 Risk Score"), an indicator for whether a patient had a missing value for COVID-19 risk score ("Missing Risk Score"), and batch fixed effects. "Black", "Asian", and "Race-Other" were coded as 0 if a patient was Hispanic or Latino. Robust standard errors are reported in parentheses. *p<0.05; **p<0.01; ***p<0.001 (all two-sided).

1.3.3 Comparing Different Types of Text Reminders with Holdout

Next, we break down the conditions within the *Follow-Through Reminder* arm and test whether the *Basic Reminder* condition, the two conditions containing ownership language, and the two conditions containing a video all outperformed the *Holdout* arm. To answer this question, we conducted three sets of regressions:

$$Y_i = \beta_0 + \beta_1 Basic\ Reminder_i + \beta_2 X_i + \epsilon_i, \quad (2)$$

Specification (2) only includes patients in the *Holdout* arm and the *Basic Reminder* condition. Y_i represents the outcome variable of patient i. $Basic\ Reminder_i$ equals 1 if patient i was assigned to the *Basic Reminder* condition and 0 otherwise. X_i represents a host of control variables listed in Supplementary Information Section 1.2. We report robust standard errors.

$$Y_i = \beta_0 + \beta_1 Ownership_i + \beta_2 X_i + \epsilon_i, \quad (3)$$

Specification (3) only includes patients in the *Holdout* arm, *Ownership Reminder* condition, and *Ownership Reminder with Video* condition. Y_i represents the outcome variable of patient i. $Ownership_i$ equals 1 if patient i was assigned to the latter two conditions that contained ownership language and 0 otherwise. X_i represents a host of control variables listed in Supplementary Information Section 1.2. We report robust standard errors.

$$Y_i = \beta_0 + \beta_1 Video_i + \beta_2 X_i + \epsilon_i, \quad (4)$$

Specification (4) only includes patients in the *Holdout* arm, *Basic Reminder with Video* condition, and *Ownership Reminder with Video* condition. Y_i represents the outcome variable of patient i. $Video_i$ equals 1 if patient i was assigned to the latter two conditions that contained the video and 0 otherwise. X_i represents a host of control variables listed in Supplementary Information Section 1.2. We report robust standard errors.

Supplementary Table 4 reports the results from specifications (2)-(4).

Table 4: Effects of Different Types of Reminders on Appointments and Vaccinations at UCLA Health

Dependent Measure	Appointment at UCLA (Within Six Days)			Vaccinated at UCLA (Within Four Weeks)		
	(1)	(2)	(3)	(4)	(5)	(6)
Basic Reminder	0.055***			0.031***		
	(0.003)			(0.004)		
Ownership		0.068***			0.041***	
		(0.003)			(0.003)	
Video			0.059***			0.035***
			(0.003)			(0.003)
Gender-Female	-0.001	-0.005*	-0.005*	-0.007*	-0.011***	-0.011***
	(0.003)	(0.003)	(0.003)	(0.004)	(0.003)	(0.003)
Gender-Other	-0.011	-0.033	0.007	-0.027	-0.058**	-0.013
	(0.014)	(0.017)	(0.046)	(0.015)	(0.018)	(0.044)
Age	-0.002***	-0.002***	-0.002***	-0.003***	-0.003***	-0.003***
	(0.000)	(0.000)	(0.000)	(0.000)	(0.000)	(0.000)
Hispanic	0.010	0.015**	0.010	0.029***	0.023***	0.028***
	(0.006)	(0.005)	(0.005)	(0.008)	(0.006)	(0.006)
Black	0.024**	0.044***	0.035***	0.057***	0.068***	0.069***
	(0.008)	(0.007)	(0.007)	(0.009)	(0.008)	(0.008)
Asian	0.016**	0.016**	0.020***	0.023**	0.014*	0.020***
	(0.006)	(0.005)	(0.005)	(0.007)	(0.006)	(0.006)
Race-Other	-0.011	-0.019***	-0.013*	-0.011	-0.011	-0.001
	(0.006)	(0.005)	(0.005)	(0.007)	(0.006)	(0.006)
Race-Unknown	-0.036***	-0.036***	-0.038***	-0.044***	-0.046***	-0.045***
	(0.004)	(0.003)	(0.003)	(0.005)	(0.004)	(0.004)
Preferring Spanish	0.024**	0.007	0.007	0.035**	0.028**	0.017
	(0.009)	(0.007)	(0.007)	(0.011)	(0.009)	(0.009)
Social Vulnerability Index (SVI)	-0.001***	-0.001***	-0.001***	-0.001***	-0.001***	-0.001***
	(0.000)	(0.000)	(0.000)	(0.000)	(0.000)	(0.000)
Missing SVI	-0.051***	-0.065***	-0.054***	-0.090***	-0.098***	-0.089***
	(0.004)	(0.004)	(0.004)	(0.005)	(0.004)	(0.004)
COVID-19 Risk Score	-0.000	-0.000	-0.000*	-0.000	-0.000	-0.000
	(0.000)	(0.000)	(0.000)	(0.000)	(0.000)	(0.000)
Missing Risk Score	-0.047***	-0.053***	-0.053***	-0.068***	-0.069***	-0.070***
	(0.005)	(0.004)	(0.004)	(0.006)	(0.005)	(0.005)
N	37378	55968	56133	37378	55968	56133
R^2	0.045	0.050	0.046	0.052	0.053	0.050
Conditions Included	Basic Reminder	Ownership Reminder<br>Ownership Reminder w/ Video	Basic Reminder w/ Video<br>Ownership Reminder w/ Video	Basic Reminder	Ownership Reminder<br>Ownership Reminder w/ Video	Basic Reminder w/ Video<br>Ownership Reminder w/ Video
	Holdout	Holdout	Holdout	Holdout	Holdout	Holdout

Legend: The table reports ordinary least squares (OLS) regressions predicting whether patients scheduled an appointment to get the first dose of the COVID-19 vaccine at UCLA Health within six days of the first reminder date (Columns 1-3) and whether patients obtained the first dose of the COVID-19 vaccine at UCLA Health within four weeks of the first reminder date (Columns 4-6). *Basic Reminder* is a dummy variable coded as 1 when participants received the *Basic Reminder* in the first RCT. Its p-value is < 0.001 in Columns 1 and 4. *Ownership* is a dummy variable coded as 1 when participants received one of the messages containing the ownership language in the first RCT. Its p-value is < 0.001 in Columns 2 and 5. *Video* is a dummy variable coded as 1 when participants received one of the messages containing a link to the video in the first RCT. Its p-value is < 0.001 in Columns 3 and 6. Control variables include an indicator for whether a patient was female ("Gender-Female"), an indicator for whether a patient's gender was either other or unknown ("Gender-Other"), patient age in years ("Age"), indicators for whether a patient was Hispanic/Latino ("Hispanic"), Black ("Black"), or Asian ("Asian"), an indicator for whether a patient's race was either American Indian/Alaska Native, Native Hawaiian/Pacific Islander, or other race ("Race-Other"), an indicator for whether a patient's race was unknown ("Race-Unknown"), an indicator for whether a patient's preferred language was Spanish ("Preferring Spanish"), patient social vulnerability index ("Social Vulnerability Index (SVI)"), an indicator for whether a patient had a missing value for social vulnerability index ("Missing SVI"), patient COVID-19 risk score ("COVID-19 Risk Score"), an indicator for whether a patient had a missing value for COVID-19 risk score ("Missing Risk Score"), and batch fixed effects. "Black", "Asian", and "Race-Other" were coded as 0 if a patient was Hispanic or Latino. Robust standard errors are reported in parentheses. $^{*}p<0.05$; $^{**}p<0.01$; $^{***}p<0.001$ (all two-sided).

1.3.4 The Additive Effects of Ownership Language and Video-based Information Intervention

Next, we estimate the effects of (1) adding language designed to induce feelings of psychological ownership and (2) adding a link to the video designed to shift peoples' beliefs and perceptions about COVID-19 and the vaccine. We used the following OLS regression specification, which only involves people in the *Follow-Through Reminder* arm, given our focus on the additive effects of ownership language and video.

$$Y_i = \beta_0 + \beta_1 \mathit{Ownership}_i + \beta_2 \mathit{Video}_i + \beta_3 X_i + \epsilon_i, \quad (5)$$

where Y_i represents the outcome variable of patient i; $Ownership_i$ and $Video_i$ are defined as in equations (3) and (4). X_i represents a host of control variables listed in Supplementary Information Section 1.2 We report robust standard errors. Supplementary Table 5 reports the results from specification (5).

We anonymously tracked the total number of clicks on the link to the video, the total number of video views, their watch time, and the total number of patients who finished watching the video each day. We focused on patients whose first reminder date was between February 1 and February 17, 2021, because no text reminders for the first RCT were sent during February 18-21, 2021. We could attribute all link clicks and video views that happened by the end of February 21, 2021, to patients who had been invited to watch the video by February 17, 2021, and those patients were unlikely to go back to the text message and watch the video after February 21, 2021, since it was five or more days after their first reminder date. Using this approach, we count that the average number of link clicks per patient was 0.28 per patient, the average number of video views per patient was 0.21, and the average number of finishes per patient was 0.14. This means that at most 28% of patients clicked the link to the video, 21% watched the video, and 14% finished watching it. Conditional on starting to watch the video, patients watched about 77% of the video.

Table 5: The Additive Effects of Ownership Language and Video on Appointments and Vaccinations at UCLA Health

Dependent Measure	Appointment at UCLA (Within Six Days) (1)	Vaccinated at UCLA (Within Four Weeks) (2)
Ownership	0.015***	0.011***
	(0.002)	(0.003)
Video	-0.003	-0.002
	(0.002)	(0.003)
Gender-Female	-0.007**	-0.011***
	(0.002)	(0.003)
Gender-Other	-0.024	-0.042
	(0.041)	(0.040)
Age	-0.003***	-0.003***
	(0.000)	(0.000)
Hispanic	0.012*	0.026***
	(0.005)	(0.006)
Black	0.043***	0.068***
	(0.006)	(0.007)
Asian	0.013**	0.019***
	(0.005)	(0.005)
Race-Other	-0.016***	-0.017***
	(0.005)	(0.005)
Race-Unknown	-0.039***	-0.050***
	(0.003)	(0.003)
Preferring Spanish	0.008	0.026***
	(0.007)	(0.008)
Social Vulnerability Index (SVI)	-0.001***	-0.001***
	(0.000)	(0.000)
Missing SVI	-0.070***	-0.100***
	(0.003)	(0.004)
COVID-19 Risk Score	-0.000	0.000
	(0.000)	(0.000)
Missing Risk Score	-0.058***	-0.074***
	(0.004)	(0.004)
N	74605	74605
R^2	0.042	0.051
Conditions Included	Conditions within the Follow-Through Reminder arm	

Legend: The table reports ordinary least squares (OLS) regressions predicting whether patients scheduled an appointment to get the first dose of the COVID-19 vaccine at UCLA Health within six days of the first reminder date (Column 1) and whether patients obtained the first dose of the COVID-19 vaccine at UCLA Health within four weeks of the first reminder date (Column 2). *Ownership* is a dummy variable coded as 1 when participants received one of the messages containing the ownership language in the first RCT. Its p-value is < 0.001 in both columns without multiple comparison adjustment and becomes < 0.002 with a Holm-Bonferroni correction. *Video* is a dummy variable coded as 1 when participants received one of the messages containing a link to the video in the first RCT. Its p-value is 0.258 in Column 1 and 0.500 in Column 2. Control variables include an indicator for whether a patient was female ("Gender-Female"), an indicator for whether a patient's gender was either other or unknown ("Gender-Other"), patient age in years ("Age"), indicators for whether a patient was Hispanic/Latino ("Hispanic"), Black ("Black"), or Asian ("Asian"), an indicator for whether a patient's race was either American Indian/Alaska Native, Native Hawaiian/Pacific Islander, or other race ("Race-Other"), an indicator for whether a patient's race was unknown ("Race-Unknown"), an indicator for whether a patient's preferred language was Spanish ("Preferring Spanish"), patient social vulnerability index ("Social Vulnerability Index (SVI)"), an indicator for whether a patient had a missing value for social vulnerability index ("Missing SVI"), patient COVID-19 risk score ("COVID-19 Risk Score"), an indicator for whether a patient had a missing value for COVID-19 risk score ("Missing Risk Score"), and batch fixed effects. "Black", "Asian", and "Race-Other" were coded as 0 if a patient was Hispanic or Latino. Robust standard errors are reported in parentheses. *p<0.05; **p<0.01; ***p<0.001 (all two-sided).

1.3.5 The Heterogeneous Treatment Effect by Flu Vaccination Status

As pre-registered, we explored how the effects of our interventions varied depending on whether patients got the flu shot in either the 2019-2020 season (from August 1, 2019 to March 31, 2020) or the 2020-2021 season (from August 1, 2020 to March 31, 2020) based on patients' medical record at UCLA Health. We used flu vaccination status as a proxy for people's baseline vaccination intentions, as some survey evidence suggests that individuals' intentions to get vaccinated against COVID-19 are higher among people with previous flu vaccination behavior[32,51]. We separately applied regression specifications (1)-(5) to the two subgroups of patients (with or without getting the flu vaccine), and we also interacted our predictor(s) in each specification with a dummy variable (*Flu Shot*) that equals 1 for patients who got the flu shot in either of the two seasons and 0 otherwise.

All results are presented in Supplementary Tables 6-10.

Table 6: Effects of Receiving a Text Reminder, Broken Down by Prior Flu Vaccination Status

Dependent Measure	Appointment at UCLA (Within Six Days)			Vaccinated at UCLA (Within Four Weeks)		
Sample	Flu Shot=0	Flu Shot=1	Both	Flu Shot=0	Flu Shot=1	Both
	(1)	(2)	(3)	(4)	(5)	(6)
Follow-Through Reminder	0.039***	0.083***	0.039***	0.025***	0.047***	0.025***
	(0.002)	(0.004)	(0.002)	(0.003)	(0.005)	(0.003)
Flu Shot			0.053***			0.099***
			(0.004)			(0.005)
Follow-Through Reminder x Flu Shot			0.044***			0.023***
			(0.004)			(0.006)
Gender-Female	-0.002	-0.004	-0.004	-0.008**	-0.007	-0.008***
	(0.002)	(0.003)	(0.002)	(0.003)	(0.004)	(0.002)
Gender-Other	-0.031***	0.240	0.012	-0.047***	0.199	0.001
	(0.007)	(0.267)	(0.027)	(0.008)	(0.262)	(0.026)
Age	-0.002***	-0.002***	-0.002***	-0.002***	-0.003***	-0.003***
	(0.000)	(0.000)	(0.000)	(0.000)	(0.000)	(0.000)
Hispanic	0.007	0.010	0.008*	0.013*	0.028***	0.022***
	(0.005)	(0.007)	(0.004)	(0.006)	(0.007)	(0.005)
Black	0.019**	0.062***	0.040***	0.038***	0.102***	0.069***
	(0.006)	(0.009)	(0.005)	(0.007)	(0.010)	(0.006)
Asian	0.006	0.017**	0.013**	0.006	0.024**	0.016***
	(0.005)	(0.007)	(0.004)	(0.005)	(0.007)	(0.005)
Race-Other	-0.007	-0.016*	-0.011**	-0.007	-0.006	-0.006
	(0.004)	(0.007)	(0.004)	(0.005)	(0.008)	(0.005)
Race-Unknown	-0.020***	-0.050***	-0.032***	-0.027***	-0.060***	-0.041***
	(0.003)	(0.005)	(0.003)	(0.003)	(0.006)	(0.003)
Preferring Spanish	-0.011	0.022*	0.006	-0.006	0.043***	0.021**
	(0.006)	(0.009)	(0.006)	(0.008)	(0.011)	(0.007)
Social Vulnerability Index (SVI)	-0.001***	-0.001***	-0.001***	-0.001***	-0.001***	-0.001***
	(0.000)	(0.000)	(0.000)	(0.000)	(0.000)	(0.000)
Missing SVI	-0.043***	-0.072***	-0.053***	-0.066***	-0.114***	-0.082***
	(0.003)	(0.006)	(0.003)	(0.003)	(0.007)	(0.003)
COVID-19 Risk Score	-0.000**	0.000	-0.000	-0.000	0.000*	-0.000
	(0.000)	(0.000)	(0.000)	(0.000)	(0.000)	(0.000)
Missing Risk Score	-0.029***	-0.060***	-0.040***	-0.042***	-0.076***	-0.052***
	(0.004)	(0.007)	(0.003)	(0.004)	(0.008)	(0.004)
N	46597	46757	93354	46597	46757	93354
R^2	0.032	0.044	0.063	0.036	0.046	0.074

Legend: The table reports ordinary least squares (OLS) regressions predicting whether patients scheduled an appointment to get the first dose of the COVID-19 vaccine at UCLA Health within six days of the first reminder date (Columns 1-3) and whether patients obtained the first dose of the COVID-19 vaccine at UCLA Health within four weeks of the first reminder date (Columns 4-6), broken down by whether patients got the flu shot in either the 2019-2020 or the 2020-2021 flu season. Columns 1 and 4 focus on patients who got the flu shot, Columns 2 and 5 focus on patients who did not get the flu shot, and Columns 3 and 6 focus on the combined sample. *Follow-Through Reminder* is a dummy variable coded as 1 when participants received one of the text reminders in the first RCT. Its p-value is < 0.001 in all columns. Its interaction with Flu Shot has a p-value of < 0.001 in Columns 3 and 6. Control variables include an indicator for whether a patient was female (“Gender-Female”), an indicator for whether a patient’s gender was either other or unknown (“Gender-Other”), patient age in years (“Age”), indicators for whether a patient was Hispanic/Latino (“Hispanic”), Black (“Black”), or Asian (“Asian”), an indicator for whether a patient’s race was either American Indian/Alaska Native, Native Hawaiian/Pacific Islander, or other race (“Race-Other”), an indicator for whether a patient’s race was unknown (“Race-Unknown”), an indicator for whether a patient’s preferred language was Spanish (“Preferring Spanish”), patient social vulnerability index (“Social Vulnerability Index (SVI)”), an indicator for whether a patient had a missing value for social vulnerability index (“Missing SVI”), patient COVID-19 risk score (“COVID-19 Risk Score”), an indicator for whether a patient had a missing value for COVID-19 risk score (“Missing Risk Score”), and batch fixed effects. “Black”, “Asian”, and “Race-Other” were coded as 0 if a patient was Hispanic or Latino. Robust standard errors are reported in parentheses. *p<0.05; **p<0.01; ***p<0.001 (all two-sided).

Table 7: Effects of Basic Reminder (vs. Holdout), Broken Down by Prior Flu Vaccination Status

Dependent Measure	Appointment at UCLA (Within Six Days)			Vaccinated at UCLA (Within Four Weeks)		
Sample	Flu Shot=0	Flu Shot=1	Both	Flu Shot=0	Flu Shot=1	Both
	(1)	(2)	(3)	(4)	(5)	(6)
Basic Reminder	0.035***	0.077***	0.035***	0.023***	0.042***	0.022***
	(0.003)	(0.005)	(0.003)	(0.004)	(0.006)	(0.004)
Flu Shot			0.056***			0.099***
			(0.004)			(0.005)
Basic Reminder x Flu Shot			0.041***			0.020**
			(0.006)			(0.007)
Gender-Female	0.005	-0.004	0.000	-0.002	-0.007	-0.005
	(0.003)	(0.005)	(0.003)	(0.004)	(0.006)	(0.004)
Gender-Other	-0.017		0.012	-0.026*		0.009
	(0.011)		(0.011)	(0.013)		(0.013)
Age	-0.001***	-0.002***	-0.001***	-0.003***	-0.002***	-0.002***
	(0.000)	(0.000)	(0.000)	(0.000)	(0.000)	(0.000)
Hispanic	-0.002	0.012	0.007	0.015	0.032**	0.025***
	(0.007)	(0.010)	(0.006)	(0.009)	(0.012)	(0.008)
Black	0.013	0.041**	0.026***	0.027*	0.096***	0.060***
	(0.008)	(0.013)	(0.008)	(0.010)	(0.015)	(0.009)
Asian	0.015*	0.012	0.014*	0.019*	0.021	0.021**
	(0.007)	(0.010)	(0.006)	(0.009)	(0.011)	(0.007)
Race-Other	0.001	-0.015	-0.006	0.002	-0.012	-0.005
	(0.006)	(0.010)	(0.006)	(0.008)	(0.012)	(0.007)
Race-Unknown	-0.019***	-0.048***	-0.031***	-0.029***	-0.049***	-0.037***
	(0.004)	(0.007)	(0.004)	(0.005)	(0.009)	(0.005)
Preferring Spanish	0.001	0.043**	0.023**	0.014	0.049**	0.032**
	(0.009)	(0.014)	(0.009)	(0.012)	(0.017)	(0.011)
Social Vulnerability Index (SVI)	-0.001***	-0.001***	-0.001***	-0.001***	-0.001***	-0.001***
	(0.000)	(0.000)	(0.000)	(0.000)	(0.000)	(0.000)
Missing SVI	-0.030***	-0.066***	-0.042***	-0.060***	-0.113***	-0.077***
	(0.004)	(0.009)	(0.004)	(0.004)	(0.010)	(0.005)
COVID-19 Risk Score	-0.000	0.000	-0.000	-0.000	0.000	-0.000
	(0.000)	(0.000)	(0.000)	(0.000)	(0.000)	(0.000)
Missing Risk Score	-0.027***	-0.052***	-0.035***	-0.042***	-0.074***	-0.050***
	(0.005)	(0.010)	(0.005)	(0.006)	(0.012)	(0.006)
N	18679	18699	37378	18679	18699	37378
R^2	0.034	0.045	0.061	0.040	0.044	0.073
Conditions Included	Basic Reminder and Holdout					

Legend: The table reports ordinary least squares (OLS) regressions predicting whether patients scheduled an appointment to get the first dose of the COVID-19 vaccine at UCLA Health within six days of the first reminder date (Columns 1-3) and whether patients obtained the first dose of the COVID-19 vaccine at UCLA Health within four weeks of the first reminder date (Columns 4-6), broken down by whether patients got the flu shot in either the 2019-2020 or the 2020-2021 flu season. Columns 1 and 4 focus on patients who got the flu shot, Columns 2 and 5 focus on patients who did not get the flu shot, and Columns 3 and 6 focus on the combined sample. *Basic Reminder* is a dummy variable coded as 1 when participants received the *Basic Reminder* message in the first RCT. Its p-value is < 0.001 in all columns. Its interaction with Flu Shot has a p-value of < 0.001 in Column 3 and a p-value of 0.006 in Column 6. Control variables include an indicator for whether a patient was female (“Gender-Female”), an indicator for whether a patient’s gender was either other or unknown (“Gender-Other”), patient age in years (“Age”), indicators for whether a patient was Hispanic/Latino (“Hispanic”), Black (“Black”), or Asian (“Asian”), an indicator for whether a patient’s race was either American Indian/Alaska Native, Native Hawaiian/Pacific Islander, or other race (“Race-Other”), an indicator for whether a patient’s race was unknown (“Race-Unknown”), an indicator for whether a patient’s preferred language was Spanish (“Preferring Spanish”), patient social vulnerability index (“Social Vulnerability Index (SVI)”), an indicator for whether a patient had a missing value for social vulnerability index (“Missing SVI”), patient COVID-19 risk score (“COVID-19 Risk Score”), an indicator for whether a patient had a missing value for COVID-19 risk score (“Missing Risk Score”), and batch fixed effects. “Black”, “Asian”, and “Race-Other” were coded as 0 if a patient was Hispanic or Latino. Robust standard errors are reported in parentheses. $^{*}p<0.05$; $^{**}p<0.01$; $^{***}p<0.001$ (all two-sided).

Table 8: Effects of Ownership Messages (vs. Holdout), Broken Down by Prior Flu Vaccination Status

Dependent Measure	Appointment at UCLA (Within Six Days)			Vaccinated at UCLA (Within Four Weeks)		
Sample	Flu Shot=0	Flu Shot=1	Both	Flu Shot=0	Flu Shot=1	Both
	(1)	(2)	(3)	(4)	(5)	(6)
Ownership	0.045***	0.093***	0.044***	0.028***	0.056***	0.027***
	(0.003)	(0.004)	(0.003)	(0.003)	(0.005)	(0.003)
Flu Shot			0.054***			0.098***
			(0.004)			(0.005)
Ownership x Flu Shot			0.049***			0.028***
			(0.005)			(0.006)
Gender-Female	-0.004	-0.004	-0.004	-0.009**	-0.008	-0.009**
	(0.003)	(0.004)	(0.003)	(0.004)	(0.005)	(0.003)
Gender-Other	-0.023*	-0.147***	-0.010	-0.043***	-0.178***	-0.024
	(0.010)	(0.010)	(0.017)	(0.011)	(0.012)	(0.020)
Age	-0.002***	-0.002***	-0.002***	-0.002***	-0.003***	-0.003***
	(0.000)	(0.000)	(0.000)	(0.000)	(0.000)	(0.000)
Hispanic	0.005	0.015	0.011*	0.004	0.027**	0.018**
	(0.006)	(0.008)	(0.005)	(0.007)	(0.010)	(0.006)
Black	0.027***	0.064***	0.046***	0.038***	0.103***	0.070***
	(0.008)	(0.011)	(0.007)	(0.009)	(0.013)	(0.008)
Asian	0.007	0.018*	0.014**	0.002	0.018	0.011
	(0.006)	(0.008)	(0.005)	(0.007)	(0.009)	(0.006)
Race-Other	-0.008	-0.020*	-0.013**	-0.004	-0.005	-0.004
	(0.005)	(0.009)	(0.005)	(0.007)	(0.010)	(0.006)
Race-Unknown	-0.016***	-0.051***	-0.030***	-0.024***	-0.061***	-0.039***
	(0.004)	(0.006)	(0.003)	(0.004)	(0.007)	(0.004)
Preferring Spanish	-0.008	0.013	0.003	-0.007	0.048***	0.023**
	(0.008)	(0.012)	(0.007)	(0.009)	(0.014)	(0.009)
Social Vulnerability Index (SVI)	-0.000***	-0.001***	-0.001***	-0.001***	-0.001***	-0.001***
	(0.000)	(0.000)	(0.000)	(0.000)	(0.000)	(0.000)
Missing SVI	-0.044***	-0.077***	-0.055***	-0.066***	-0.119***	-0.084***
	(0.003)	(0.008)	(0.004)	(0.004)	(0.009)	(0.004)
COVID-19 Risk Score	-0.000*	0.000	-0.000	-0.000	0.000*	0.000
	(0.000)	(0.000)	(0.000)	(0.000)	(0.000)	(0.000)
Missing Risk Score	-0.031***	-0.055***	-0.039***	-0.042***	-0.071***	-0.050***
	(0.005)	(0.009)	(0.004)	(0.005)	(0.010)	(0.005)
N	27896	28072	55968	27896	28072	55968
R^2	0.037	0.051	0.068	0.039	0.048	0.077
Conditions Included	Ownership Reminder, Ownership Reminder w/ Video, and Holdout					

Legend: The table reports ordinary least squares (OLS) regressions predicting whether patients scheduled an appointment to get the first dose of the COVID-19 vaccine at UCLA Health within six days of the first reminder date (Columns 1-3) and whether patients obtained the first dose of the COVID-19 vaccine at UCLA Health within four weeks of the first reminder date (Columns 4-6), broken down by whether patients got the flu shot in either the 2019-2020 or the 2020-2021 flu season. Columns 1 and 4 focus on patients who got the flu shot, Columns 2 and 5 focus on patients who did not get the flu shot, and Columns 3 and 6 focus on the combined sample. *Ownership* is a dummy variable coded as 1 when participants received one of the messages containing the ownership language in the first RCT. Its p-value is < 0.001 in all columns. Its interaction with Flu Shot has a p-value of < 0.001 in Columns 3 and 6. Control variables include an indicator for whether a patient was female ("Gender-Female"), an indicator for whether a patient's gender was either other or unknown ("Gender-Other"), patient age in years ("Age"), indicators for whether a patient was Hispanic/Latino ("Hispanic"), Black ("Black"), or Asian ("Asian"), an indicator for whether a patient's race was either American Indian/Alaska Native, Native Hawaiian/Pacific Islander, or other race ("Race-Other"), an indicator for whether a patient's race was unknown ("Race-Unknown"), an indicator for whether a patient's preferred language was Spanish ("Preferring Spanish"), patient social vulnerability index ("Social Vulnerability Index (SVI)"), an indicator for whether a patient had a missing value for social vulnerability index ("Missing SVI"), patient COVID-19 risk score ("COVID-19 Risk Score"), an indicator for whether a patient had a missing value for COVID-19 risk score ("Missing Risk Score"), and batch fixed effects. "Black", "Asian", and "Race-Other" were coded as 0 if a patient was Hispanic or Latino. Robust standard errors are reported in parentheses. *p<0.05; **p<0.01; ***p<0.001 (all two-sided).

Table 9: Effects of Video Messages (vs. Holdout), Broken Down by Prior Flu Vaccination Status

Dependent Measure	Appointment at UCLA (Within Six Days)			Vaccinated at UCLA (Within Four Weeks)		
Sample	Flu Shot=0	Flu Shot=1	Both	Flu Shot=0	Flu Shot=1	Both
	(1)	(2)	(3)	(4)	(5)	(6)
Video	0.039***	0.080***	0.039***	0.026***	0.045***	0.025***
	(0.003)	(0.004)	(0.003)	(0.003)	(0.005)	(0.003)
Flu Shot			0.054***			0.100***
			(0.004)			(0.005)
Video x Flu Shot			0.041***			0.020**
			(0.005)			(0.006)
Gender-Female	-0.003	-0.005	-0.004	-0.010**	-0.007	-0.009**
	(0.003)	(0.004)	(0.003)	(0.004)	(0.005)	(0.003)
Gender-Other	-0.027**	0.256	0.027	-0.044***	0.215	0.016
	(0.009)	(0.268)	(0.042)	(0.010)	(0.262)	(0.039)
Age	-0.002***	-0.002***	-0.002***	-0.002***	-0.003***	-0.003***
	(0.000)	(0.000)	(0.000)	(0.000)	(0.000)	(0.000)
Hispanic	0.009	0.004	0.006	0.016*	0.028**	0.023***
	(0.006)	(0.008)	(0.005)	(0.007)	(0.010)	(0.006)
Black	0.017*	0.058***	0.037***	0.041***	0.105***	0.073***
	(0.007)	(0.011)	(0.007)	(0.009)	(0.013)	(0.008)
Asian	0.011	0.023**	0.018***	0.008	0.025**	0.018**
	(0.006)	(0.008)	(0.005)	(0.007)	(0.009)	(0.006)
Race-Other	-0.003	-0.014	-0.008	0.005	0.004	0.005
	(0.005)	(0.009)	(0.005)	(0.007)	(0.010)	(0.006)
Race-Unknown	-0.018***	-0.054***	-0.033***	-0.025***	-0.058***	-0.038***
	(0.003)	(0.006)	(0.003)	(0.004)	(0.007)	(0.004)
Preferring Spanish	-0.009	0.016	0.004	-0.006	0.029*	0.013
	(0.008)	(0.011)	(0.007)	(0.010)	(0.013)	(0.009)
Social Vulnerability Index (SVI)	-0.001***	-0.001***	-0.001***	-0.001***	-0.001***	-0.001***
	(0.000)	(0.000)	(0.000)	(0.000)	(0.000)	(0.000)
Missing SVI	-0.038***	-0.060***	-0.045***	-0.063***	-0.104***	-0.076***
	(0.003)	(0.008)	(0.004)	(0.004)	(0.009)	(0.004)
COVID-19 Risk Score	-0.000**	0.000	-0.000	-0.000	0.000	-0.000
	(0.000)	(0.000)	(0.000)	(0.000)	(0.000)	(0.000)
Missing Risk Score	-0.027***	-0.065***	-0.039***	-0.040***	-0.077***	-0.052***
	(0.005)	(0.008)	(0.004)	(0.005)	(0.010)	(0.005)
N	27950	28183	56133	27950	28183	56133
R^2	0.031	0.047	0.063	0.035	0.045	0.072
Conditions Included	Basic Reminder w/ Video, Ownership Reminder w/ Video, and Holdout					

Legend: The table reports ordinary least squares (OLS) regressions predicting whether patients scheduled an appointment to get the first dose of the COVID-19 vaccine at UCLA Health within six days of the first reminder date (Columns 1-3) and whether patients obtained the first dose of the COVID-19 vaccine at UCLA Health within four weeks of the first reminder date (Columns 4-6), broken down by whether patients got the flu shot in either the 2019-2020 or the 2020-2021 flu season. Columns 1 and 4 focus on patients who got the flu shot, Columns 2 and 5 focus on patients who did not get the flu shot, and Columns 3 and 6 focus on the combined sample. *Video* is a dummy variable coded as 1 when participants received one of the messages containing a link to the video in the first RCT. Its p-value is < 0.001 in all columns. Its interaction with Flu Shot has a p-value of < 0.001 in Column 3 and a p-value of 0.001 in Column 6. Control variables include an indicator for whether a patient was female ("Gender-Female"), an indicator for whether a patient's gender was either other or unknown ("Gender-Other"), patient age in years ("Age"), indicators for whether a patient was Hispanic/Latino ("Hispanic"), Black ("Black"), or Asian ("Asian"), an indicator for whether a patient's race was either American Indian/Alaska Native, Native Hawaiian/Pacific Islander, or other race ("Race-Other"), an indicator for whether a patient's race was unknown ("Race-Unknown"), an indicator for whether a patient's preferred language was Spanish ("Preferring Spanish"), patient social vulnerability index ("Social Vulnerability Index (SVI)"), an indicator for whether a patient had a missing value for social vulnerability index ("Missing SVI"), patient COVID-19 risk score ("COVID-19 Risk Score"), an indicator for whether a patient had a missing value for COVID-19 risk score ("Missing Risk Score"), and batch fixed effects. "Black", "Asian", and "Race-Other" were coded as 0 if a patient was Hispanic or Latino. Robust standard errors are reported in parentheses. *p<0.05; **p<0.01; ***p<0.001 (all two-sided).

Table 10: The Additive Effects of Ownership Language and Video, Broken Down by Prior Flu Vaccination Status

Dependent Measure	Appointment at UCLA (Within Six Days)			Vaccinated at UCLA (Within Four Weeks)		
Sample	Flu Shot=0	Flu Shot=1	Both	Flu Shot=0	Flu Shot=1	Both
	(1)	(2)	(3)	(4)	(5)	(6)
Ownership	0.010***	0.020***	0.010***	0.005	0.017***	0.005
	(0.003)	(0.004)	(0.003)	(0.003)	(0.004)	(0.003)
Video	-0.001	-0.006	-0.001	0.001	-0.005	0.001
	(0.003)	(0.004)	(0.003)	(0.003)	(0.004)	(0.003)
Flu Shot			0.094***			0.118***
			(0.004)			(0.005)
Ownership x Flu Shot			0.010*			0.011*
			(0.005)			(0.005)
Video x Flu Shot			-0.005			-0.006
			(0.005)			(0.005)
Gender-Female	-0.005	-0.006	-0.005*	-0.010***	-0.008	-0.009***
	(0.003)	(0.004)	(0.002)	(0.003)	(0.004)	(0.003)
Gender-Other	-0.041***	0.228	0.007	-0.057***	0.189	-0.004
	(0.007)	(0.268)	(0.037)	(0.008)	(0.263)	(0.036)
Age	-0.002***	-0.003***	-0.002***	-0.002***	-0.003***	-0.003***
	(0.000)	(0.000)	(0.000)	(0.000)	(0.000)	(0.000)
Hispanic	0.007	0.009	0.008	0.014*	0.025**	0.021***
	(0.006)	(0.008)	(0.005)	(0.007)	(0.008)	(0.006)
Black	0.021**	0.071***	0.046***	0.041***	0.103***	0.072***
	(0.007)	(0.010)	(0.006)	(0.008)	(0.011)	(0.007)
Asian	0.002	0.016*	0.011*	0.001	0.026**	0.016**
	(0.005)	(0.008)	(0.005)	(0.006)	(0.008)	(0.005)
Race-Other	-0.009	-0.012	-0.010*	-0.013*	-0.006	-0.010
	(0.005)	(0.008)	(0.005)	(0.006)	(0.009)	(0.005)
Race-Unknown	-0.022***	-0.049***	-0.033***	-0.029***	-0.064***	-0.043***
	(0.003)	(0.006)	(0.003)	(0.004)	(0.006)	(0.003)
Preferring Spanish	-0.014	0.021*	0.004	-0.010	0.049***	0.022**
	(0.007)	(0.010)	(0.007)	(0.009)	(0.012)	(0.008)
Social Vulnerability Index (SVI)	-0.001***	-0.001***	-0.001***	-0.001***	-0.001***	-0.001***
	(0.000)	(0.000)	(0.000)	(0.000)	(0.000)	(0.000)
Missing SVI	-0.050***	-0.079***	-0.060***	-0.070***	-0.118***	-0.086***
	(0.003)	(0.007)	(0.003)	(0.004)	(0.008)	(0.004)
COVID-19 Risk Score	-0.000**	0.000*	-0.000	-0.000	0.000**	0.000
	(0.000)	(0.000)	(0.000)	(0.000)	(0.000)	(0.000)
Missing Risk Score	-0.031***	-0.065***	-0.043***	-0.044***	-0.079***	-0.054***
	(0.004)	(0.008)	(0.004)	(0.005)	(0.008)	(0.004)
N	37261	37344	74605	37261	37344	74605
R^2	0.030	0.040	0.061	0.035	0.047	0.074
Conditions Included	Conditions within the Follow-Through Reminder arm					

Legend: The table reports ordinary least squares (OLS) regressions predicting whether patients scheduled an appointment to get the first dose of the COVID-19 vaccine at UCLA Health within six days of the first reminder date (Columns 1-3) and whether patients obtained the first dose of the COVID-19 vaccine at UCLA Health within four weeks of the first reminder date (Columns 4-6), broken down by whether patients got the flu shot in either the 2019-2020 or the 2020-2021 flu season. Columns 1 and 4 focus on patients who got the flu shot, Columns 2 and 5 focus on patients who did not get the flu shot, and Columns 3 and 6 focus on the combined sample. *Ownership* is a dummy variable coded as 1 when participants received one of the messages containing the ownership language in the first RCT. Its p-value is < 0.001 in Columns 1-3 and 5 without multiple comparison adjustment and becomes < 0.002 in Columns 1-3 and 5 with a Holm-Bonferroni correction; and its p-value is 0.090 in Column 4 and 0.080 in Column 6. The interaction between *Ownership* and *Flu Shot* has a p-value of 0.045 in Column 3 and 0.037 in Column 6. *Video* is a dummy variable coded as 1 when participants received one of the messages containing a link to the video in the first RCT. Its p-value is 0.845, 0.162, 0.842, 0.764, 0.218, and 0.767 in Columns 1-6, respectively. The interaction between *Video* and *Flu Shot* has a p-value of 0.298 in Column 3 and 0.244 in Column 6. Controls include an indicator for whether a patient was female ("Gender-Female"), an indicator for whether a patient's gender was either other or unknown ("Gender-Other"), age in years ("Age"), indicators for whether a patient was Hispanic/Latino ("Hispanic"), Black ("Black"), or Asian ("Asian"), an indicator for whether a patient's race was either American Indian/Alaska Native, Native Hawaiian/Pacific Islander, or other race ("Race-Other"), an indicator for whether a patient's race was unknown ("Race-Unknown"), an indicator for whether a patient's preferred language was Spanish ("Preferring Spanish"), patient social vulnerability index ("Social Vulnerability Index (SVI)"), an indicator for whether a patient had a missing value for social vulnerability index ("Missing SVI"), patient COVID-19 risk score ("COVID-19 Risk Score"), an indicator for whether a patient had a missing value for COVID-19 risk score ("Missing Risk Score"), and batch fixed effects. "Black", "Asian", and "Race-Other" were coded as 0 if a patient was Hispanic or Latino. Robust standard errors are reported in parentheses. $^{*}p<0.05$; $^{**}p<0.01$; $^{***}p<0.001$ (all two-sided).

1.3.6 Generalizability Across Racial/Ethnic and Age Groups

We explored whether the average effect of our text message and the effect of ownership language held for both White and racial/ethnic minority patients (including Hispanic or Latino patients, Black patients, Asian patients, and patients whose race was American Indian/Alaska Native, Native Hawaiian/Pacific Islander, or other race) by applying regression specifications (1) and (5) separately to these two subgroups. Supplementary Table 11 reports these regression results.

We explored whether the average effect of our text message held for each of the three most common racial/ethnic minority groups: Hispanic/Latino, Black (excluding Hispanic/Latino), and Asian (excluding Hispanic/Latino). We applied regression specification (1) to each of these groups. Supplementary Table 12 reports the regression results.

We also explored whether the average effect of our text message and the effect of ownership language held for both patients at or above 65 years old (i.e., the vast majority of patients enrolled in the first RCT by February 23, 2021) and patients under 65 years old (to whom COVID-19 vaccines gradually became available in U.S. after the last enrollment date in our paper). We applied regression specifications (1) and (5) separately to these subgroups of patients. Supplementary Table 13 reports the results.

Table 11: Effects of Receiving a Text Reminder and Adding Ownership Language Among White Patients vs. Racial/Ethnic Minority Patients

Dependent Measure	Appointment at UCLA (Within Six Days)				Vaccinated at UCLA (Within Four Weeks)			
Sample	White	Minority	White	Minority	White	Minority	White	Minority
	(1)	(2)	(3)	(4)	(5)	(6)	(7)	(8)
Follow-Through Reminder	0.063***	0.062***			0.040***	0.033***		
	(0.003)	(0.004)			(0.004)	(0.005)		
Ownership			0.014***	0.017***			0.013***	0.008
			(0.003)	(0.005)			(0.004)	(0.005)
Video			-0.003	-0.001			-0.005	0.004
			(0.003)	(0.005)			(0.004)	(0.005)
Gender-Female	-0.008**	0.001	-0.011**	-0.000	-0.012***	-0.006	-0.013***	-0.008
	(0.003)	(0.004)	(0.003)	(0.005)	(0.003)	(0.005)	(0.004)	(0.005)
Gender-Other	0.101	-0.091***	0.097	-0.091***	0.063	-0.107***	0.059	-0.110***
	(0.170)	(0.015)	(0.169)	(0.017)	(0.162)	(0.017)	(0.164)	(0.020)
Age	-0.003***	-0.002***	-0.003***	-0.002***	-0.004***	-0.003***	-0.004***	-0.003***
	(0.000)	(0.000)	(0.000)	(0.000)	(0.000)	(0.000)	(0.000)	(0.000)
Hispanic		0.028***		0.027***		0.038***		0.039***
		(0.006)		(0.007)		(0.007)		(0.008)
Black		0.055***		0.059***		0.081***		0.086***
		(0.006)		(0.008)		(0.007)		(0.008)
Asian		0.030***		0.028***		0.032***		0.035***
		(0.005)		(0.006)		(0.006)		(0.007)
Preferring Spanish	-0.032	0.012	-0.032	0.011	0.015	0.031***	0.018	0.033***
	(0.023)	(0.007)	(0.028)	(0.008)	(0.032)	(0.008)	(0.038)	(0.009)
Social Vulnerability Index (SVI)	-0.001***	-0.001***	-0.001***	-0.001***	-0.001***	-0.001***	-0.001***	-0.001***
	(0.000)	(0.000)	(0.000)	(0.000)	(0.000)	(0.000)	(0.000)	(0.000)
Missing SVI	-0.070***	-0.066***	-0.078***	-0.072***	-0.100***	-0.113***	-0.104***	-0.115***
	(0.004)	(0.007)	(0.004)	(0.008)	(0.004)	(0.007)	(0.005)	(0.008)
COVID-19 Risk Score	-0.000	-0.000*	-0.000	-0.000	0.000	-0.000*	0.000	-0.000
	(0.000)	(0.000)	(0.000)	(0.000)	(0.000)	(0.000)	(0.000)	(0.000)
Missing Risk Score	-0.053***	-0.065***	-0.056***	-0.073***	-0.064***	-0.087***	-0.067***	-0.091***
	(0.005)	(0.007)	(0.006)	(0.008)	(0.005)	(0.008)	(0.006)	(0.009)
N	49909	29784	39811	23911	49909	29784	39811	23911
R^2	0.044	0.040	0.042	0.037	0.047	0.046	0.047	0.045
Conditions Included	All Conditions		Conditions within the Follow-Through Reminder arm		All Conditions		Conditions within the Follow-Through Reminder arm	

Legend: The table reports ordinary least squares (OLS) regressions predicting whether patients scheduled an appointment to get the first dose of the COVID-19 vaccine at UCLA Health within six days of the first reminder date (Columns 1-4) and whether patients obtained the first dose of the COVID-19 vaccine at UCLA Health within four weeks of the first reminder date (Columns 5-8). Columns 1, 3, 5, and 7 focus on White patients excluding Hispanic or Latino patients), and Columns 2, 4, 6, and 8 focus on minority patients (including Hispanic or Latino patients, Black patients, Asian patients, and patients whose race was American Indian/Alaska Native, Native Hawaiian/Pacific Islander, or other race). *Follow-Through Reminder* is a dummy variable coded as 1 when participants received one of the text reminders in the first RCT. Its p-value is < 0.001 in Columns 1-2 and 5-6. *Ownership* is a dummy variable coded as 1 when participants received one of the messages containing the ownership language in the first RCT. Its p-value is < 0.001 in Columns 3-4 and 7 without multiple comparison adjustment and becomes < 0.002 with a Holm-Bonferroni correction in Columns 3-4 and 7; and its p-value is 0.139 in Column 8. *Video* is a dummy variable coded as 1 when participants received one of the messages containing a link to the video in the first RCT. Its p-value is 0.423, 0.871, 0.163, and 0.396 in Columns 3-4 and 7-8, respectively. Control variables include an indicator for whether a patient was female (“Gender-Female”), an indicator for whether a patient’s gender was either other or unknown (“Gender-Other”), patient age in years (“Age”), indicators for whether a patient was Hispanic/Latino (“Hispanic”), Black (“Black”), or Asian (“Asian”), an indicator for whether a patient’s preferred language was Spanish (“Preferring Spanish”), patient social vulnerability index (“Social Vulnerability Index (SVI)”), an indicator for whether a patient had a missing value for social vulnerability index (“Missing SVI”), patient COVID-19 risk score (“COVID-19 Risk Score”), an indicator for whether a patient had a missing value for COVID-19 risk score (“Missing Risk Score”), and batch fixed effects. “Black” and “Asian” were coded as 0 if a patient was Hispanic or Latino. Robust standard errors are reported in parentheses. *p<0.05; **p<0.01; ***p<0.001 (all two-sided).

Table 12: Effects of Receiving a Text Reminder, Broken Down by the Three Most Common Racial/Ethnic Minority Subgroups

Dependent Measure	Appointment at UCLA (Within Six Days)			Vaccinated at UCLA (Within Four Weeks)		
Sample	Hispanic	Black	Asian	Hispanic	Black	Asian
	(1)	(2)	(3)	(4)	(5)	(6)
Follow-Through Reminder	0.059***	0.084***	0.051***	0.033***	0.048***	0.034**
	(0.007)	(0.011)	(0.009)	(0.009)	(0.014)	(0.010)
Gender-Female	-0.000	0.009	0.012	-0.010	-0.001	0.011
	(0.007)	(0.010)	(0.008)	(0.008)	(0.012)	(0.009)
Gender-Other			-0.105***			-0.151***
			(0.021)			(0.024)
Age	-0.001*	-0.001	-0.003***	-0.001***	-0.002**	-0.004***
	(0.000)	(0.000)	(0.000)	(0.000)	(0.001)	(0.001)
Race-Unknown	-0.022			-0.026		
	(0.012)			(0.015)		
Preferring Spanish	0.009	-0.214***	0.524	0.024**	-0.295***	0.814***
	(0.007)	(0.027)	(0.291)	(0.008)	(0.030)	(0.027)
Social Vulnerability Index (SVI)	-0.001***	-0.001***	-0.001***	-0.001***	-0.001***	-0.001***
	(0.000)	(0.000)	(0.000)	(0.000)	(0.000)	(0.000)
Missing SVI	-0.082***	-0.046*	-0.077***	-0.130***	-0.120***	-0.112***
	(0.013)	(0.019)	(0.014)	(0.015)	(0.020)	(0.015)
COVID-19 Risk Score	-0.000	-0.000	-0.000	-0.000	-0.001	-0.000
	(0.000)	(0.000)	(0.000)	(0.000)	(0.000)	(0.000)
Missing Risk Score	-0.078***	-0.058*	-0.084***	-0.106***	-0.102***	-0.082***
	(0.013)	(0.026)	(0.014)	(0.015)	(0.028)	(0.016)
N	10624	5109	7553	10624	5109	7553
R^2	0.040	0.034	0.059	0.046	0.044	0.060

Legend: The table reports ordinary least squares (OLS) regressions predicting whether patients scheduled an appointment to get the first dose of the COVID-19 vaccine at UCLA Health within six days of the first reminder date (Columns 1-3) and whether patients obtained the first dose of the COVID-19 vaccine at UCLA Health within four weeks of the first reminder date (Columns 4-6). Columns 1 and 4 focus on Hispanic patients, Columns 2 and 5 focus on Black patients (excluding Hispanic or Latino patients), and Columns 3 and 6 focus on Asian patients (excluding Hispanic or Latino patients). *Follow-Through Reminder* is a dummy variable coded as 1 when participants received one of the text reminders in the first RCT. Its p-value is < 0.001 in Columns 1-5 and equals 0.001 in Column 6. Control variables include an indicator for whether a patient was female ("Gender-Female"), an indicator for whether a patient's gender was either other or unknown ("Gender-Other"), patient age in years ("Age"), an indicator for whether a patient's race was unknown ("Race-Unknown"; only in Columns 1 and 4), an indicator for whether a patient's preferred language was Spanish ("Preferring Spanish"), patient social vulnerability index ("Social Vulnerability Index (SVI)"), an indicator for whether a patient had a missing value for social vulnerability index ("Missing SVI"), patient COVID-19 risk score ("COVID-19 Risk Score"), an indicator for whether a patient had a missing value for COVID-19 risk score ("Missing Risk Score"), and batch fixed effects. Robust standard errors are reported in parentheses. *p<0.05; **p<0.01; ***p<0.001 (all two-sided).

Table 13: Effects of Receiving a Text Reminder and Adding Ownership Language, Broken Down by Whether or Not Patients Were at Least 65 Years Old

Dependent Measure	Appointment at UCLA (Within Six Days)				Vaccinated at UCLA (Within Four Weeks)			
Sample	Age $\geq$ 65	Age $<$ 65	Age $\geq$ 65	Age $<$ 65	Age $\geq$ 65	Age $<$ 65	Age $\geq$ 65	Age $<$ 65
	(1)	(2)	(3)	(4)	(5)	(6)	(7)	(8)
Follow-Through Reminder	0.057***	0.099***			0.034***	0.053***		
	(0.002)	(0.010)			(0.003)	(0.011)		
Ownership			0.012***	0.041***			0.009**	0.028**
			(0.002)	(0.010)			(0.003)	(0.011)
Video			-0.002	-0.006			-0.002	-0.002
			(0.002)	(0.010)			(0.003)	(0.011)
Gender-Female	-0.001	-0.017	-0.003	-0.021*	-0.004	-0.028**	-0.005	-0.033**
	(0.002)	(0.009)	(0.003)	(0.010)	(0.002)	(0.010)	(0.003)	(0.011)
Gender-Other	-0.053***	0.768***	-0.072***	0.793***	-0.073***	0.741***	-0.090***	0.748***
	(0.010)	(0.016)	(0.009)	(0.020)	(0.011)	(0.018)	(0.012)	(0.021)
Age	-0.003***	0.002***	-0.004***	0.002***	-0.004***	0.002***	-0.004***	0.003***
	(0.000)	(0.000)	(0.000)	(0.000)	(0.000)	(0.000)	(0.000)	(0.001)
Hispanic	0.011*	0.012	0.011*	0.009	0.020***	0.037*	0.021***	0.024
	(0.004)	(0.013)	(0.005)	(0.015)	(0.005)	(0.014)	(0.006)	(0.016)
Black	0.046***	-0.024	0.052***	-0.026	0.072***	0.008	0.074***	0.004
	(0.006)	(0.016)	(0.007)	(0.018)	(0.007)	(0.018)	(0.007)	(0.020)
Asian	0.011**	0.045*	0.011*	0.035	0.014**	0.061**	0.015**	0.048*
	(0.004)	(0.018)	(0.005)	(0.020)	(0.005)	(0.019)	(0.005)	(0.021)
Ethnicity-Other	-0.015***	-0.027	-0.016***	-0.018	-0.012*	-0.032	-0.016**	-0.027
	(0.004)	(0.018)	(0.005)	(0.021)	(0.005)	(0.019)	(0.005)	(0.022)
Ethnicity-Unknown	-0.033***	-0.078***	-0.034***	-0.078***	-0.042***	-0.079***	-0.045***	-0.078***
	(0.003)	(0.015)	(0.003)	(0.017)	(0.003)	(0.016)	(0.004)	(0.018)
Preferring Spanish	0.008	-0.013	0.006	-0.015	0.020**	0.019	0.020*	0.023
	(0.006)	(0.017)	(0.007)	(0.019)	(0.007)	(0.020)	(0.008)	(0.023)
Social Vulnerability Index (SVI)	-0.001***	-0.002***	-0.001***	-0.002***	-0.001***	-0.002***	-0.001***	-0.002***
	(0.000)	(0.000)	(0.000)	(0.000)	(0.000)	(0.000)	(0.000)	(0.000)
Missing SVI	-0.056***	-0.153***	-0.063***	-0.171***	-0.087***	-0.203***	-0.090***	-0.214***
	(0.003)	(0.016)	(0.003)	(0.018)	(0.003)	(0.017)	(0.004)	(0.019)
COVID-19 Risk Score	-0.000*	-0.000	-0.000	-0.000	-0.000*	0.000	-0.000*	0.000
	(0.000)	(0.000)	(0.000)	(0.000)	(0.000)	(0.000)	(0.000)	(0.000)
Missing Risk Score	-0.037***	-0.084***	-0.039***	-0.089***	-0.046***	-0.103***	-0.048***	-0.107***
	(0.004)	(0.013)	(0.004)	(0.015)	(0.004)	(0.015)	(0.005)	(0.016)
N	84075	9279	67115	7490	84075	9279	67115	7490
R^2	0.034	0.054	0.032	0.048	0.044	0.042	0.044	0.041
Conditions Included	All Conditions		Conditions within the Follow-Through Reminder arm		All Conditions		Conditions within the Follow-Through Reminder arm	

Legend: The table reports ordinary least squares (OLS) regressions predicting whether patients scheduled an appointment to get the first dose of the COVID-19 vaccine at UCLA Health within six days of the first reminder date (Columns 1-4) and whether patients obtained the first dose of the COVID-19 vaccine at UCLA Health within four weeks of the first reminder date (Columns 5-8). Columns 1, 3, 5, and 7 focus on patients at or above 65 years old, and Columns 2, 4, 6, and 8 focus on patients under 65 years old. *Follow-Through Reminder* is a dummy variable coded as 1 when participants received one of the text reminders in the first RCT. Its p-value is $<$ 0.001 in Columns 1-2 and 5-6. *Ownership* is a dummy variable coded as 1 when participants received one of the messages containing the ownership language in the first RCT. Its p-value is $<$ 0.001 in Columns 3-4, 0.001 in Column 7, and 0.007 in Column 8 without multiple comparison adjustment; and its p-value becomes $<$ 0.002 in Columns 3-4, 0.002 in Column 7, and 0.014 in Column 8 with a Holm-Bonferroni correction. *Video* is a dummy variable coded as 1 when participants received one of the messages containing a link to the video in the first RCT. Its p-value is 0.326, 0.521, 0.486, and 0.838 in Columns 3-4 and 7-8, respectively. Control variables include an indicator for whether a patient was female ("Gender-Female"), an indicator for whether a patient's gender was either other or unknown ("Gender-Other"), patient age in years ("Age"), indicators for whether a patient was Hispanic/Latino ("Hispanic"), Black ("Black"), or Asian ("Asian"), an indicator for whether a patient's race was either American Indian/Alaska Native, Native Hawaiian/Pacific Islander, or other race ("Race-Other"), an indicator for whether a patient's race was unknown ("Race-Unknown"), an indicator for whether a patient's preferred language was Spanish ("Preferring Spanish"), patient social vulnerability index ("Social Vulnerability Index (SVI)"), an indicator for whether a patient had a missing value for social vulnerability index ("Missing SVI"), patient COVID-19 risk score ("COVID-19 Risk Score"), an indicator for whether a patient had a missing value for COVID-19 risk score ("Missing Risk Score"), and batch fixed effects. "Black", "Asian", and "Race-Other" were coded as 0 if a patient was Hispanic or Latino. Robust standard errors are reported in parentheses. *p<0.05; **p<0.01; ***p<0.001 (all two-sided).

1.3.7 Pre-registered Scope of Analyses After Data Collection Has Been Completed

Below, we detail the scope of the analyses we pre-registered to do after all UCLA patients have been invited (or if vaccine distribution plan changes and UCLA Health no longer sends out text messages to patients at some point; see https://clinicaltrials.gov/ct2/show/record/NCT04800965).

1. If the additional data collected after February 23, 2021 (the last enrollment date for this paper) exceeds 30,000 patients, then we will analyze the main effect of sending a text reminder (vs. Holdout) and report the raw data for each sub-arm to see if the patterns for the subsequent sample are qualitatively comparable with findings in this paper. $30,000$ was selected as a cutoff point because it would give us 80% power to detect a 2-percentage-point difference between the *Holdout* arm and the *Follow-Through Reminder* arm, assuming that the *Holdout* arm has a 50% baseline.

2. We will use the full sample (including patients enrolled by and after February 23, 2021) to:

    (a) test the interaction between the video intervention and the ownership intervention–that is, to analyze whether combining video and ownership language outperforms video alone or ownership language alone;

    (b) analyze the heterogeneous treatment effects based on gender, race/ethnicity, preferred language (Spanish vs. not), age (under 65 or not), health status (social vulnerability index, COVID risk score, population risk score), marital status, prior flu vaccination status, the day of the week when the text message was sent to a patient, and how strongly a patient's neighborhood (defined by zipcode) is in favor of the Republican (vs. Democratic) Party.

1.4 Analyses and Results about the Second RCT

1.4.1 Pre-registered Scope of Analyses about Patients Enrolled by February 23, 2021

Since the sample of patients who were enrolled in the second RCT by February 23, 2021 and met our inclusion/exclusion criteria exceeds 40,000 (we selected this threshold because it would give us 80% power to detect a 2-percentage-point difference between the *Holdout* arm and the *Follow-Through Reminder* arm, assuming that the *Holdout* arm has a 50% baseline), we followed the pre-registration (see the Tabular View tab at https://www.clinicaltrials.gov/ct2/show/record/NCT04801524) to answer the following questions about the second RCT in this paper:

1. What is the average effect of sending behaviorally informed text reminders among patients in the second RCT?

2. Do all types of text messages—including the three messages mentioning personal benefits, the three messages emphasizing prosocial benefits, the two messages highlighting exclusive access to the vaccine, and the two messages referencing the opportunity for a fresh start—all outperform the *Holdout* arm?

Because we were uncertain about how many people would be qualified for the second RCT's inclusion/exclusion criteria by February 23, 2021, we were concerned that we might not have enough power to test the effects of prosocial (vs. self) messaging, the effects of exclusivity (vs. basic) framing, the effects of fresh start (vs. basic) framing, as well as their interactions. Thus, we pre-registered to only answer those questions and conduct hypothesis testing between sub-arms within the *Follow-Through Reminder* arm once data collection has been completed.

In addition to reporting regression analyses, we also pre-registered that we would show the raw data for each condition without conducting hypothesis testing. Supplementary Figure 2 displays the average appointment rate and the average vaccination rate at UCLA Health in each of the seven conditions (six sub-arms within the *Follow-Through Reminder* arm plus the *Holdout* arm).

Similar to the first RCT, we employed a two-stage gatekeeping design[50] to control the familywise type I error rate for our primary analyses at the 5% level. Specifically, we planned to first compare the combined *Follow-through Reminder* arm with the *Holdout* arm for each outcome variable and, if this comparison leads to statistically significant differences, we would next evaluate the difference between each type of message and the *Holdout* arm to explore whether every type of message consistently outperforms the *Holdout* arm.

1.4.2 The Average Effect of Receiving the Second Text Reminder

We used the same OLS regression specification as equation (1) to estimate the average effect of receiving the second text reminder at UCLA Health. Supplementary Table 14 reports the results.

Among patients in the *Follow-Through Reminder* arm of the second RCT, only 20.98% were in the *Holdout* arm of the first RCT, to whom the text message sent in the second RCT was the first reminder they received from UCLA Health. Thus, Supplementary Table 14 Columns 3-4 report regression results after we exclude from our analysis patients in the *Holdout* arm of the first RCT.

Table 14: Effects of Receiving the Second Text Reminder on Appointments and Vaccinations at UCLA Health

Dependent Measure	Appointment at UCLA (Within Six Days)		Vaccinated at UCLA (Within Four Weeks)	
Sample	All Patients	Excl. Patients in the Holdout Arm of the first RCT	All Patients	Excl. Patients in the Holdout Arm of the first RCT
	(1)	(2)	(3)	(4)
Follow-Through Reminder	0.017***	0.014***	0.011***	0.010***
	(0.002)	(0.002)	(0.003)	(0.003)
Gender-Female	-0.002	-0.001	-0.004	-0.003
	(0.002)	(0.002)	(0.002)	(0.002)
Gender-Other	-0.019***	-0.018***	-0.030***	-0.031***
	(0.004)	(0.004)	(0.005)	(0.006)
Age	-0.001***	-0.001***	-0.001***	-0.001***
	(0.000)	(0.000)	(0.000)	(0.000)
Hispanic	0.016***	0.012***	0.023***	0.022***
	(0.004)	(0.004)	(0.004)	(0.005)
Black	0.017***	0.014**	0.039***	0.037***
	(0.004)	(0.004)	(0.005)	(0.006)
Asian	0.007*	0.007	0.012**	0.013**
	(0.003)	(0.004)	(0.004)	(0.004)
Race-Other	-0.001	-0.003	0.005	0.003
	(0.003)	(0.003)	(0.004)	(0.004)
Race-Unknown	-0.011***	-0.012***	-0.014***	-0.016***
	(0.002)	(0.002)	(0.002)	(0.003)
Preferring Spanish	0.008	0.008	0.018**	0.022**
	(0.005)	(0.005)	(0.006)	(0.007)
Social Vulnerability Index (SVI)	-0.000***	-0.000***	-0.000***	-0.000***
	(0.000)	(0.000)	(0.000)	(0.000)
Missing SVI	-0.029***	-0.026***	-0.046***	-0.043***
	(0.002)	(0.002)	(0.002)	(0.002)
COVID-19 Risk Score	-0.000**	-0.000*	-0.000***	-0.000***
	(0.000)	(0.000)	(0.000)	(0.000)
Missing Risk Score	-0.021***	-0.020***	-0.027***	-0.027***
	(0.003)	(0.003)	(0.003)	(0.003)
N	67092	53007	67092	53007
R^2	0.011	0.010	0.021	0.020

Legend: The table reports ordinary least squares (OLS) regressions predicting whether patients scheduled an appointment to get the first dose of the COVID-19 vaccine at UCLA Health within six days of the second reminder date (Columns 1-2) and whether patients obtained the first dose of the COVID-19 vaccine at UCLA Health within four weeks of the second reminder date (Columns 3-4). Columns 1 and 3 include all patients in the analysis sample of the second RCT. Columns 2 and 4 exclude patients who were in the *Holdout* arm of the first RCT. The primary predictor, *Follow-Through Reminder*, is a dummy variable coded as 1 when patients received one of the text reminders in the second RCT. Its p-value is < 0.001 in all columns. Control variables include an indicator for whether a patient was female ("Gender-Female"), an indicator for whether a patient's gender was either other or unknown ("Gender-Other"), patient age in years ("Age"), indicators for whether a patient was Hispanic/Latino ("Hispanic"), Black ("Black"), or Asian ("Asian"), an indicator for whether a patient's race was either American Indian/Alaska Native, Native Hawaiian/Pacific Islander, or other race ("Race-Other"), an indicator for whether a patient's race was unknown ("Race-Unknown"), an indicator for whether a patient's preferred language was Spanish ("Preferring Spanish"), patient social vulnerability index ("Social Vulnerability Index (SVI)"), an indicator for whether a patient had a missing value for social vulnerability index ("Missing SVI"), patient COVID-19 risk score ("COVID-19 Risk Score"), an indicator for whether a patient had a missing value for COVID-19 risk score ("Missing Risk Score"), and batch fixed effects. "Black", "Asian", and "Race-Other" were coded as 0 if a patient was Hispanic or Latino. Robust standard errors are reported in parentheses. *p<0.05; **p<0.01; ***p<0.001 (all two-sided).

1.4.3 Comparing Different Types of Second Text Reminders with Holdout

To test whether all types of messages—the three reminders mentioning personal benefits, the three reminders emphasizing prosocial benefits, the two reminders highlighting patients' early access to the vaccine, and the two reminders referencing the opportunity for a fresh start—outperformed the *Holdout* arm, we conducted four sets of regressions:

$$Y_i = \beta_0 + \beta_1 \mathit{Self}_i + \beta_2 X_i + \epsilon_i, \tag{6}$$

Specification (6) only includes patients in the *Holdout* arm, the *Basic Self* condition, the *Early Access Self* condition, and the *Fresh Start Self* condition. Y_i represents the outcome variable of patient i. $Self_i$ equals 1 if patient i was assigned to the three latter conditions that highlighted personal benefits and 0 otherwise. X_i represents a host of control variables listed in Supplementary Information Section 1.2. We report robust standard errors.

$$Y_i = \beta_0 + \beta_1 \mathit{Prosocial}_i + \beta_2 X_i + \epsilon_i, \tag{7}$$

Specification (7) only includes patients in the *Holdout* arm, the *Basic Prosocial* condition, the *Early Access Prosocial* condition, and the *Fresh Start Prosocial* condition. Y_i represents the outcome variable of patient i. $Prosocial_i$ equals 1 if patient i was assigned to the three latter conditions that highlighted prosocial benefits and 0 otherwise. X_i represents a host of control variables listed in Supplementary Information Section 1.2. We report robust standard errors.

$$Y_i = \beta_0 + \beta_1 \mathit{Early\ Access}_i + \beta_2 X_i + \epsilon_i, \tag{8}$$

Specification (8) only includes patients in the *Holdout* arm, the *Early Access Self* condition, and the *Early Access Prosocial* condition. Y_i represents the outcome variable of patient i. $\mathit{Early\ Access}_i$ equals 1 if patient i was assigned to the two latter conditions that highlighted their early access to the vaccine and 0, otherwise. X_i represents a host of control variables listed in Supplementary Information Section 1.2. We report robust standard errors.

$$Y_i = \beta_0 + \beta_1 \mathit{Fresh\ Start}_i + \beta_2 X_i + \epsilon_i, \tag{9}$$

Specification (9) only includes patients in the *Holdout* arm, the *Fresh Start Self* condition, and the *Fresh Start Prosocial* condition. Y_i represents the outcome variable of patient i. $\mathit{Fresh\ Start}_i$ equals 1 if patient i was assigned to the two latter conditions that framed getting the vaccine as an opportunity for having a fresh start and 0, otherwise. X_i represents a host of control variables listed in Supplementary Information Section 1.2. We report robust standard errors.

Supplementary Table 15 reports the results from specifications (6)-(9).

Table 15: Effects of Different Types of Second Text Reminders on Appointments and Vaccinations at UCLA Health

Dependent Measure	Appointment at UCLA (Within Six Days)				Vaccinated at UCLA (Within Four Weeks)			
	(1)	(2)	(3)	(4)	(5)	(6)	(7)	(8)
Self	0.015***				0.009**			
	(0.002)				(0.003)			
Prosocial		0.018***				0.013***		
		(0.002)				(0.003)		
Early Access			0.020***				0.013***	
			(0.002)				(0.003)	
Fresh Start				0.013***				0.009**
				(0.002)				(0.003)
Gender-Female	-0.002	-0.001	-0.001	-0.001	-0.004	-0.003	-0.003	-0.003
	(0.002)	(0.002)	(0.002)	(0.002)	(0.003)	(0.003)	(0.003)	(0.003)
Gender-Other	-0.019***	-0.015**	-0.013*	-0.008	-0.030***	-0.027***	-0.028***	-0.019**
	(0.005)	(0.005)	(0.006)	(0.006)	(0.006)	(0.006)	(0.007)	(0.007)
Age	-0.001***	-0.001***	-0.001***	-0.001***	-0.001***	-0.001***	-0.002***	-0.001***
	(0.000)	(0.000)	(0.000)	(0.000)	(0.000)	(0.000)	(0.000)	(0.000)
Hispanic	0.015***	0.017***	0.016**	0.017**	0.023***	0.025***	0.022***	0.022***
	(0.005)	(0.005)	(0.005)	(0.005)	(0.006)	(0.006)	(0.007)	(0.006)
Black	0.016**	0.014*	0.017**	0.013*	0.041***	0.038***	0.036***	0.039***
	(0.005)	(0.005)	(0.006)	(0.006)	(0.007)	(0.007)	(0.008)	(0.008)
Asian	0.005	0.009*	0.016**	0.002	0.008	0.015**	0.016*	0.011
	(0.004)	(0.004)	(0.005)	(0.004)	(0.005)	(0.005)	(0.006)	(0.006)
Race-Other	-0.004	0.002	-0.004	0.001	0.003	0.007	0.001	0.009
	(0.004)	(0.004)	(0.004)	(0.004)	(0.005)	(0.005)	(0.006)	(0.006)
Race-Unknown	-0.012***	-0.010***	-0.010***	-0.008**	-0.013***	-0.015***	-0.013***	-0.012**
	(0.003)	(0.003)	(0.003)	(0.003)	(0.003)	(0.003)	(0.004)	(0.004)
Preferring Spanish	0.000	0.010	0.002	0.010	0.007	0.024**	0.023*	0.017
	(0.006)	(0.006)	(0.007)	(0.007)	(0.008)	(0.008)	(0.009)	(0.009)
Social Vulnerability Index (SVI)	-0.000***	-0.000***	-0.000***	-0.000***	-0.000***	-0.001***	-0.001***	-0.000***
	(0.000)	(0.000)	(0.000)	(0.000)	(0.000)	(0.000)	(0.000)	(0.000)
Missing SVI	-0.027***	-0.027***	-0.029***	-0.026***	-0.047***	-0.042***	-0.042***	-0.046***
	(0.002)	(0.002)	(0.003)	(0.003)	(0.003)	(0.003)	(0.003)	(0.003)
COVID-19 Risk Score	-0.000**	-0.000	-0.000	-0.000*	-0.000**	-0.000**	-0.000*	-0.000**
	(0.000)	(0.000)	(0.000)	(0.000)	(0.000)	(0.000)	(0.000)	(0.000)
Missing Risk Score	-0.020***	-0.022***	-0.016***	-0.015***	-0.027***	-0.026***	-0.022***	-0.024***
	(0.003)	(0.003)	(0.004)	(0.004)	(0.004)	(0.004)	(0.004)	(0.004)
N	38406	38311	28765	28759	38406	38311	28765	28759
R^2	0.010	0.012	0.013	0.010	0.021	0.022	0.024	0.020
Conditions Included	Self: Basic Early Access Fresh Start Holdout	Prosocial: Basic Early Access Fresh Start Holdout	Early Access: Self Prosocial Holdout	Fresh Start: Self Prosocial Holdout	Self: Basic Early Access Fresh Start Holdout	Prosocial: Basic Early Access Fresh Start Holdout	Early Access: Self Prosocial Holdout	Fresh Start: Self Prosocial Holdout

Legend: The table reports OLS regressions predicting whether patients scheduled an appointment to get the first dose of the COVID-19 vaccine at UCLA Health within six days of the second reminder date (Columns 1-4) and whether patients obtained the first dose at UCLA Health within four weeks (Columns 5-8). *Self* is a dummy variable coded as 1 when patients received one of the text messages highlighting personal benefits (its p-value is $<$ 0.001 in Column 1 and 0.003 in Column 5). *Prosocial* is a dummy variable coded as 1 when patients received one of the text messages highlighting prosocial benefits (its p-value is $<$ 0.001 in Columns 2 and 6). *Early Access* is a dummy variable coded as 1 when patients received one of the text messages highlighting their early access to the vaccine (its p-value is $<$ 0.001 in Columns 3 and 7). *Fresh Start* is a dummy variable coded as 1 when patients received one of the text messages framing getting vaccinated as an opportunity for a fresh start (its p-value is $<$ 0.001 in Column 4 and 0.004 in Column 8). Controls include an indicator for whether a patient was female ("Gender-Female"), an indicator for whether a patient's gender was either other or unknown ("Gender-Other"), patient age in years ("Age"), indicators for whether a patient was Hispanic/Latino ("Hispanic"), Black ("Black"), or Asian ("Asian"), an indicator for whether a patient's race was either American Indian/Alaska Native, Native Hawaiian/Pacific Islander, or other race ("Race-Other"), an indicator for whether a patient's race was unknown ("Race-Unknown"), an indicator for whether a patient's preferred language was Spanish ("Preferring Spanish"), patient social vulnerability index ("Social Vulnerability Index (SVI)"), an indicator for whether a patient had a missing value for social vulnerability index ("Missing SVI"), patient COVID-19 risk score ("COVID-19 Risk Score"), an indicator for whether a patient had a missing value for COVID-19 risk score ("Missing Risk Score"), and batch fixed effects. "Black", "Asian", and "Race-Other" were coded as 0 if a patient was Hispanic or Latino. Robust standard errors are reported in parentheses. *p<0.05; **p<0.01; ***p<0.001 (all two-sided).

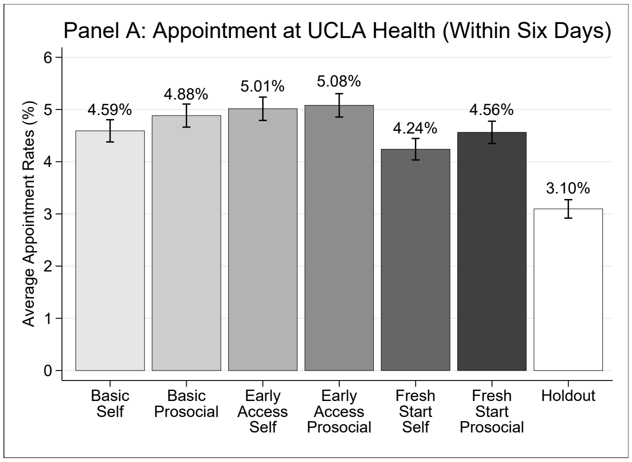

(a) Panel A

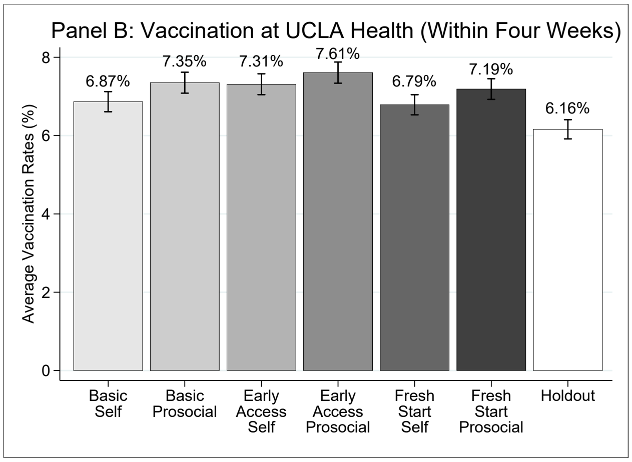

(b) Panel B

Figure 2: Appointment and Vaccination Rates by Condition (Second RCT)

Figure Legend: The average appointment and vaccination rates at UCLA Health for each condition of the second RCT are depicted here. Panel A displays the proportion of patients in each condition who scheduled an appointment for the first dose of the COVID-19 vaccine at UCLA Health between 3pm PST on the second reminder data and the end of the fifth day later. Panel B displays the proportion of patients in each condition who obtained the first dose of the COVID-19 vaccine at UCLA Health within four weeks of the second reminder date. In both panels, error bars represent ±1 standard error of the mean. The number of observations in each condition (from left to right in each panel) is 9671, 9522, 9533, 9607, 9575, 9557, and 9625, respectively.

1.4.4 Pre-registered Scope of Analyses After Data Collection Has Been Completed

Below, we detail the scope of the analyses we pre-registered to do after all UCLA patients have been invited (or if vaccine distribution plan changes and UCLA Health no longer sends out text messages to patients at some point; see https://www.clinicaltrials.gov/ct2/show/record/NCT04801524).

1. If the additional data collected after February 23, 2021 (the last enrollment date for this paper) exceeds 40,000 patients (this would give us 80% power to detect a 2-percentage-point difference between the *Holdout* arm and the *Follow-Through Reminder* arm, assuming that the *Holdout* arm has a 50% baseline), then we will analyze the main effect of sending a text reminder (vs. Holdout) and report the raw data for each sub-arm to see if the patterns for the subsequent sample are qualitatively comparable with findings in this paper.

2. We will use the full sample (including patients enrolled by and after February 23, 2021) to analyze:

    (a) the effect of highlighting prosocial benefits (vs. self-benefits);

    (b) the effect of highlighting early access (vs. not highlighting it);

    (c) the effect of highlighting the promise of a fresh start (vs. not highlighting it);

    (d) whether the combination of early access and prosocial benefits will outperform early access alone or prosocial benefits alone;

(e) whether the combination of fresh start and prosocial benefits will outperform fresh start alone or prosocial benefits alone;

(f) the heterogeneous treatment effects based on gender, race, preferred language (Spanish vs. not), age (under 65 or not), health status (social vulnerability index score, COVID risk score, population risk score), marital status, prior flu vaccination status, the day of the week when the text message was sent to a patient, how strongly a patient's neighborhood (defined by zipcode) is in favor of the Republican (vs. Democratic) Party, the condition that each participant was assigned to in the first RCT, the number of days between when the first batch of patients received the initial invitation from UCLA Health and when a patient in question received the initial invitation, as well as the number of patients who had received the initial invitation from UCLA Health before a patient in question received the initial invitation.

1.5 Robustness Checks

In this section, we report the following robustness checks: (1) using all patients who were enrolled in the first (second) RCT by February 23, 2021 (i.e., the intent-to-treat analysis) except that we had to exclude patients who were under 18 years old given that the Institutional Review Board approved our analysis of adult patients (Supplementary Tables 16-17); (2) dropping control variables (Supplementary Tables 18-19); (3) conducting logistic regressions (Supplementary Tables 20-21); and (4) assessing whether patients scheduled the first-dose appointment at UCLA Health within four weeks after 3pm PST on the first (second) reminder date and whether patients obtained the first dose anywhere within four weeks of the first (second) reminder date according to vaccination records we extracted on May 25, 2021 (Supplementary Tables 22-23).

To shed some light on why the effect of receiving the second text reminder on vaccination rates anywhere was not significant in four weeks (B = 0.007, SE = 0.004, p = 0.127 and B = 0.006, SE = 0.005, p = 0.219 in Columns 3 and 4, respectively, of Supplementary Table 23), we plotted the Kaplan-Meier curves to capture the percentage of patients who had obtained the first dose anywhere by a given day within four weeks after their second reminder date (Supplementary Figure 3). Supplementary Figure 3 shows that in the second RCT, the gap between the *Follow-Through Reminder* arm and the *Holdout* arm emerged around 10 days after the second reminder, maintained the same size up to the 15th day, decreased afterward, and disappeared at the end of four weeks. Inspired by this figure, we constructed one binary outcome variable for patients in the second RCT, *Vaccination Anywhere Within Two Weeks*, which took a value of 1 if patients obtained the first dose anywhere within two weeks of their second reminder date and 0 otherwise. As shown in Table 24, receiving the second text reminder on average significantly boosted patients' likelihood of getting the first dose anywhere within two weeks of the second reminder date by 0.95 percentage points (B = 0.010, SE = 0.004, p = 0.008), from a baseline of 12.04 percentage points in the *Holdout* arm. Altogether, even though vaccination rates by the end of four weeks did not statistically significantly differ between the *Follow-Through Reminder* arm and the *Holdout* arm at the 5% level, we did observe statistically significant differences in vaccination rates early on, suggesting that receiving the second text reminder could accelerate vaccinations.

Table 16: Effects of Receiving a Text Reminder and Adding Ownership Language (Intent-to-Treat Analysis)

Dependent Measure	Appointment at UCLA (Within Six Days)		Vaccinated at UCLA (Within Four Weeks)	
	(1)	(2)	(3)	(4)
Follow-Through Reminder	0.042***		0.018***	
	(0.002)		(0.002)	
Ownership		0.011***		0.009***
		(0.002)		(0.002)
Video		-0.002		-0.000
		(0.002)		(0.002)
Gender-Female	-0.004*	-0.005**	-0.012***	-0.013***
	(0.002)	(0.002)	(0.002)	(0.002)
Gender-Other	-0.008	-0.011	-0.031	-0.035
	(0.031)	(0.041)	(0.027)	(0.036)
Age	-0.002***	-0.002***	-0.003***	-0.003***
	(0.000)	(0.000)	(0.000)	(0.000)
Hispanic	0.017***	0.018***	0.029***	0.029***
	(0.003)	(0.004)	(0.004)	(0.005)
Black	0.041***	0.046***	0.073***	0.075***
	(0.005)	(0.005)	(0.006)	(0.006)
Asian	0.009**	0.008*	0.005	0.005
	(0.003)	(0.004)	(0.004)	(0.004)
Race-Other	-0.007*	-0.006	-0.017***	-0.017***
	(0.003)	(0.004)	(0.004)	(0.004)
Race-Unknown	-0.027***	-0.027***	-0.046***	-0.047***
	(0.002)	(0.002)	(0.002)	(0.003)
Preferring Spanish	0.012*	0.012*	0.020***	0.023**
	(0.005)	(0.006)	(0.006)	(0.007)
Social Vulnerability Index (SVI)	-0.000***	-0.001***	-0.001***	-0.001***
	(0.000)	(0.000)	(0.000)	(0.000)
Missing SVI	-0.038***	-0.042***	-0.076***	-0.079***
	(0.003)	(0.003)	(0.003)	(0.004)
COVID-19 Risk Score	0.000	0.000	0.000	0.000
	(0.000)	(0.000)	(0.000)	(0.000)
Missing Risk Score	-0.044***	-0.047***	-0.073***	-0.077***
	(0.002)	(0.003)	(0.003)	(0.003)
N	132277	105834	132277	105834
R^2	0.033	0.031	0.060	0.060
Conditions Included	All Conditions	Conditions within the Follow-Through Reminder arm	All Conditions	Conditions within the Follow-Through Reminder arm

Legend: The table reports ordinary least squares (OLS) regressions predicting whether patients scheduled an appointment to get the first dose of the COVID-19 vaccine at UCLA Health within six days of the first reminder date (Columns 1-2) and whether patients obtained the first dose of the COVID-19 vaccine at UCLA Health within four weeks of the first reminder date (Columns 3-4). All columns include patients who were enrolled in the first RCT by February 23, 2021 and were in one of the conditions marked in the last row of the table, regardless of whether they fit the inclusion/exclusion criteria for the analysis sample. *Follow-Through Reminder* is a dummy variable coded as 1 when participants received one of the text reminders in the first RCT. Its p-value is < 0.001 in Columns 1 and 3. *Ownership* is a dummy variable coded as 1 when participants received one of the messages containing the ownership language in the first RCT. Its p-value is < 0.001 in Columns 2 and 4 without multiple comparison adjustment; and its p-value becomes < 0.002 with a Holm-Bonferroni correction. *Video* is a dummy variable coded as 1 when participants received one of the messages containing a link to the video in the first RCT. Its p-value is 0.321 and 0.950 in Columns 2 and 4, respectively. Control variables include an indicator for whether a patient was female ("Gender-Female"), an indicator for whether a patient's gender was either other or unknown ("Gender-Other"), patient age in years ("Age"), indicators for whether a patient was Hispanic/Latino ("Hispanic"), Black ("Black"), or Asian ("Asian"), an indicator for whether a patient's race was either American Indian/Alaska Native, Native Hawaiian/Pacific Islander, or other race ("Race-Other"), an indicator for whether a patient's race was unknown ("Race-Unknown"), an indicator for whether a patient's preferred language was Spanish ("Preferring Spanish"), patient social vulnerability index ("Social Vulnerability Index (SVI)"), an indicator for whether a patient had a missing value for social vulnerability index ("Missing SVI"), patient COVID-19 risk score ("COVID-19 Risk Score"), an indicator for whether a patient had a missing value for COVID-19 risk score ("Missing Risk Score"), and batch fixed effects. "Black", "Asian", and "Race-Other" were coded as 0 if a patient was Hispanic or Latino. Robust standard errors are reported in parentheses. $^{*}p<0.05$; $^{**}p<0.01$; $^{***}p<0.001$ (all two-sided).

Table 17: Effects of Receiving the Second Text Reminder (Intent-to-Treat Analysis)

Dependent Measure	Appointment at UCLA (Within Six Days)		Vaccinated at UCLA (Within Four Weeks)	
Sample	All Patients	Excl. Patients in the Holdout Arm of the first RCT	All Patients	Excl. Patients in the Holdout Arm of the first RCT
	(1)	(2)	(3)	(4)
Follow-Through Reminder	0.012***	0.010***	0.007***	0.007***
	(0.002)	(0.001)	(0.002)	(0.002)
Gender-Female	-0.001	-0.001	-0.002	-0.002
	(0.001)	(0.001)	(0.001)	(0.001)
Gender-Other	-0.014	-0.014***	-0.021	-0.022***
	(0.035)	(0.003)	(0.043)	(0.004)
Age	-0.001***	-0.001***	-0.001***	-0.001***
	(0.000)	(0.000)	(0.000)	(0.000)
Hispanic	0.013***	0.010***	0.023***	0.022***
	(0.002)	(0.003)	(0.003)	(0.004)
Black	0.017***	0.015***	0.043***	0.043***
	(0.003)	(0.004)	(0.003)	(0.005)
Asian	0.005*	0.005*	0.006*	0.007*
	(0.002)	(0.002)	(0.003)	(0.003)
Race-Other	-0.001	-0.002	0.005*	0.004
	(0.002)	(0.002)	(0.003)	(0.003)
Race-Unknown	-0.008***	-0.008***	-0.009***	-0.010***
	(0.002)	(0.001)	(0.002)	(0.002)
Preferring Spanish	0.009**	0.009*	0.016***	0.018***
	(0.003)	(0.004)	(0.004)	(0.005)
Social Vulnerability Index (SVI)	-0.000***	-0.000***	-0.000***	-0.000***
	(0.000)	(0.000)	(0.000)	(0.000)
Missing SVI	-0.017***	-0.015***	-0.027***	-0.025***
	(0.002)	(0.002)	(0.003)	(0.002)
COVID-19 Risk Score	-0.000***	-0.000***	-0.000***	-0.000***
	(0.000)	(0.000)	(0.000)	(0.000)
Missing Risk Score	-0.016***	-0.015***	-0.020***	-0.020***
	(0.002)	(0.002)	(0.002)	(0.002)
N	102625	81599	102625	81599
R^2	0.009	0.008	0.018	0.017

Legend: The table reports ordinary least squares (OLS) regressions predicting whether patients scheduled an appointment to get the first dose of the COVID-19 vaccine at UCLA Health within six days of the second reminder date (Columns 1-2) and whether patients obtained the first dose of the COVID-19 vaccine at UCLA Health within four weeks of the second reminder date (Columns 3-4). Columns 1 and 3 include all patients enrolled in the second RCT by February 23, 2021, regardless of whether they fit the inclusion/exclusion criteria for the analysis sample. Columns 2 and 4 exclude patients who were in the *Holdout* arm of the first RCT. The primary predictor, *Follow-Through Reminder*, is a dummy variable coded as 1 when participants received one of the text reminders in the second RCT. Its p-value is < 0.001 in Columns 1-4. Control variables include an indicator for whether a patient was female ("Gender-Female"), an indicator for whether a patient's gender was either other or unknown ("Gender-Other"), patient age in years ("Age"), indicators for whether a patient was Hispanic/Latino ("Hispanic"), Black ("Black"), or Asian ("Asian"), an indicator for whether a patient's race was either American Indian/Alaska Native, Native Hawaiian/Pacific Islander, or other race ("Race-Other"), an indicator for whether a patient's race was unknown ("Race-Unknown"), an indicator for whether a patient's preferred language was Spanish ("Preferring Spanish"), patient social vulnerability index ("Social Vulnerability Index (SVI)"), an indicator for whether a patient had a missing value for social vulnerability index ("Missing SVI"), patient COVID-19 risk score ("COVID-19 Risk Score"), an indicator for whether a patient had a missing value for COVID-19 risk score ("Missing Risk Score"), and batch fixed effects. "Black", "Asian", and "Race-Other" were coded as 0 if a patient was Hispanic or Latino. Robust standard errors are reported in parentheses. *p<0.05; **p<0.01; ***p<0.001 (all two-sided).

Table 18: Effects of Receiving a Text Reminder and Adding Ownership Language (Without Controls)

Dependent Measure	Appointment at UCLA (Within Six Days)		Vaccinated at UCLA (Within Four Weeks)	
	(1)	(2)	(3)	(4)
Follow-Through Reminder	0.062***		0.037***	
	(0.002)		(0.003)	
Ownership		0.016***		0.012***
		(0.002)		(0.003)
Video		-0.002		-0.001
		(0.002)		(0.003)
N	93354	74605	93354	74605
R^2	0.006	0.001	0.002	0.000
Conditions Included	All Conditions	Conditions within the Follow-Through Reminder arm	All Conditions	Conditions within the Follow-Through Reminder arm

Legend: The table reports ordinary least squares (OLS) regressions predicting whether patients scheduled an appointment to get the first dose of the COVID-19 vaccine at UCLA Health within six days of the first reminder date (Columns 1-2) and whether patients obtained the first dose of the COVID-19 vaccine at UCLA Health within four weeks of the first reminder date (Columns 3-4). *Follow-Through Reminder* is a dummy variable coded as 1 when participants received one of the text reminders in the first RCT. Its p-value is < 0.001 in Columns 1 and 3. *Ownership* is a dummy variable coded as 1 when participants received one of the messages containing the ownership language in the first RCT. Its p-value is < 0.001 in Columns 2 and 4 without multiple comparison adjustment and becomes < 0.002 with a Holm-Bonferroni correction in Columns 2 and 4. *Video* is a dummy variable coded as 1 when participants received one of the messages containing a link to the video in the first RCT. Its p-value is 0.322 and 0.609 in Columns 2 and 4, respectively. No control variables are included. *p<0.05; **p<0.01; ***p<0.001 (all two-sided).

Table 19: Effects of Receiving the Second Text Reminder (Without Controls)

Dependent Measure	Appointment at UCLA (Within Six Days)		Vaccinated at UCLA (Within Four Weeks)	
Sample	All Patients	Excl. Patients in the Holdout Arm of the first RCT	All Patients	Excl. Patients in the Holdout Arm of the first RCT
	(1)	(2)	(3)	(4)
Follow-Through Text Reminder	0.016***	0.013***	0.010***	0.010**
	(0.002)	(0.002)	(0.003)	(0.003)
N	67092	53007	67092	53007
R^2	0.001	0.001	0.000	0.000

Legend: The table reports ordinary least squares (OLS) regressions predicting whether patients scheduled an appointment to get the first dose of the COVID-19 vaccine at UCLA Health within six days of the second reminder date (Columns 1-2) and whether patients obtained the first dose of the COVID-19 vaccine at UCLA Health within four weeks of the second reminder date (Columns 3-4). Columns 1 and 3 include all patients in the analysis sample of the second RCT. Columns 2 and 4 exclude patients who were in the *Holdout* arm of the first RCT. The primary predictor, *Follow-Through Reminder*, is a dummy variable coded as 1 when participants received one of the text reminders in the second RCT. Its p-value is < 0.001 in Columns 1-3 and 0.001 in Column 4. No control variables are included. Robust standard errors are reported in parentheses. *p<0.05; **p<0.01; ***p<0.001 (all two-sided).

Table 20: Effects of Receiving a Text Reminder and Adding Ownership Language (Logistic Regressions)

Dependent Measure	Appointment at UCLA (Within Six Days)		Vaccinated at UCLA (Within Four Weeks)	
	(1)	(2)	(3)	(4)
Follow-Through Reminder	0.704***		0.285***	
	(0.031)		(0.024)	
Ownership		0.137***		0.080***
		(0.022)		(0.020)
Video		-0.024		-0.012
		(0.022)		(0.020)
Gender-Female	-0.053*	-0.065**	-0.079***	-0.084***
	(0.021)	(0.022)	(0.018)	(0.020)
Gender-Other	-0.454	-0.368	-0.728	-0.591
	(1.041)	(1.048)	(1.038)	(1.048)
Age	-0.023***	-0.024***	-0.023***	-0.024***
	(0.001)	(0.001)	(0.001)	(0.001)
Hispanic	0.120**	0.112**	0.188***	0.176***
	(0.038)	(0.041)	(0.033)	(0.037)
Black	0.337***	0.353***	0.439***	0.440***
	(0.043)	(0.046)	(0.037)	(0.041)
Asian	0.130***	0.109**	0.133***	0.127***
	(0.037)	(0.040)	(0.033)	(0.036)
Race-Other	-0.160***	-0.146***	-0.103**	-0.127**
	(0.041)	(0.044)	(0.035)	(0.039)
Race-Unknown	-0.489***	-0.452***	-0.467***	-0.467***
	(0.037)	(0.039)	(0.032)	(0.035)
Preferring Spanish	0.092	0.074	0.185***	0.190***
	(0.057)	(0.061)	(0.047)	(0.052)
Social Vulnerability Index (SVI)	-0.009***	-0.008***	-0.008***	-0.008***
	(0.000)	(0.000)	(0.000)	(0.000)
Missing SVI	-0.874***	-0.888***	-1.057***	-1.048***
	(0.056)	(0.059)	(0.051)	(0.055)
COVID-19 Risk Score	0.001*	0.001**	0.001***	0.001***
	(0.000)	(0.000)	(0.000)	(0.000)
Missing Risk Score	-0.714***	-0.694***	-0.725***	-0.716***
	(0.045)	(0.047)	(0.039)	(0.043)
N	93354	74605	93354	74605
R^2				
Conditions Included	All Conditions	Conditions within the Follow-Through Reminder arm	All Conditions	Conditions within the Follow-Through Reminder arm

Legend: The table reports logistic regressions predicting whether patients scheduled an appointment to get the first dose of the COVID-19 vaccine at UCLA Health within six days of the first reminder date (Columns 1-2) and whether patients obtained the first dose of the COVID-19 vaccine at UCLA Health within four weeks of the first reminder date (Columns 3-4). *Follow-Through Reminder* is a dummy variable coded as 1 when participants received one of the text reminders in the first RCT. Its p-value is $<$ 0.001 in Columns 1 and 3. *Ownership* is a dummy variable coded as 1 when participants received one of the messages containing the ownership language in the first RCT. Its p-value is $<$ 0.001 in Columns 2 and 4 without multiple comparison adjustment and becomes $<$ 0.002 with a Holm-Bonferroni correction in Columns 2 and 4. *Video* is a dummy variable coded as 1 when participants received one of the messages containing a link to the video in the first RCT. Its p-value is 0.273 and 0.529 in Columns 2 and 4, respectively. Control variables include an indicator for whether a patient was female ("Gender-Female"), an indicator for whether a patient's gender was either other or unknown ("Gender-Other"), patient age in years ("Age"), indicators for whether a patient was Hispanic/Latino ("Hispanic"), Black ("Black"), or Asian ("Asian"), an indicator for whether a patient's race was either American Indian/Alaska Native, Native Hawaiian/Pacific Islander, or other race ("Race-Other"), an indicator for whether a patient's race was unknown ("Race-Unknown"), an indicator for whether a patient's preferred language was Spanish ("Preferring Spanish"), patient social vulnerability index ("Social Vulnerability Index (SVI)"), an indicator for whether a patient had a missing value for social vulnerability index ("Missing SVI"), patient COVID-19 risk score ("COVID-19 Risk Score"), an indicator for whether a patient had a missing value for COVID-19 risk score ("Missing Risk Score"), and batch fixed effects. "Black", "Asian", and "Race-Other" were coded as 0 if a patient was Hispanic or Latino. Robust standard errors are reported in parentheses. $^*p<0.05$; $^{**}p<0.01$; $^{***}p<0.001$ (all two-sided).

Table 21: Effects of Receiving the Second Text Reminder (Logistic Regressions)

Dependent Measure	Appointment at UCLA (Within Six Days)		Vaccinated at UCLA (Within Four Weeks)	
Sample	All Patients	Excl. Patients in the Holdout Arm of the first RCT	All Patients	Excl. Patients in the Holdout Arm of the first RCT
	(1)	(2)	(3)	(4)
Follow-Through Reminder	0.448***	0.392***	0.172***	0.172**
	(0.062)	(0.072)	(0.046)	(0.053)
Gender-Female	-0.049	-0.032	-0.066*	-0.063
	(0.038)	(0.044)	(0.031)	(0.035)
Gender-Other	0.000	0.000	0.000	0.000
	(.)	(.)	(.)	(.)
Age	-0.016***	-0.015***	-0.019***	-0.016***
	(0.002)	(0.003)	(0.002)	(0.002)
Hispanic	0.329***	0.284***	0.328***	0.320***
	(0.067)	(0.079)	(0.055)	(0.063)
Black	0.367***	0.324***	0.518***	0.521***
	(0.077)	(0.091)	(0.060)	(0.069)
Asian	0.157*	0.162*	0.180**	0.204**
	(0.069)	(0.080)	(0.056)	(0.064)
Race-Other	-0.020	-0.064	0.076	0.049
	(0.073)	(0.086)	(0.058)	(0.067)
Race-Unknown	-0.348***	-0.417***	-0.329***	-0.369***
	(0.065)	(0.078)	(0.054)	(0.062)
Preferring Spanish	0.175	0.194	0.240***	0.298***
	(0.091)	(0.107)	(0.073)	(0.083)
Social Vulnerability Index (SVI)	-0.008***	-0.008***	-0.007***	-0.007***
	(0.001)	(0.001)	(0.001)	(0.001)
Missing SVI	-1.097***	-1.073***	-1.245***	-1.188***
	(0.110)	(0.127)	(0.095)	(0.107)
COVID-19 Risk Score	-0.001	-0.001	-0.002**	-0.002**
	(0.001)	(0.001)	(0.001)	(0.001)
Missing Risk Score	-0.664***	-0.648***	-0.702***	-0.708***
	(0.082)	(0.096)	(0.072)	(0.083)
N	67068	52989	67068	52989
R^2				

Legend: The table reports logistic regressions predicting whether patients scheduled an appointment to get the first dose of the COVID-19 vaccine at UCLA Health within six days of the second reminder date (Columns 1-2) and whether patients obtained the first dose of the COVID-19 vaccine at UCLA Health within four weeks of the second reminder date (Columns 3-4). Columns 1 and 3 include all patients in the analysis sample of the second RCT. Columns 2 and 4 exclude patients who were in the *Holdout* arm of the first RCT. The primary predictor, *Follow-Through Reminder*, is a dummy variable coded as 1 when participants received one of the text reminders in the second RCT. Its p-value is < 0.001 in Columns 1-3 and 0.001 in Column 4. Control variables include an indicator for whether a patient was female (“Gender-Female”), an indicator for whether a patient’s gender was either other or unknown (“Gender-Other”), patient age in years (“Age”), indicators for whether a patient was Hispanic/Latino (“Hispanic”), Black (“Black”), or Asian (“Asian”), an indicator for whether a patient’s race was either American Indian/Alaska Native, Native Hawaiian/Pacific Islander, or other race (“Race-Other”), an indicator for whether a patient’s race was unknown (“Race-Unknown”), an indicator for whether a patient’s preferred language was Spanish (“Preferring Spanish”), patient social vulnerability index (“Social Vulnerability Index (SVI)”), an indicator for whether a patient had a missing value for social vulnerability index (“Missing SVI”), patient COVID-19 risk score (“COVID-19 Risk Score”), an indicator for whether a patient had a missing value for COVID-19 risk score (“Missing Risk Score”), and batch fixed effects. “Black”, “Asian”, and “Race-Other” were coded as 0 if a patient was Hispanic or Latino. Robust standard errors are reported in parentheses. $^{*}p<0.05$; $^{**}p<0.01$; $^{***}p<0.001$ (all two-sided).

Table 22: Effects of Receiving a Text Reminder and Adding Ownership Language on Appointments at UCLA Health and Vaccinations Anywhere Within Four Weeks

Dependent Measure	Appointment at UCLA (Within Four Weeks)		Vaccinated Anywhere (Within Four Weeks)	
	(1)	(2)	(3)	(4)
Follow-Through Reminder	0.037***		0.021***	
	(0.003)		(0.004)	
Ownership		0.009**		0.009*
		(0.003)		(0.003)
Video		-0.004		-0.002
		(0.003)		(0.003)
Gender-Female	-0.004	-0.006*	-0.004	-0.006
	(0.003)	(0.003)	(0.003)	(0.003)
Gender-Other	-0.052	-0.064	-0.123**	-0.113
	(0.029)	(0.038)	(0.046)	(0.061)
Age	-0.004***	-0.004***	-0.002***	-0.002***
	(0.000)	(0.000)	(0.000)	(0.000)
Hispanic	0.035***	0.033***	0.020***	0.020**
	(0.005)	(0.006)	(0.006)	(0.007)
Black	0.087***	0.089***	0.013	0.014
	(0.007)	(0.007)	(0.007)	(0.008)
Asian	0.027***	0.027***	0.012*	0.010
	(0.005)	(0.006)	(0.006)	(0.007)
Race-Other	-0.009	-0.012*	-0.036***	-0.039***
	(0.005)	(0.006)	(0.006)	(0.006)
Race-Unknown	-0.055***	-0.056***	-0.018***	-0.024***
	(0.003)	(0.004)	(0.004)	(0.005)
Preferring Spanish	0.028***	0.027***	0.003	0.007
	(0.007)	(0.008)	(0.008)	(0.009)
Social Vulnerability Index (SVI)	-0.001***	-0.001***	-0.001***	-0.001***
	(0.000)	(0.000)	(0.000)	(0.000)
Missing SVI	-0.110***	-0.114***	-0.205***	-0.206***
	(0.004)	(0.004)	(0.004)	(0.005)
COVID-19 Risk Score	-0.000	-0.000	0.000*	0.000*
	(0.000)	(0.000)	(0.000)	(0.000)
Missing Risk Score	-0.087***	-0.088***	-0.079***	-0.079***
	(0.004)	(0.005)	(0.005)	(0.006)
N	93354	74605	93354	74605
R^2	0.061	0.060	0.037	0.037
Conditions Included	All Conditions	Conditions within the Follow-Through Reminder arm	All Conditions	Conditions within the Follow-Through Reminder arm

Legend: The table reports ordinary least squares (OLS) regressions predicting whether patients scheduled an appointment to get the first dose of the COVID-19 vaccine at UCLA Health within four weeks of the first reminder date (Columns 1-2) and whether patients obtained the first dose of the COVID-19 vaccine anywhere within four weeks of the first reminder date (Columns 3-4).
Follow-Through Reminder is a dummy variable coded as 1 when participants received one of the text reminders in the first RCT. Its p-value is < 0.001 in Columns 1 and 3. *Ownership* is a dummy variable coded as 1 when participants received one of the messages containing the ownership language in the first RCT. Its p-value is 0.001 in Column 2 and 0.010 in Column 4 without multiple comparison adjustment; and its p-value becomes 0.002 in Column 2 and 0.020 in Column 4 with a Holm-Bonferroni correction. *Video* is a dummy variable coded as 1 when participants received one of the messages containing a link to the video in the first RCT. Its p-value is 0.197 and 0.604 in Columns 2 and 4, respectively. Control variables include an indicator for whether a patient was female ("Gender-Female"), an indicator for whether a patient's gender was either other or unknown ("Gender-Other"), patient age in years ("Age"), indicators for whether a patient was Hispanic/Latino ("Hispanic"), Black ("Black"), or Asian ("Asian"), an indicator for whether a patient's race was either American Indian/Alaska Native, Native Hawaiian/Pacific Islander, or other race ("Race-Other"), an indicator for whether a patient's race was unknown ("Race-Unknown"), an indicator for whether a patient's preferred language was Spanish ("Preferring Spanish"), patient social vulnerability index ("Social Vulnerability Index (SVI)"), an indicator for whether a patient had a missing value for social vulnerability index ("Missing SVI"), patient COVID-19 risk score ("COVID-19 Risk Score"), an indicator for whether a patient had a missing value for COVID-19 risk score ("Missing Risk Score"), and batch fixed effects. "Black", "Asian", and "Race-Other" were coded as 0 if a patient was Hispanic or Latino. Robust standard errors are reported in parentheses. $^{*}p<0.05$; $^{**}p<0.01$; $^{***}p<0.001$ (all two-sided).

Table 23: Effects of Receiving the Second Text Reminder on Appointments at UCLA Health and Vaccinations Anywhere Within Four Weeks

Dependent Measure	Appointment at UCLA (Within Four Weeks)		Vaccinated Anywhere (Within Four Weeks)	
Sample	All Patients	Excl. Patients in the Holdout Arm of the First RCT	All Patients	Excl. Patients in the Holdout Arm of the First RCT
	(1)	(2)	(3)	(4)
Follow-Through Reminder	0.011***	0.012***	0.007	0.006
	(0.003)	(0.003)	(0.004)	(0.005)
Gender-Female	0.003	0.002	-0.002	-0.003
	(0.002)	(0.003)	(0.003)	(0.004)
Gender-Other	-0.037***	-0.040***	-0.042	-0.013
	(0.006)	(0.007)	(0.052)	(0.068)
Age	-0.002***	-0.002***	-0.001***	-0.001***
	(0.000)	(0.000)	(0.000)	(0.000)
Hispanic	0.033***	0.029***	0.027***	0.025***
	(0.005)	(0.005)	(0.006)	(0.007)
Black	0.059***	0.059***	0.012	0.015
	(0.006)	(0.007)	(0.007)	(0.008)
Asian	0.016***	0.017***	0.006	0.005
	(0.005)	(0.005)	(0.006)	(0.007)
Race-Other	0.010*	0.008	-0.021***	-0.021***
	(0.004)	(0.005)	(0.006)	(0.006)
Race-Unknown	-0.022***	-0.022***	0.011*	0.007
	(0.003)	(0.003)	(0.005)	(0.005)
Preferring Spanish	0.024***	0.025***	0.011	0.014
	(0.007)	(0.008)	(0.009)	(0.010)
Social Vulnerability Index (SVI)	-0.001***	-0.001***	0.000	0.000
	(0.000)	(0.000)	(0.000)	(0.000)
Missing SVI	-0.060***	-0.056***	-0.140***	-0.138***
	(0.003)	(0.003)	(0.004)	(0.004)
COVID-19 Risk Score	-0.000***	-0.000***	0.000	0.000
	(0.000)	(0.000)	(0.000)	(0.000)
Missing Risk Score	-0.041***	-0.041***	-0.037***	-0.034***
	(0.003)	(0.004)	(0.005)	(0.006)
N	67092	53007	67092	53007
R^2	0.029	0.027	0.018	0.018

Legend: The table reports ordinary least squares (OLS) regressions predicting whether patients scheduled an appointment to get the first dose of the COVID-19 vaccine at UCLA Health within four weeks of the second reminder date (Columns 1-2) and whether patients obtained the first dose of the COVID-19 vaccine anywhere within four weeks of the second reminder date (Columns 3-4). Columns 1 and 3 include all patients in the analysis sample of the second RCT. Columns 2 and 4 exclude patients who were in the *Holdout* arm of the first RCT. The primary predictor, *Follow-Through Reminder*, is a dummy variable coded as 1 when participants received one of the text reminders in the second RCT. Its p-value is < 0.001 in Columns 1-2, 0.127 in Column 3, and 0.219 in Column 4. Control variables include an indicator for whether a patient was female ("Gender-Female"), an indicator for whether a patient's gender was either other or unknown ("Gender-Other"), patient age in years ("Age"), indicators for whether a patient was Hispanic/Latino ("Hispanic"), Black ("Black"), or Asian ("Asian"), an indicator for whether a patient's race was either American Indian/Alaska Native, Native Hawaiian/Pacific Islander, or other race ("Race-Other"), an indicator for whether a patient's race was unknown ("Race-Unknown"), an indicator for whether a patient's preferred language was Spanish ("Preferring Spanish"), patient social vulnerability index ("Social Vulnerability Index (SVI)"), an indicator for whether a patient had a missing value for social vulnerability index ("Missing SVI"), patient COVID-19 risk score ("COVID-19 Risk Score"), an indicator for whether a patient had a missing value for COVID-19 risk score ("Missing Risk Score"), and batch fixed effects. "Black", "Asian", and "Race-Other" were coded as 0 if a patient was Hispanic or Latino. Robust standard errors are reported in parentheses. $^{*}p<0.05$; $^{**}p<0.01$; $^{***}p<0.001$ (all two-sided).

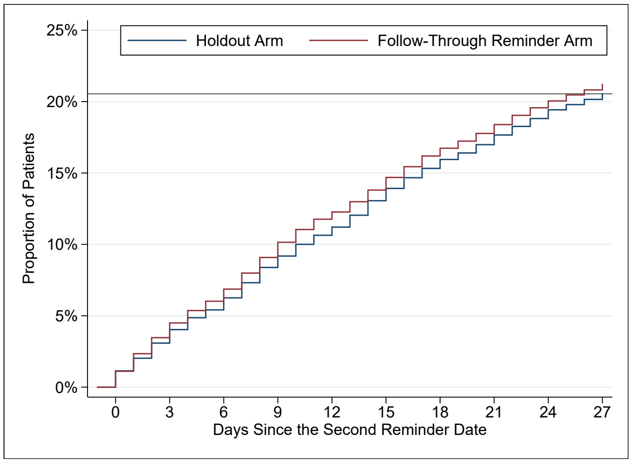

Figure 3: Kaplan-Meier Curves Reflecting the Proportion of Patients Who Had Obtained the First Dose Anywhere by a Given Day After the Second Reminder Date

Legend: This figure depicts the Kaplan-Meier curves tracking the percentage of patients in the *Holdout* arm (blue; N = 9,625) versus the *Follow-Through Reminder* arm (red; N = 61,237) of the second RCT who had received the first dose of COVID-19 vaccine at UCLA Health by a given day during a four-week period from the second reminder date (0 on the x-axis) to 27 days later. The solid horizontal line represents the proportion of patients who had received the first dose by the end of 27 days after the second reminder date (i.e., 20.6%).

Table 24: Effects of Receiving the Second Text Reminder on Vaccinations Anywhere Within Two Weeks

Dependent Measure	Vaccinated Anywhere (Within Two Weeks)	
Sample	All Patients	Excl. Patients in the Holdout Arm of the First RCT
	(1)	(2)
Follow-Through Reminder	0.010**	0.008+
	(0.004)	(0.004)
Gender-Female	-0.003	-0.003
	(0.003)	(0.003)
Gender-Other	-0.082***	-0.078***
	(0.007)	(0.009)
Age	-0.000*	-0.000
	(0.000)	(0.000)
Hispanic	0.015**	0.011+
	(0.005)	(0.006)
Black	0.001	0.000
	(0.006)	(0.007)
Asian	0.004	0.004
	(0.005)	(0.006)
Race-Other	-0.016***	-0.021***
	(0.005)	(0.005)
Race-Unknown	0.010**	0.005
	(0.004)	(0.004)
Preferring Spanish	-0.005	-0.002
	(0.007)	(0.008)
Social Vulnerability Index (SVI)	-0.000	-0.000
	(0.000)	(0.000)
Missing SVI	-0.090***	-0.088***
	(0.003)	(0.003)
COVID-19 Risk Score	0.000**	0.000*
	(0.000)	(0.000)
Missing Risk Score	-0.027***	-0.024***
	(0.004)	(0.005)
N	67092	53007
R^2	0.010	0.010

Legend: The table reports ordinary least squares (OLS) regressions predicting whether patients obtained the first dose of the COVID-19 vaccine anywhere within two weeks of the second reminder date. Column 1 includes all patients in the analysis sample of the second RCT. Column 2 excludes patients who were in the *Holdout* arm of the first RCT. The primary predictor, *Follow-Through Reminder*, is a dummy variable coded as 1 when participants received one of the text reminders in the second RCT. Its p-value is 0.008 in Column 1 and 0.057 in Column 2. Control variables include an indicator for whether a patient was female ("Gender-Female"), an indicator for whether a patient's gender was either other or unknown ("Gender-Other"), patient age in years ("Age"), indicators for whether a patient was Hispanic/Latino ("Hispanic"), Black ("Black"), or Asian ("Asian"), an indicator for whether a patient's race was either American Indian/Alaska Native, Native Hawaiian/Pacific Islander, or other race ("Race-Other"), an indicator for whether a patient's race was unknown ("Race-Unknown"), an indicator for whether a patient's preferred language was Spanish ("Preferring Spanish"), patient social vulnerability index ("Social Vulnerability Index (SVI)"), an indicator for whether a patient had a missing value for social vulnerability index ("Missing SVI"), patient COVID-19 risk score ("COVID-19 Risk Score"), an indicator for whether a patient had a missing value for COVID-19 risk score ("Missing Risk Score"), and batch fixed effects. "Black", "Asian", and "Race-Other" were coded as 0 if a patient was Hispanic or Latino. Robust standard errors are reported in parentheses. +p<0.10; *p<0.05; **p<0.01; ***p<0.001 (all two-sided).

1.6 Additional Information About UCLA Health Patients and Vaccination Patterns

In this section, we present additional information about UCLA Health patients' characteristics and vaccination behavior that may be of interest to policymakers and the public.

1.6.1 Representativeness of Our Study Sample

To understand the extent to which our sample is representative, Extended Data Table 7 summarizes demographics and first-dose vaccination rates of (1) California residents, (2) Los Angeles (LA) County residents, (3) all UCLA Health primary care and specialty care patients (about 600,000 in total as of May 25, 2021) regardless of whether they were invited to get vaccinated at UCLA Health, (4) patients enrolled in the first RCT by February 23, 2021 (N=132,337), and (5) patients enrolled in the first RCT by February 23, 2021 and were further excluded from analyses based on our exclusion criteria (N=93,354). We focus on first-dose vaccination rates of people at or above 65 years old because this age group was eligible for COVID-19 vaccines in January and February, 2021, and was thus comparable between California, LA County, and our study sample.

We highlight three observations. First, the population of all UCLA Health primary and specialty care patients (Column 3) has a higher percentage of senior patients, a lower percentage of Hispanic/Latino patients, and a similar percentage of females and White patients (if we do not count patients whose race was unknown) than LA County residents (Column 2) and California residents (Column 1). As of February 23, 2021, first-dose vaccination rates of seniors were higher among the overall UCLA Health patient population than among LA County and California residents by 7-13 percentage points. As of March 23, 2021, seniors' vaccination rates were virtually the same between UCLA Health and LA County, both being higher than rates in California by 5 percentage points. As of April 23, 2021, seniors' vaccination rates at UCLA Health were very similar to rates in California, both being 6-7 percentage points lower than rates in LA County. Altogether, we think UCLA Health's overall patient population was reasonably similar to the general LA County or California population in their likelihood of getting the COVID-19 vaccine, at least among seniors.

Second, demographic composition was similar between patients enrolled in the first RCT by February 23, 2021 (Column 4) and the UCLA Health primary and specialty care patient population (Column 3), except that the former contained a much larger proportion of seniors because seniors were eligible for the COVID-19 vaccine early on. Seniors' vaccination rates were lower among patients enrolled in the first RCT by February 23, 2021 than among the overall UCLA Health primary and specialty care patient population. This is expected because if patients had obtained the first dose before their turn to be invited to get vaccinated at UCLA Health, they were not invited in the first place. Further, among those who were invited, patients who scheduled or obtained the first dose before the first reminder date were not enrolled in the first RCT. In other words, due to UCLA Health's invitation process and our RCT's enrollment process, patients enrolled in the first RCT were more hesitant to get vaccinated than the overall UCLA Health patient population.

Third, demographic composition was similar between patients included in our analysis of the first RCT (after applying exclusion criteria; Column 5) and patients enrolled in the first RCT by February 23, 2021 (Column 4), but seniors' vaccination rates were lower in the former group. This is also expected because, as pre-registered, we excluded from our analysis patients who were enrolled in the first RCT but scheduled the first-dose appointment at UCLA Health by 3pm PST on the first reminder date or obtained the first

dose anywhere before the first reminder date based on the latest vaccination records we could obtain. Thus, patients in the analysis sample of the first RCT were even more hesitant to get vaccinated than patients enrolled in the first RCT by February 23, 2021.

To summarize, UCLA Health patients in general have comparable vaccination rates with LA and California residents. However, because of how UCLA Health patients were invited to get the vaccine, subsequently enrolled in our RCTs, and finally included in our analysis, patients in the analysis sample of our RCTs were more hesitant about getting the COVID-19 vaccine than average residents of LA county or California. We believe our finding—that behaviorally informed nudges can improve vaccine uptake among patients who are more hesitant than average to get vaccinated—is valuable because a large portion of people in the U.S. and worldwide feel uncertain about getting vaccinated and could benefit from interventions like ours.

1.6.2 Cancellation and No-Show Rates

Among patients in our first RCT sample who scheduled the first-dose appointment at UCLA Health within six days of the first reminder date (n=10,106), 89.28% either had obtained their first dose or had an active, upcoming appointment as of May 25, 2021. That is, only 10.72% patients cancelled their appointments without rescheduling a new one by May 25, 2021. Among these patients, 8,267 had an active first-dose appointment that was supposed to take place before May 25, 2021 (n=8,267), and 99.75% of them completed it; that is, only 21 patients did not show up for their active first-dose appointments, indicating a 0.25% no-show rate. The low no-show rate may occur because UCLA Health exerted extra effort to remind people of their vaccination appointment or because patients who scheduled an appointment were determined to receive the vaccine. The relatively low cancellation rate and the extremely low no-show rate suggest that the biggest challenge to tackle for the vaccine uptake challenge is to get people to schedule an appointment.

1.6.3 Scheduling Rates of the Second Dose

Among people who were in the analysis sample of either RCT and obtained the first dose of Moderna or Pfizer at UCLA Health as of our data extraction on May 25, 2021, 87.40% had an appointment for the second dose or already obtained the second dose as of May 25, 2021. Since we do not have data about appointments made outside of UCLA Health, we could only report statistics based on UCLA Health records. UCLA Health took great measures to ensure that patients schedule their second-dose appointment right after they obtain the first dose, which helps combat barriers patients may have otherwise faced (e.g., forgetfulness, procrastination) if they were left to schedule the second-dose appointment later online. The high appointment rate for the second dose may also be driven by patients' strong intentions to get fully vaccinated among those who cared enough to get the first dose.

2 Online Experiments Assessing Intentions and Mechanisms - February 2021

We conducted two pre-registered online experiments in February 2021—the same time period as when the analysis sample of our RCTs was enrolled into the RCTs. We had three objectives. First, using a different sample of respondents than patients at UCLA Health, we sought to examine how the text messages from the first RCT affect (1) people's reported intentions to get the vaccine, measured via eliciting the likelihood of scheduling the COVID-19 vaccine, and (2) people's perceptions of these messages' persuasiveness. Second, we wanted to check if the ownership language deployed in the first RCT indeed creates a sense of psychological ownership towards the vaccine, as we intended. Third, we explored whether the video shifts certain beliefs and perceptions about COVID-19 and the COVID-19 vaccine. We pre-registered the experiments on aspredicted.org (https://aspredicted.org/blind.php?x=u2ng5c and https://aspredicted.org/blind.php?x=ae3ci5). The two experiments had the same design and analysis plan but the second experiment had a different recruitment method. The key results are similar between the two experiments (see Supplementary Tables 29-33), so we combined the data, which gave us a larger sample for analyzing heterogeneous treatment effects.

2.1 Method

2.1.1 Participants

In the first online experiment, we aimed to recruit 1,200 participants who fit our pre-registered selection criteria. People on Amazon's Mechanical Turk (MTurk) and Prolific Academic (Prolific) who passed a Captcha and an attention check question, and had not already received the COVID-19 vaccine or did not already have a COVID-19 vaccination appointment were eligible to take our study. To identify participants eligible for taking the study, we first asked participants several screening questions. Only those who satisfied the aforementioned criteria were allowed to take our study; those who were not eligible for the study were offered a payment of $0.10 as a compensation for their time. Possibly because of this recruitment method and our attempt to get a balanced sample of Democrats and Republicans (see the next paragraph), our initial data collection on MTurk became very slow after we hit about 500 Democrats and 450 Republicans, at which point we switched to Prolific to speed up data collection. This is a minor deviation from our pre-registration. On Prolific, we initially used the same recruitment method as on MTurk but later were informed that we could not kick people out of a study on Prolific even if we compensated them for the time they spent taking a few screening questions. Thus, for about 40 participants recruited towards the end of the first experiment (as well as all participants taking our second experiment on Prolific), we did not kick people out in the survey but only kept those who satisfied our criteria in the analysis.

Since the majority of participants on MTurk and Prolific identify with the Democratic Party based on our prior data collection experiences and prior research[52,53], we had to take extra measures to obtain a more balanced number of Republicans and Democrats as study participants in order to conduct heterogeneity analyses by political affiliation. We were interested in this analysis because national surveys suggest Republicans are more hesitant about getting the COVID-19 vaccine than Democrats[36,41]. The survey we conducted to inform our design of the video also revealed that Republicans had significantly lower self-reported intentions to take the COVID-19 vaccine, compared to Democrats. See Section 5. When collecting data on MTurk, we used preset quotas in our survey on Qualtrics such that once the number of Democrats hit 600, only people

who identified with Republicans could take the study. When we later collected data on Prolific, we used Prolific's screening tools to separately target Republicans and Democrats.

Our sample for the first experiment consists of 1,163 participants (51.59% female, $M_{age} = 39.64$) who satisfied the aforementioned criteria, at least completed the portion of the survey involving our pre-registered primary dependent variables, did not report having technical issues with watching the video, and did not report having taken a similar study on MTurk (only relevant to the Prolific sample). We did not pre-register dropping people who had technical problems with the video or had taken a similar study on MTurk in the first experiment, but we realized these issues later and added these exclusion criteria when pre-registering the second experiment. Here, we drop these people from the first experiment for consistency but keeping these observations does not qualitatively change the results.

In the second online experiment, we aimed to recruit 800 participants from Prolific who fit our pre-registered selection criteria: people who passed an attention check question, had not already received the COVID-19 vaccine or did not already have a COVID-19 vaccination appointment, did not report having taken a similar study on MTurk, and had no technical problems with watching our COVID-19 video. We again used Prolific's screening tools and sought to recruit 400 Republicans and 400 Democrats. Prolific's screening tools are not perfect as some people marked by Prolific as Democrats (Republicans) identified themselves as Republicans (Democrats) or neither Republicans or Democrats when responding to our survey. Our sample for the second experiment consists of 840 participants (50.12% female, $M_{age} = 35.46$) who satisfied the aforementioned criteria and at least completed the portion of the survey involving our pre-registered primary dependent variables.

Altogether, across the two online experiments, our final sample consists of 2,003 participants (50.97% female, $M_{age} = 37.89$). If a person took the survey and completed our pre-registered dependent variables more than once, we kept their first response.

2.1.2 Procedure

Upon passing the screening, we instructed participants to imagine receiving one of the reminders from the first RCT. Specifically, they imagined receiving a text message from their healthcare provider encouraging them to get the COVID-19 vaccine. Precisely, we instructed participants to "imagine that you become eligible to get the COVID-19 vaccine, and your healthcare provider sends you the following message informing you of your eligibility." In our pre-registration, we said, "...we will ask participants to imagine being a hospital patient and receiving a text message about their eligibility to get the COVID vaccine." However, we did not actually ask participants to imagine being a hospital patient. This discrepancy was unintentional. When we drafted the pre-registration, what we had in mind was the language that we actually presented to participants in the experiments. We did not copy the exact language from our survey instrument since the pre-registration questionnaire only asked us to provide some context into the study design rather than presenting the exact scenario participants would see. By "a hospital patient", we meant to use the same loose definition as how UCLA Health thinks about participants involved in our RCTs: all participants in our RCTs are considered UCLA Health patients (and we refer to them as UCLA Health patients in our paper), even though they were not necessarily hospitalized and did not necessarily visit a doctor during our study period. In our online experiments, since participants were asked to imagine receiving a message from their healthcare provider (just like people in our RCTs received a message from UCLA Health), we said in our pre-registration that

they were asked to imagine being a hospital patient. In hindsight, we realized that this discrepancy could cause confusion and thus clarified our intention here.

We randomly assigned participants to one of four conditions: *Basic Reminder*, *Ownership Reminder*, *Basic Reminder with Video*, or *Ownership Reminder with Video* condition. Participants in each condition were presented with the text of the reminder from the corresponding condition of the first RCT , except that we used placeholders for patient first name, video link, and the link to the vaccination appointment website and we replaced "UCLA Health" with "our clinics". Specifically,

- In the *Basic Reminder* condition, participants saw the following message: "[Your first name], you can get the COVID-19 vaccine at our clinics. Make a vaccination appointment here: [link to the appointment website]".
- In the *Ownership Reminder* condition, the message read, "[Your first name], a COVID-19 vaccine has just been made available to you at our clinics. Claim your dose today by making a vaccination appointment here: [link to the appointment website]".
- In the *Basic Reminder with Video* condition, the message read, "[Your first name], you can get the COVID-19 vaccine at our clinics. Please watch this important 2 min video: [link to the video]. Make a vaccination appointment here: [link to the appointment website]".
- In the *Ownership Reminder with Video* condition, the message read, "[Your first name], a COVID-19 vaccine has just been made available to you at our clinics. Please take 2 simple steps: 1. Watch this important 2 min video: [link to the video]. 2. Claim your dose today by making a vaccination appointment here: [link to the appointment websites]".

In the two video conditions, we then asked participants to watch the 2-minute video we sent to patients in the corresponding conditions of the first RCT. The full survey instructions are available at https://osf.io/qn8hr/?view_only=cf7b2bc590054aee8c4a2bae99ef20c5.

2.1.3 Measures

After people read the text message presented to them and watched the video (if they were in one of the video conditions), we collected a number of measures.

Primary Dependent Measures. Our first primary dependent measure was participants' self-reported likelihood of scheduling a COVID-19 vaccination appointment upon receiving the message (*Scheduling Likelihood*): "How likely would you be to schedule a vaccination appointment after receiving this message from your healthcare provider?" (from 1 = Not at all Likely to 7 = Very Likely). Our second dependent measure was participants' perceived persuasiveness of the text message (*Persuasiveness*): "How persuasive do you think the message is?" (from 1 = Not at all persuasive to 7 = Very persuasive).

Mechanism Measures for the Ownership Language. To test whether the ownership language enhanced feelings of psychological ownership towards the vaccine, we measured *Psychological Ownership*[31], "To what extent does the text message make you feel that the COVID-19 vaccine is already yours?" (from 1 = Not at all to 7 = Very much). To address the possibility that our messages containing the ownership language are longer and thus could make getting the COVID-19 vaccine seem more difficult, we measured

Easiness, "To what extent does the text message make you feel that getting the COVID-19 vaccine is easy?" (from 1 = Not at all to 7 = Very much).

Mechanism Measures for the Video. To test how the video affected participants' beliefs and perceptions about COVID-19 and the COVID-19 vaccine, we collected the following measures:

- *Infection Likelihood without Vaccine*: We asked participants to report their likelihood of getting infected with COVID-19 (including cases with and without symptoms) if they did not get the COVID-19 vaccine (from 0% = I certainly won't get infected with COVID-19 to 100% = I certainly will get infected with COVID-19). We also asked participants to report their likelihood of developing symptomatic COVID-19 if they did not get the COVID-19 vaccine (from 0% = I certainly won't develop it to 100% = I certainly will develop it). Responses to those questions were highly correlated ($r = 0.82$, $p < 0.0001$) and thus were averaged to form a measure of *Infection Likelihood without Vaccine.*

- *Infection Likelihood with Vaccine*: We asked participants to report their likelihood of getting infected with COVID-19 (including cases with and without symptoms) if they got the COVID-19 vaccine (from 0% = I certainly won't get infected with COVID-19 to 100% = I certainly will get infected with COVID-19). We also asked participants to report their likelihood of developing symptomatic COVID-19 if they got the COVID-19 vaccine (from 0% = I certainly won't develop it to 100% = I certainly will develop it). Responses to those questions were highly correlated ($r = 0.71$, $p < 0.0001$) and were averaged to form a measure of *Infection Likelihood with Vaccine.* The two questions about the likelihood of developing general COVID-19 infections (with vs. without the vaccine) and the two questions about developing symptomatic COVID-19 (with vs. without the vaccine) were counterbalanced. The results reported later are robust if we separately examine people's beliefs about general COVID-19 infections versus symptomatic COVID-19.

- *Vaccine Effectiveness*: For each participant, we subtracted *Infection Likelihood with Vaccine* from *Infection Likelihood without Vaccine* to capture her belief in the effectiveness of the COVID-19 vaccine. The value of this variable in theory could range from -100% to 100%, and a positive value indicates that participants believed getting the vaccine could reduce their infection likelihood. In our analysis, for *Infection Likelihood without Vaccine*, *Infection Likelihood with Vaccine*, and *Vaccine Effectiveness*, we multiplied the raw data by 100.

- *Worry of Transmission*: Participants indicated the extent to which they worried about passing COVID-19 to other people (from 1 = Not at all to 7 = Very much).

- *Anticipated Regret*: Participants indicated how much they would regret not getting the COVID-19 vaccine if, when eligible, they decided not to get it and ended up getting symptomatic COVID-19 (from 1 = Not at all to 7 = Very much).

- *Trust in Vaccine*: We measured trust in the safety of the vaccine and the vaccine development process with five questions on a seven-point scale (from 1 = Not at all to 7 = Very Much). The questions assessed worries about side effects ("To what extent are you concerned about potential side effects of the COVID-19 vaccine?"), concerns about potential negative long-term impacts of the vaccine ("To what extent do you worry that the COVID-19 vaccine will have negative long-term impacts on your health?"), concerns about the speed of the vaccine authorization process ("To what extent are you

concerned that the current COVID-19 vaccines were authorized for use too quickly?"), overall trust in the development of the vaccine ("How much do you trust the research and development process of COVID-19 vaccines?"), and perceived safety of the vaccine ("How confident are you that the research and development process of COVID-19 vaccine has produced a safe vaccine?"). Participants' responses to these five questions reached a high inter-item reliability (Cronbach's alpha = 0.93); thus, we averaged responses to these questions (after reverse coding the first three questions) to form a composite score of *Trust in Vaccine*. The higher one's composite score is, the more they trusted the safety of vaccine and its development process.

Additional Measures. In the conditions containing the video, we also measured the perceived persuasiveness ("How persuasive do you think the video is?") and the perceived accuracy of the video ("How accurate do you think the video is?"; from a 1 = Not at all to 7 = Very much). Finally, participants reported whether they got the flu shot during each of the two recent flu seasons (2019-2020 and 2020-2021 seasons), the frequency with which they washed their hands when coming back from being outside (from 1 = Never or almost never to 5 = Always or almost always), and the frequency with which they wore a mask when they could not maintain social distance outside (from 1 = Never or almost never to 5 = Always or almost always), whether they ever tested positive for COVID-19 (or thought they had COVID-19), their demographics and socio-economics status (age, gender, race/ethnicity, highest level of education attained, pre-tax household income in 2020, whether they had children under 18, whether they were living with anyone in the same household, and which state they were living in). The question about children was added after our first day of data collection because we learned from a colleague's presentation that people with children reported lower COVID-19 vaccination intentions than those without children. We decided to add this for exploratory heterogeneity analyses. Participants in the video conditions additionally reported whether they had any technical issue with the video. Participants recruited from Prolific were also asked whether they took a similar study on MTurk.

2.2 Results

As pre-registered, we used ordinary least squares (OLS) regressions with robust standard errors to predict dependent variables. To estimate the main effect of ownership language, the key predictor is a binary variable indicating whether or not participants read one of the text messages containing the ownership language (*Ownership*). It equals 1 for participants in the *Ownership Reminder* or *Ownership Reminder with Video* condition and 0 otherwise. To estimate the main effect of our video, the key predictor is a binary variable indicating whether or not participants read one of the text messages containing the video (*Video*). It equals 1 for participants in the *Basic Reminder with Video* or *Ownership Reminder with Video* condition and 0 otherwise. To estimate the simple effect of ownership language (when the message does not contain the video), the simple effect of our video (when the message does not contain ownership language), and the interaction between these two interventions, our key predictors include the two binary indicators (Ownership and Video) as well as their interaction term.

Our regressions include a host of control variables, including an indicator for whether a participant was female ("Gender-Female"), an indicator for whether a participant's gender was either other or unknown ("Gender-Other"), participant age in years ("Age"), indicators for whether a participant was Hispanic/Latino ("Hispanic"), Black ("Black"), or Asian ("Asian"), an indicator for whether a participant either had other

race or did not report race information ("Race-Other/Unknown"), as well as an indicator for whether participants did not respond to demographic questions ("Missing Demographics"). "Black", "Asian", and "Race-Other/Unknown" were coded as 0 if a participant was Hispanic or Latino. All of the control variables were pre-registered except for "Missing Demographics." When pre-registering the study, we did not consider the possibility that some participants (n = 21) responded to our primary dependent variables but did not provide demographics. We treated those people's gender and race/ethnicity as unknown, assigned the average value of age as their age, and added a control for whether people missed reporting demographics.

All results reported in this section come from OLS regressions with the aforementioned controls, and we report two-sided p-values without adjustment for multiple comparisons.

2.2.1 Scheduling Likelihood

Supplementary Figure 4 Panel A displays the mean rating of scheduling likelihood for each message based on raw data, overlaying on a dot plot. Supplementary Table 25 Columns 1-2 report OLS regression results about scheduling likelihood. Column 1 indicates that adding ownership language did not have a statistically significant main effect at the 5% level (B = -0.15, SE = 0.10, p = 0.13). Instead, adding the video to the text message has a statistically significant effect at the 5% level: people overall indicated a greater likelihood of scheduling a vaccination appointment after receiving a message containing the video than after receiving a message without the video (B = 0.25, SE = 0.10, p = 0.01).

Furthermore, as shown in Column 2, we observed a positive simple effect of video whereby the *Basic Reminder with Video* message significantly increased people's self-reported likelihood of scheduling a vaccination appointment, relative to the *Basic Reminder* message (B = 0.40, SE = 0.13, p = 0.003). There is not a statistically significant simple effect of ownership language at the 5% level (B = -0.002, SE = 0.14, p = 0.99), neither is there a statistically significant interaction between the two interventions at the 5% level (B = -0.30, SE = 0.19, p = 0.13).

2.2.2 Message Persuasiveness

Supplementary Figure 4 Panel B displays the mean persuasiveness rating for each message based on raw data, overlaying on a dot plot. Supplementary Table 25 Columns 3-4 report OLS regression results about *Persuasiveness.* Column 3 shows a small, statistically significant main effect of adding ownership language: people rated the two messages containing the ownership language as more persuasive than the other two messages without such language (B = 0.18, SE = 0.08, p = 0.02). It shows a positive main effect of adding the video to the text message: people rated the two messages containing the video as significantly more persuasive than the two messages that do not contain the video (B = 1.42, SE = 0.07, $p < 0.001$).

Furthermore, as shown in Column 4, we observed a positive simple effect of ownership text whereby the *Ownership Reminder* message was viewed as more persuasive than the *Basic Reminder* message (B = 0.47, SE = 0.11, $p < 0.001$), and a positive simple effect of video whereby the *Basic Reminder with Video* message was perceived as more persuasive than the *Basic Reminder* message (B = 1.72, SE = 0.01, $p < 0.001$). We also observed a significant, negative interaction between the *Video* indicator and the *Ownership Reminder* indicator (B = -0.63, SE = 0.15, $p < 0.001$), which suggests that the impact of adding the video on persuasiveness and that of adding ownership language were not additive.

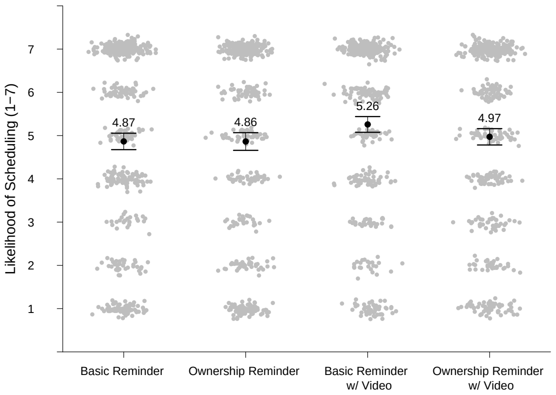

(a) Panel A: Likelihood of Scheduling

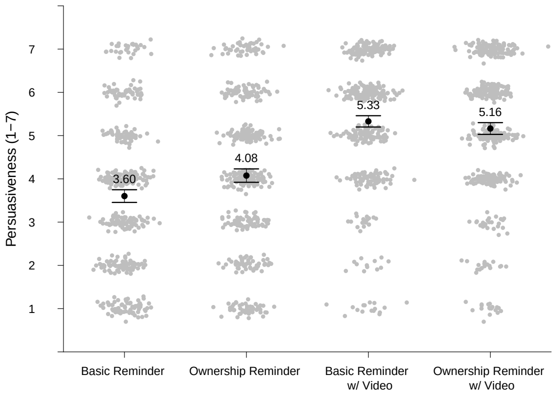

(b) Panel B: Persuasiveness

Figure 4: Likelihood of Scheduling and Persuasiveness by Message

Figure Legend: The scheduling likelihood and message persuasiveness for each condition are depicted here for the two online experiments (pooled) conducted in February 2021 (N=2,003 participants on Amazon Mechanical Turk and Prolific). Gray dots represent individual observations, with jittering in both dimensions for increased visibility, while the black dots represent condition means. Panel A displays participants' self-reported likelihood of scheduling a COVID-19 vaccination appointment upon receiving the message. Panel B displays participants' perceived persuasiveness of the text message. In both panels, error bars represent ±1 standard error of the corresponding mean. The number of observations in each condition (from left to right in each panel) is 520, 490, 492, and 501.

Table 25: Effects of Text Messages on Likelihood of Scheduling and Persuasiveness

Dependent Measure	Scheduling Likelihood		Persuasiveness	
	(1)	(2)	(3)	(4)
Ownership	-0.15	-0.00	0.16*	0.47***
	(0.10)	(0.14)	(0.07)	(0.11)
Video	0.25*	0.40**	1.41***	1.72***
	(0.10)	(0.13)	(0.07)	(0.10)
Ownership x Video		-0.30		-0.63***
		(0.19)		(0.15)
Gender-Female	-0.01	-0.01	0.17*	0.17*
	(0.10)	(0.10)	(0.07)	(0.07)
Gender-Other	0.92*	0.92*	0.38	0.38
	(0.41)	(0.41)	(0.39)	(0.39)
Age	-0.01	-0.01	0.01**	0.01**
	(0.00)	(0.00)	(0.00)	(0.00)
Hispanic	0.18	0.18	0.14	0.12
	(0.23)	(0.23)	(0.18)	(0.18)
Black	-0.51**	-0.52**	0.14	0.12
	(0.20)	(0.20)	(0.16)	(0.16)
Asian	0.74***	0.74***	0.02	0.01
	(0.13)	(0.13)	(0.12)	(0.12)
Race-Other/Unknown	0.02	0.02	-0.20	-0.19
	(0.22)	(0.22)	(0.16)	(0.16)
Missing Demographics	-0.87	-0.87	0.07	0.07
	(0.66)	(0.66)	(0.57)	(0.56)
Constant	5.16***	5.09***	3.36***	3.22***
	(0.18)	(0.19)	(0.13)	(0.14)
N	2003	2003	2003	2003
R^2	0.026	0.027	0.166	0.174

Legend: The table reports ordinary least squares (OLS) regressions predicting participants' self-reported likelihood of scheduling a COVID-19 vaccination appointment upon receiving the message (Columns 1-2) and their perceived persuasiveness of the text message (Columns 3-4). *Ownership* is a dummy variable coded as 1 when participants received one of the text messages containing the ownership language. Its p-value is 0.126, 0.987, 0.033, and < 0.001 in Columns 1-4, respectively. *Video* is a dummy variable coded as 1 when participants received one of the text messages containing the video. Its p-value is 0.010 in Column 1, 0.003 in Column 2, and < 0.001 in Columns 3-4. The interaction between *Ownership* and *Video* has a p-value of 0.128 in Column 2 and a p-value < 0.001 in Column 4. Control variables include an indicator for whether a participant was female ("Gender-Female"), an indicator for whether a participant's gender was either other or unknown ("Gender-Other"), participant age in years ("Age"), indicators for whether a participant was Hispanic/Latino ("Hispanic"), Black ("Black"), or Asian ("Asian"), an indicator for whether a participant either had other race or did not report race information ("Race-Other/Unknown"), as well as an indicator for whether participants did not respond to demographic questions ("Missing Demographics"). "Black", "Asian", and "Race-Other/Unknown" were coded as 0 if a participant was Hispanic or Latino. Robust standard errors are reported in parentheses. $^{*}p<0.05$; $^{**}p<0.01$; $^{***}p<0.001$ (all two-sided).

2.2.3 Mechanism for the Ownership Language

Next, we test how our ownership language changed people's psychological ownership of the vaccine and the perceived ease of getting the vaccine. Supplementary Table 26 Columns 1-2 report the results of OLS regressions that predict *Psychological Ownership* and *Easiness* as a function of an indicator for whether people read a text message containing the ownership language and the control variables described earlier. Consistent with the intention behind our design of the ownership language, the ownership language elevated participants' feeling that a COVID-19 vaccine was already theirs (B = 0.38, SE = 0.08, $p < 0.001$; Column 1). Also, we did not find that the ownership language affected people's perceptions of how easy it would be to receive the COVID-19 vaccine at the 5% significance level (B = 0.003, SE = 0.07, p = 0.97; Column 2). This helps address the concern that the text message containing the ownership language is longer and could increase the perceived complexity of getting vaccinated.

Table 26: Effects of Ownership Language on Psychological Ownership and Ease of Getting Vaccinated

Dependent Measure	Psychological Ownership	Easiness
	(1)	(2)
Ownership	0.38***	0.00
	(0.08)	(0.07)
Gender-Female	0.24**	0.18**
	(0.08)	(0.07)
Gender-Other	0.63	0.24
	(0.36)	(0.30)
Age	0.00	-0.00
	(0.00)	(0.00)
Hispanic	0.09	-0.19
	(0.21)	(0.18)
Black	-0.19	-0.27
	(0.18)	(0.16)
Asian	-0.14	-0.26*
	(0.14)	(0.11)
Race-Other/Unknown	-0.24	-0.10
	(0.19)	(0.15)
Missing Demographics	0.69	-0.55
	(0.61)	(0.81)
Constant	4.27***	5.50***
	(0.15)	(0.12)
N	1987	1987
R^2	0.018	0.009

Legend: The table reports ordinary least squares (OLS) regressions predicting the extent to which the message made participants feel that the COVID-19 vaccine was already theirs (Column 1) and the extent to which the message made them feel that getting the COVID-19 vaccine was easy (Column 2). *Ownership* is a dummy variable coded as 1 when participants received one of the text messages containing the ownership language. Its p-value is < 0.001 in Column 1 and 0.969 in Column 2. Control variables include an indicator for whether a participant was female ("Gender-Female"), an indicator for whether a participant's gender was either other or unknown ("Gender-Other"), participant age in years ("Age"), indicators for whether a participant was Hispanic/Latino ("Hispanic"), Black ("Black"), or Asian ("Asian"), an indicator for whether a participant either had other race or did not report race information ("Race-Other/Unknown"), as well as an indicator for whether participants did not respond to demographic questions ("Missing Demographics"). "Black", "Asian", and "Race-Other/Unknown" were coded as 0 if a participant was Hispanic or Latino. Robust standard errors are reported in parentheses. $^{*}p<0.05$; $^{**}p<0.01$; $^{***}p<0.001$ (all two-sided).

2.2.4 Mechanisms for the Video

Since we are interested in how the video may change people's beliefs and perceptions about COVID-19 and the vaccine, we focus on the main effect of the video. Supplementary Table 27 Columns 1-6 report the results of OLS regressions that predict each belief or perception measure as a function of an indicator for whether people read one of the text messages containing the video and the aforementioned control variables. As shown in Column 1, our video significantly increased people's predicted likelihood of getting COVID-19 without the vaccine by 2.79 percentage points (B = 2.79, SE = 1.11, p = 0.01), suggesting that the video made people believe COVID-19 is more prevalent and easier to transmit. As shown in Column 2, *Infection Likelihood with Vaccine* was significantly lower by 2.21 percentage points among people who were assigned to watch the video than among those who were not assigned to watch the video (B = -2.21, SE = 0.83, p = 0.01), suggesting that watching the video led people to believe they would be less likely to get COVID-19 with the vaccine. Furthermore, Column 3 shows that *Vaccine Effectiveness*—which we calculated by subtracting *Infection Likelihood with Vaccine* from *Infection Likelihood without Vaccine*—was significantly higher by 5.01 percentage points in the two conditions involving the video than in the other two conditions (B = 5.01, SE = 1.28, $p < 0.001$). This suggests that watching the video boosted people's belief that the vaccine could reduce their infection likelihood.

In addition, watching the video led participants to feel more worried about the possibility of transmitting COVID-19 to others (B = 0.26, SE = 0.09, p = 0.003; Column 4). We did not find evidence that our video could change people's anticipated regret of not getting vaccinated (B = 0.13, SE = 0.10, p = 0.17; Column 5) or their trust in the safety and development process of the authorized vaccines (B = 0.003, SE = 0.08, p = 0.97; Column 6) at the 5% significance level.

We also conducted a mediation analysis to explore which of these beliefs and perceptions drove the effect of the video on scheduling likelihood. We ran a multi-mediator model[54] that included *Infection Likelihood without Vaccine*, *Vaccine Effectiveness*, *Worry of Transmission*, *Anticipated Regret*, and *Trust in Vaccine* as potential mediators and kept the aforementioned demographic controls. Since *Vaccine Effectiveness* equals *Infection Likelihood without Vaccine* minus *Infection Likelihood with Vaccine*, we could not include all three of them in the mediation model. We focused on *Infection Likelihood without Vaccine* rather than *Infection Likelihood with Vaccine* because the former captured participants' baseline belief in the prevalence of COVID-19 and their chance of being infected. We used 5,000 bootstrapped samples. We found evidence of partial mediation. Specifically, we estimated a positive indirect effect of *Vaccine Effectiveness* (B = 0.03, SE = 0.01), and the 95% bias-corrected confidence interval (CI) excluded 0 ([0.01, 0.06]). We also estimated a positive indirect effect of *Worry of Transmission* (B = 0.015, SE = 0.007), and the 95% bias-corrected CI excluded 0 ([0.004, 0.03]). These results suggest that watching the video significantly boosted people's beliefs in the effectiveness of the existing vaccine and elevated their worry of transmission to others, which further increased their reported likelihood of scheduling a vaccination appointment.

Table 27: Effects of Video on Beliefs and Perceptions

Dependent Measure	Infection Likelihood w/o Vaccine	Infection Likelihood w/ Vaccine	Vaccine Effectiveness	Worry	Anticipated Regret	Trust
	(1)	(2)	(3)	(4)	(5)	(6)
Video	2.79*	-2.21**	5.01***	0.26**	0.13	0.00
	(1.11)	(0.83)	(1.28)	(0.09)	(0.10)	(0.08)
Gender-Female	8.82***	6.18***	2.63*	0.48***	0.18	-0.27***
	(1.12)	(0.83)	(1.27)	(0.09)	(0.10)	(0.08)
Gender-Other	14.79**	3.40	11.39	1.21***	1.19***	0.76*
	(4.98)	(3.52)	(6.02)	(0.27)	(0.24)	(0.38)
Age	-0.15***	0.03	-0.19***	-0.01***	-0.00	-0.02***
	(0.04)	(0.03)	(0.05)	(0.00)	(0.00)	(0.00)
Hispanic	6.66*	5.09*	1.57	0.17	0.23	-0.07
	(2.63)	(2.44)	(3.10)	(0.21)	(0.23)	(0.19)
Black	1.24	7.40***	-6.16*	-0.21	0.01	-0.59***
	(2.31)	(2.14)	(2.91)	(0.18)	(0.20)	(0.16)
Asian	2.38	0.96	1.42	0.27*	0.72***	0.24*
	(1.85)	(1.36)	(2.05)	(0.14)	(0.13)	(0.11)
Race-Other/Unknown	-0.70	1.61	-2.31	-0.06	0.30	-0.14
	(2.55)	(1.82)	(2.60)	(0.20)	(0.20)	(0.17)
Missing Demographics	-2.74	-0.64	-2.10	-0.77	-0.63	-0.82
	(8.58)	(6.83)	(10.68)	(0.61)	(0.46)	(0.60)
Constant	46.32***	12.86***	33.46***	4.84***	5.14***	5.05***
	(2.01)	(1.47)	(2.29)	(0.16)	(0.17)	(0.14)
N	1992	1992	1992	1991	1991	1988
R^2	0.044	0.040	0.022	0.034	0.019	0.033

Legend: The table reports ordinary least squares (OLS) regressions predicting *Infection Likelihood without Vaccine* in Column 1, *Infection Likelihood with Vaccine* in Column 2, *Vaccine Effectiveness* in Column 3, *Worry of Transmission* in Column 4, *Anticipated Regret* in Column 5, and *Trust in Vaccine* in Column 6. We multiplied participants' original responses by 100 for *Infection Likelihood without Vaccine* (ranging from 0 to 100), *Infection Likelihood with Vaccine* (ranging from 0 to 100), and *Vaccine Effectiveness* (ranging from -100 to 100). *Video* is a dummy variable coded as 1 when participants received one of the text messages containing the video. Its p-value is 0.012, 0.008, < 0.001, 0.003, 0.172, and 0.966 in Columns 1-6, respectively. Control variables include an indicator for whether a participant was female ("Gender-Female"), an indicator for whether a participant's gender was either other or unknown ("Gender-Other"), participant age in years ("Age"), indicators for whether a participant was Hispanic/Latino ("Hispanic"), Black ("Black"), or Asian ("Asian"), an indicator for whether a participant either had other race or did not report race information ("Race-Other/Unknown"), as well as an indicator for whether participants did not respond to demographic questions ("Missing Demographics"). "Black", "Asian", and "Race-Other/Unknown" were coded as 0 if a participant was Hispanic or Latino. Robust standard errors are reported in parentheses. $^{*}p<0.05$; $^{**}p<0.01$; $^{***}p<0.001$ (all two-sided).

2.2.5 Heterogeneous Treatment Effects

Finally, as pre-registered, we explored how the effects of our interventions on scheduling likelihood and persuasiveness differed across the different sub-populations studied, including participants who identified themselves as Democrats or Republicans, participants who did or did not receive the flu shot at least once during the two most recent seasons, participants with or without a bachelor's degree, participants who self-reported washing their hands at a high or low frequency when coming back home from outside, participants who self-reported wearing masks outside at a high or low frequency, and participants with or without children under 18. For the frequency with which people washed their hands when coming back home and the frequency with which people wore masks when outside, we pre-registered that we would do a median split. The median response is 5 for both questions, corresponding to "Always or almost always." Thus, we essentially separated participants into two buckets for each question based on whether or not they reported always washing their hands (or always wearing masks).

Since (1) we did not find a statistically significant interaction at the 5% level between the video intervention and ownership language in predicting participants' self-reported likelihood of scheduling (B = -0.30, SE = 0.19, p = 0.13 in Column 2 of Supplementary Table 25) and (2) we are primarily interested in the main effects of these two manipulations, we focused on exploring the heterogeneous treatment effects for the main effects of video and ownership language.

Given that we largely did not see significant differences between sub-populations, we only report here heterogeneous treatment effects based on political affiliations, which may be of the most interest to readers (Supplementary Table 28). Results about other sub-sample analyses are available at https://osf.io/qn8hr/?view_only=cf7b2bc590054aee8c4a2bae99ef20c5.

Table 28: Main Effects of Text Message on Scheduling Likelihood and Persuasiveness for Democrats and Republicans

Dependent Measure	Scheduling Likelihood			Persuasiveness		
Sample	Democrat	Republican	Both	Democrat	Republican	Both
	(1)	(2)	(3)	(4)	(5)	(6)
Ownership	-0.03	-0.24	-0.24	0.24**	0.09	0.09
	(0.10)	(0.15)	(0.15)	(0.09)	(0.11)	(0.11)
Video	0.17+	0.32*	0.31*	1.49***	1.33***	1.32***
	(0.10)	(0.15)	(0.15)	(0.09)	(0.11)	(0.11)
Democrat			2.07***			0.63***
			(0.15)			(0.13)
Ownership x Democrat			0.21			0.15
			(0.18)			(0.14)
Video x Democrat			-0.14			0.18
			(0.18)			(0.14)
Gender-Female	0.01	-0.25+	-0.13	0.06	0.22+	0.13+
	(0.09)	(0.15)	(0.09)	(0.09)	(0.11)	(0.07)
Gender-Other	0.04	0.85*	0.07	-0.14	2.97***	0.06
	(0.43)	(0.37)	(0.40)	(0.39)	(0.25)	(0.40)
Age	0.00	0.01*	0.01 +	0.01***	0.01**	0.01***
	(0.00)	(0.01)	(0.00)	(0.00)	(0.00)	(0.00)
Hispanic	-0.25	-0.14	-0.15	0.09	-0.27	-0.02
	(0.21)	(0.46)	(0.21)	(0.21)	(0.29)	(0.17)
Black	-1.36***	-0.35	-1.05***	-0.19	0.26	-0.06
	(0.21)	(0.41)	(0.19)	(0.17)	(0.34)	(0.16)
Asian	-0.12	0.99**	0.23+	-0.28*	0.14	-0.17
	(0.13)	(0.32)	(0.13)	(0.14)	(0.24)	(0.12)
Race-Other/Unknown	-0.45+	0.27	-0.09	-0.29	-0.25	-0.25
	(0.24)	(0.36)	(0.21)	(0.19)	(0.25)	(0.15)
Missing Demographics	-0.28	-0.80	-0.09	0.67	-2.59***	0.36
	(0.77)	(0.77)	(0.67)	(0.58)	(0.64)	(0.59)
Constant	6.01***	3.49***	3.72***	3.54***	2.87***	2.89***
	(0.19)	(0.27)	(0.19)	(0.17)	(0.20)	(0.15)
N	1037	942	1979	1037	942	1979
R^2	0.067	0.026	0.236	0.230	0.146	0.212

Legend: The table reports ordinary least squares (OLS) regressions predicting participants' self-reported likelihood of scheduling a COVID-19 vaccination appointment upon receiving the message (Columns 1-3) and their perceived persuasiveness of the text message (Columns 4-6), broken down by political affiliation. Columns 1 and 4 focus on participants who self-identified as Democrats, Columns 2 and 5 focus on participants who self-identified as Republicans, and Columns 3 and 6 focus on the combined sample (excluding people who did not report identifying with either party). *Ownership* is a dummy variable coded as 1 when participants received one of the text messages containing the ownership language. Its p-value is 0.786, 0.112, 0.109, 0.008, 0.406, and 0.425 in Columns 1-6, respectively. *Video* is a dummy variable coded as 1 when participants received one of the text messages containing the video. Its p-value is 0.071 in Column 1, 0.029 in Column 2, and 0.035 in Column 3, and < 0.001 in Columns 4-6. The interaction between *Ownership* and *Democrat* has a p-value of 0.245 in Column 3 and 0.296 in Column 6. The interaction between *Video* and *Democrat* has a p-value of 0.427 in Column 3 and 0.210 in Column 6. Control variables include an indicator for whether a participant was female ("Gender-Female"), an indicator for whether a participant's gender was either other or unknown ("Gender-Other"), participant age in years ("Age"), indicators for whether a participant was Hispanic/Latino ("Hispanic"), Black ("Black"), or Asian ("Asian"), an indicator for whether a participant either had other race or did not report race information ("Race-Other/Unknown"), as well as an indicator for whether participants did not respond to demographic questions ("Missing Demographics"). "Black", "Asian", and "Race-Other/Unknown" were coded as 0 if a participant was Hispanic or Latino. Robust standard errors are reported in parentheses. +p<0.10; *p<0.05; **p<0.01; ***p<0.001 (all two-sided).

2.2.6 Separate Results for the First and Second Online Experiments Conducted in February 2021

Table 29: Experiment 1: Effects of Text Messages on Likelihood of Scheduling and Persuasiveness

Dependent Measure	Scheduling Likelihood		Persuasiveness	
	(1)	(2)	(3)	(4)
Ownership	-0.16	-0.03	0.13	0.42**
	(0.13)	(0.18)	(0.09)	(0.14)
Video	0.22+	0.35+	1.43***	1.72***
	(0.13)	(0.18)	(0.09)	(0.13)
Ownership x Video		-0.25		-0.59**
		(0.26)		(0.19)
Gender-Female	-0.15	-0.15	0.18	0.18
	(0.13)	(0.13)	(0.10)	(0.10)
Gender-Other	1.51**	1.50**	1.00	0.99
	(0.53)	(0.53)	(0.59)	(0.60)
Age	0.00	0.00	0.01**	0.01**
	(0.01)	(0.01)	(0.00)	(0.00)
Hispanic	0.45	0.45	0.14	0.13
	(0.34)	(0.34)	(0.25)	(0.26)
Black	-0.22	-0.23	0.21	0.20
	(0.24)	(0.24)	(0.19)	(0.19)
Asian	0.76***	0.75***	0.00	-0.01
	(0.20)	(0.20)	(0.17)	(0.17)
Race-Other/Unknown	-0.33	-0.32	-0.43	-0.41
	(0.33)	(0.34)	(0.24)	(0.24)
Missing Demographics	-2.15*	-2.14*	-1.16	-1.15
	(0.94)	(0.94)	(0.86)	(0.87)
Constant	4.84***	4.79***	3.22***	3.10***
	(0.25)	(0.26)	(0.18)	(0.18)
N	1163	1163	1163	1163
R^2	0.023	0.024	0.177	0.183

Legend: The table reports ordinary least squares (OLS) regressions predicting participants' self-reported likelihood of scheduling a COVID-19 vaccination appointment upon receiving the message (Columns 1-2) and their perceived persuasiveness of the text message (Columns 3-4) for participants included in the first online experiment conducted in February 2021. *Ownership* is a dummy variable coded as 1 when participants received one of the text messages containing the ownership language. Its p-value is 0.219, 0.851, 0.160, and 0.003 in Columns 1-4, respectively. *Video* is a dummy variable coded as 1 when participants received one of the text messages containing the video. Its p-value is 0.090 in Column 1, 0.054 in Column 2, and < 0.001 in Columns 3-4. The interaction between *Ownership* and *Video* has a p-value of 0.325 in Column 2 and 0.002 in Column 4. Control variables include an indicator for whether a participant was female ("Gender-Female"), an indicator for whether a participant's gender was either other or unknown ("Gender-Other"), participant age in years ("Age"), indicators for whether a participant was Hispanic/Latino ("Hispanic"), Black ("Black"), or Asian ("Asian"), an indicator for whether a participant either had other race or did not report race information ("Race-Other/Unknown"), as well as an indicator for whether participants did not respond to demographic questions ("Missing Demographics"). "Black", "Asian", and "Race-Other/Unknown" were coded as 0 if a participant was Hispanic or Latino. Robust standard errors are reported in parentheses. $^{*}p<0.05$; $^{**}p<0.01$; $^{***}p<0.001$ (all two-sided).

Table 30: Experiment 1: Effects of Ownership Language on Psychological Ownership and Ease of Getting Vaccinated

Dependent Measure	Psychological Ownership	Easiness
	(1)	(2)
Ownership	0.33**	0.02
	(0.11)	(0.09)
Gender-Female	0.20	0.12
	(0.11)	(0.09)
Gender-Other	0.91	0.11
	(0.54)	(0.51)
Age	0.01*	-0.00
	(0.00)	(0.00)
Hispanic	-0.10	0.00
	(0.30)	(0.20)
Black	-0.17	-0.06
	(0.22)	(0.17)
Asian	-0.13	-0.19
	(0.19)	(0.15)
Race-Other/Unknown	-0.32	-0.26
	(0.27)	(0.24)
Missing Demographics	1.12*	0.76
	(0.53)	(0.49)
Constant	3.92***	5.43***
	(0.21)	(0.16)
N	1155	1155
R^2	0.020	0.005

Legend: The table reports ordinary least squares (OLS) regressions predicting the extent to which the message made participants feel that the COVID-19 vaccine was already theirs (Column 1) and the extent to which the message made them feel that getting the COVID-19 vaccine was easy (Column 2) for participants included in the first online experiment conducted in February 2021. *Ownership* is a dummy variable coded as 1 when participants received one of the text messages containing the ownership language. Its p-value is 0.003 in Column 1 and 0.785 in Column 2. Control variables include an indicator for whether a participant was female ("Gender-Female"), an indicator for whether a participant's gender was either other or unknown ("Gender-Other"), participant age in years ("Age"), indicators for whether a participant was Hispanic/Latino ("Hispanic"), Black ("Black"), or Asian ("Asian"), an indicator for whether a participant either had other race or did not report race information ("Race-Other/Unknown"), as well as an indicator for whether participants did not respond to demographic questions ("Missing Demographics"). "Black", "Asian", and "Race-Other/Unknown" were coded as 0 if a participant was Hispanic or Latino. Robust standard errors are reported in parentheses. $^{*}p<0.05$; $^{**}p<0.01$; $^{***}p<0.001$ (all two-sided).

Table 31: Experiment 1: Effects of Video on Beliefs and Perceptions

Dependent Measure	Infection Likelihood w/o Vaccine	Infection Likelihood w/ Vaccine	Vaccine Effectiveness	Worry	Anticipated Regret	Trust
	(1)	(2)	(3)	(4)	(5)	(6)
Video	3.41*	-1.65	5.06**	0.29*	0.12	-0.05
	(1.44)	(1.12)	(1.69)	(0.12)	(0.13)	(0.10)
Gender-Female	10.17***	7.58***	2.58	0.61***	0.11	-0.43***
	(1.45)	(1.11)	(1.67)	(0.12)	(0.13)	(0.11)
Gender-Other	23.12***	8.04	15.08	1.77***	1.52***	1.14**
	(7.00)	(5.68)	(9.22)	(0.34)	(0.32)	(0.36)
Age	-0.09	-0.04	-0.05	-0.01*	0.01	-0.01
	(0.06)	(0.05)	(0.07)	(0.00)	(0.01)	(0.00)
Hispanic	6.53	4.79	1.75	-0.07	0.15	0.11
	(3.44)	(3.23)	(4.83)	(0.29)	(0.36)	(0.29)
Black	0.95	7.72**	-6.77*	-0.09	0.27	-0.43*
	(2.71)	(2.63)	(3.25)	(0.22)	(0.23)	(0.20)
Asian	2.47	1.82	0.65	0.30	0.73***	0.26
	(2.52)	(2.01)	(3.00)	(0.18)	(0.20)	(0.16)
Race-Other/Unknown	-4.75	1.01	-5.76	-0.45	-0.09	-0.53*
	(3.54)	(2.55)	(3.90)	(0.30)	(0.31)	(0.24)
Missing Demographics	-3.07	17.89**	-20.96*	0.26	-0.49	-1.89***
	(7.60)	(5.88)	(9.39)	(0.37)	(0.34)	(0.40)
Constant	42.18***	15.05***	27.13***	4.55***	4.72***	4.74***
	(2.75)	(2.09)	(3.23)	(0.22)	(0.23)	(0.19)
N	1155	1155	1155	1155	1155	1155
R^2	0.053	0.052	0.018	0.040	0.015	0.033

Legend: The table reports ordinary least squares (OLS) regressions predicting *Infection Likelihood without Vaccine* in Column 1, *Infection Likelihood with Vaccine* in Column 2, *Vaccine Effectiveness* in Column 3, *Worry of Transmission* in Column 4, *Anticipated Regret* in Column 5, and *Trust in Vaccine* in Column 6 for participants included in the first online experiment conducted in February 2021. We multiplied participants' original responses by 100 for *Infection Likelihood without Vaccine* (ranging from 0 to 100), *Infection Likelihood with Vaccine* (ranging from 0 to 100), and *Vaccine Effectiveness* (ranging from -100 to 100). *Video* is a dummy variable coded as 1 when participants received one of the text messages containing the video. Its p-value is 0.018, 0.139, 0.003, 0.012, 0.350, and 0.633 in Columns 1-6, respectively. Control variables include an indicator for whether a participant was female ("Gender-Female"), an indicator for whether a participant's gender was either other or unknown ("Gender-Other"), participant age in years ("Age"), indicators for whether a participant was Hispanic/Latino ("Hispanic"), Black ("Black"), or Asian ("Asian"), an indicator for whether a participant either had other race or did not report race information ("Race-Other/Unknown"), as well as an indicator for whether participants did not respond to demographic questions ("Missing Demographics"). "Black", "Asian", and "Race-Other/Unknown" were coded as 0 if a participant was Hispanic or Latino. Robust standard errors are reported in parentheses. *p<0.05; **p<0.01; ***p<0.001 (all two-sided).

Table 32: Experiment 2: Effects of Text Messages on Likelihood of Scheduling and Persuasiveness

Dependent Measure	Scheduling Likelihood		Persuasiveness	
	(1)	(2)	(3)	(4)
Ownership	-0.12	0.07	0.20	0.54**
	(0.15)	(0.22)	(0.11)	(0.17)
Video	0.30*	0.49*	1.40***	1.73***
	(0.15)	(0.20)	(0.11)	(0.16)
Ownership x Video		-0.38		-0.68**
		(0.29)		(0.23)
Gender-Female	0.17	0.19	0.14	0.16
	(0.15)	(0.15)	(0.11)	(0.11)
Gender-Other	0.31	0.31	-0.30	-0.29
	(0.62)	(0.65)	(0.44)	(0.43)
Age	-0.02**	-0.02**	0.01	0.01
	(0.01)	(0.01)	(0.00)	(0.00)
Hispanic	-0.11	-0.12	0.11	0.10
	(0.33)	(0.33)	(0.26)	(0.26)
Black	-1.11**	-1.13**	0.00	-0.03
	(0.35)	(0.35)	(0.28)	(0.28)
Asian	0.67***	0.67***	0.03	0.03
	(0.17)	(0.17)	(0.17)	(0.17)
Race-Other/Unknown	0.29	0.29	-0.01	-0.02
	(0.28)	(0.28)	(0.20)	(0.20)
Missing Demographics	0.26	0.26	1.18	1.17
	(0.88)	(0.88)	(0.63)	(0.61)
Constant	5.43***	5.34***	3.49***	3.32***
	(0.27)	(0.28)	(0.20)	(0.22)
N	840	840	840	840
R^2	0.049	0.051	0.163	0.172

Legend: The table reports ordinary least squares (OLS) regressions predicting participants' self-reported likelihood of scheduling a COVID-19 vaccination appointment upon receiving the message (Columns 1-2) and their perceived persuasiveness of the text message (Columns 3-4) for participants included in the second online experiment conducted in February 2021. *Ownership* is a dummy variable coded as 1 when participants received one of the text messages containing the ownership language. Its p-value is 0.408, 0.750, 0.078, and 0.002 in Columns 1-4, respectively. *Video* is a dummy variable coded as 1 when participants received one of the text messages containing the video. Its p-value is 0.037 in Column 1, 0.014 in Column 2, and < 0.001 in Columns 3-4. The interaction between *Ownership* and *Video* has a p-value of 0.199 in Column 2 and 0.003 in Column 4. Control variables include an indicator for whether a participant was female ("Gender-Female"), an indicator for whether a participant's gender was either other or unknown ("Gender-Other"), participant age in years ("Age"), indicators for whether a participant was Hispanic/Latino ("Hispanic"), Black ("Black"), or Asian ("Asian"), an indicator for whether a participant either had other race or did not report race information ("Race-Other/Unknown"), as well as an indicator for whether participants did not respond to demographic questions ("Missing Demographics"). "Black", "Asian", and "Race-Other/Unknown" were coded as 0 if a participant was Hispanic or Latino. Robust standard errors are reported in parentheses. $^{*}p<0.05$; $^{**}p<0.01$; $^{***}p<0.001$ (all two-sided).

Table 33: Experiment 2: Effects of Ownership Language on Psychological Ownership and Ease of Getting Vaccinated

Dependent Measure	Psychological Ownership	Easiness
	(1)	(2)
Ownership	0.46***	-0.02
	(0.13)	(0.11)
Gender-Female	0.28*	0.26*
	(0.13)	(0.11)
Gender-Other	0.28	0.50
	(0.48)	(0.29)
Age	-0.00	-0.00
	(0.00)	(0.00)
Hispanic	0.20	-0.39
	(0.29)	(0.29)
Black	-0.13	-0.70*
	(0.31)	(0.32)
Asian	-0.18	-0.36*
	(0.20)	(0.16)
Race-Other/Unknown	-0.21	0.04
	(0.26)	(0.17)
Missing Demographics	0.74	-1.21
	(0.79)	(0.96)
Constant	4.54***	5.57***
	(0.21)	(0.17)
N	832	832
R^2	0.025	0.025

Legend: The table reports ordinary least squares (OLS) regressions predicting the extent to which the message made participants feel that the COVID-19 vaccine was already theirs (Column 1) and the extent to which the message made them feel that getting the COVID-19 vaccine was easy (Column 2) for participants included in the second online experiment conducted in February 2021. *Ownership* is a dummy variable coded as 1 when participants received one of the text messages containing the ownership language. Its p-value is < 0.001 in Column 1 and 0.817 in Column 2. Control variables include an indicator for whether a participant was female ("Gender-Female"), an indicator for whether a participant's gender was either other or unknown ("Gender-Other"), participant age in years ("Age"), indicators for whether a participant was Hispanic/Latino ("Hispanic"), Black ("Black"), or Asian ("Asian"), an indicator for whether a participant either had other race or did not report race information ("Race-Other/Unknown"), as well as an indicator for whether participants did not respond to demographic questions ("Missing Demographics"). "Black", "Asian", and "Race-Other/Unknown" were coded as 0 if a participant was Hispanic or Latino. Robust standard errors are reported in parentheses. *p<0.05; **p<0.01; ***p<0.001 (all two-sided).

Table 34: Experiment 2: Effects of Video on Beliefs and Perceptions

Dependent Measure	Infection Likelihood w/o Vaccine	Infection Likelihood w/ Vaccine	Vaccine Effectiveness	Worry	Anticipated Regret	Trust
	(1)	(2)	(3)	(4)	(5)	(6)
Video	1.82	-2.84*	4.67*	0.20	0.13	0.07
	(1.75)	(1.26)	(1.94)	(0.13)	(0.14)	(0.12)
Gender-Female	6.80***	4.48***	2.32	0.30*	0.25	-0.07
	(1.77)	(1.27)	(1.96)	(0.14)	(0.15)	(0.12)
Gender-Other	6.33	-1.71	8.04	0.72	0.92*	0.42
	(6.36)	(2.41)	(7.06)	(0.39)	(0.38)	(0.69)
Age	-0.22**	0.09	-0.31***	-0.02***	-0.02**	-0.03***
	(0.07)	(0.05)	(0.07)	(0.01)	(0.01)	(0.00)
Hispanic	6.26	5.62	0.64	0.34	0.24	-0.28
	(3.95)	(3.60)	(4.03)	(0.30)	(0.29)	(0.26)
Black	2.69	6.34	-3.65	-0.40	-0.50	-0.94***
	(4.39)	(3.68)	(5.97)	(0.34)	(0.37)	(0.25)
Asian	2.06	0.44	1.62	0.22	0.66***	0.17
	(2.72)	(1.80)	(2.74)	(0.20)	(0.18)	(0.16)
Race-Other/Unknown	2.63	2.09	0.55	0.28	0.65*	0.20
	(3.66)	(2.62)	(3.44)	(0.26)	(0.27)	(0.23)
Missing Demographics	0.31	1.12	-0.81	-0.91	-0.74	-0.53
	(10.34)	(6.75)	(12.10)	(0.74)	(0.61)	(0.87)
Constant	50.87***	11.44***	39.43***	5.16***	5.56***	5.33***
	(2.96)	(2.11)	(3.26)	(0.23)	(0.25)	(0.20)
N	837	837	837	836	836	833
R^2	0.039	0.034	0.035	0.035	0.041	0.058

Legend: The table reports ordinary least squares (OLS) regressions predicting *Infection Likelihood without Vaccine* in Column 1, *Infection Likelihood with Vaccine* in Column 2, *Vaccine Effectiveness* in Column 3, *Worry of Transmission* in Column 4, *Anticipated Regret* in Column 5, and *Trust in Vaccine* in Column 6 for participants included in the second online experiment conducted in February 2021. We multiplied participants' original responses by 100 for *Infection Likelihood without Vaccine* (ranging from 0 to 100), *Infection Likelihood with Vaccine* (ranging from 0 to 100), and *Vaccine Effectiveness* (ranging from -100 to 100). *Video* is a dummy variable coded as 1 when participants received one of the text messages containing the video. Its p-value is 0.296, 0.025, 0.016, 0.133, 0.368, and 0.556 in Columns 1-6, respectively. Control variables include an indicator for whether a participant was female ("Gender-Female"), an indicator for whether a participant's gender was either other or unknown ("Gender-Other"), participant age in years ("Age"), indicators for whether a participant was Hispanic/Latino ("Hispanic"), Black ("Black"), or Asian ("Asian"), an indicator for whether a participant either had other race or did not report race information ("Race-Other/Unknown"), as well as an indicator for whether participants did not respond to demographic questions ("Missing Demographics"). "Black", "Asian", and "Race-Other/Unknown" were coded as 0 if a participant was Hispanic or Latino. Robust standard errors are reported in parentheses. $^{*}p<0.05$; $^{**}p<0.01$; $^{***}p<0.001$ (all two-sided).

3 Replication Online Experiment Assessing Intentions and Mechanisms in April 2021

We conducted another pre-registered online experiment late April 2021 with two primary objectives. First, we sought to examine whether the patterns observed in our earlier online experiments conducted in February 2021 still hold late April 2021 when the U.S had reached universal access to the COVID-19 vaccine. Second, we wanted to test whether findings from our earlier online experiments about vaccination intentions are robust to different ways of soliciting intentions. To that end, we varied whether participants reported their interest in getting vaccinated in the same hypothetical manner as in our previous online experiments or whether participants responded to questions that had less of an hypothetical framing. In a nutshell, regardless of the way we measured interest in getting vaccinated, we replicated the findings about vaccination intentions from our earlier online experiments.

3.1 Method

3.1.1 Participants

We began our data collection on MTurk and planned to end our data collection either when we hit the end of our seven-day data collection period or when we recruited 1,000 participants who fit our pre-registered selection criteria (see pre-registration at https://aspredicted.org/blind.php?x=7wf9er). The only deviation from the pre-registration is that we pre-registered to only run data collection from 9am PST to 7pm PST. To increase our sample size, we started data collection from 6am PST from the third day on. Data collection on MTurk was much slower than we had expected: After five days, we only recruited around 500 participants. This may happen because we were only looking for people who had not yet gotten vaccinated and did not have a vaccination appointment and we had to screen out participants who had taken our earlier experiments. Thus, we decided to expand our data collection to Prolific and pre-registered that we would recruit 400 participants from Prolific who satisfied our selection criteria in the hope of bringing our final combined sample to about 1,000 (see pre-registration at https://aspredicted.org/blind.php?x=u82hy5). Study design, selection criteria, and analysis plan were identical between participants recruited from MTurk and Prolific. Recruiting participants from Prolific, in hindsight, also allows us to better compare our results between experiments since our earlier online experiments included both MTurk and Prolific populations.

We pre-registered that we would only include in our analysis participants who (1) passed a Captcha and an attention check question, (2) had not already received the COVID-19 vaccine and did not already have a COVID-19 vaccination appointment, (3) completed at the minimum the portion of the survey involving our pre-registered primary dependent variables, (4) did not report having technical issues with watching the video, and (5) did not report having taken a similar study on Prolific (if participants were recruited from MTurk) or Mturk (if participants were from Prolific). These criteria were similar to the criteria of our February 2021 online experiments. We did not balance Republicans and Democrats in the recruitment stage as we did for the earlier online experiments because (1) Democrats and Republicans did not behave meaningfully differently in our previous experiments and (2) it would take much more time to get a large sample of republicans. If a person took the survey and completed our pre-registered dependent variables more than once, we kept their first response. Our sample consists of 1,178 participants (53.40% female, M_{age} = 36.75) who satisfied the aforementioned criteria.

3.1.2 Procedure and Measures

The procedure and measures were identical to those of our earlier online experiments conducted in February 2021 (see details in Sections 2.1.2 and 2.1.3), with a few exceptions. The complete survey is available at https://osf.io/qn8hr/?view_only=cf7b2bc590054aee8c4a2bae99ef20c5.

- First, since all US residents became eligible for the COVID-19 vaccine, we no longer asked participants to imagine that they became eligible for the COVID-19 vaccine. We simply instructed participants to "Imagine that your healthcare provider sends you the following message today."
- Second, we used two items to measure interest in getting vaccinated. The first item assessed people's likelihood of scheduling a vaccination appointment (the same as what we used in the earlier online experiments). The second item assessed how much people wanted the vaccine.
- Third, for half of the participants, we solicited their interest in getting vaccinated in a hypothetical manner exactly as in our earlier experiments: "How likely would you be to schedule a vaccination appointment after receiving this message from your healthcare provider?" (from 1 = Not at all likely to 7 = Very likely) and "How much would you want the vaccine after receiving this message from your healthcare provider?" (from 1 = Not at all to 7 = Very much). These two items were highly correlated ($r = 0.94$, $p < 0.0001$) and were averaged to form a composite score of *Hypothetical Interest in Getting the Vaccine*. For another half of the participants, we constructed the measures in the present tense and sought to assess their current interest: "How likely are you to schedule a vaccination appointment *today* after reading this message from your healthcare provider?" (from 1 = Not at all likely to 7 = Very likely) and "How much do you want the vaccine *now* after receiving this message from your healthcare provider?" (from 1 = Not at all to 7 = Very much; italics were not actually part of the questions and were added here to highlight that we assessed current interest). These two items were highly correlated ($r = 0.93$, $p < 0.0001$) and were averaged to form a composite score of *Current Interest in Getting the Vaccine*. Notably, our measure of *Persuasiveness* was the same across all participants: "How persuasive do you think the message is?" (from 1 = Not at all persuasive to 7 = Very persuasive). Nevertheless, we report results by separately analyzing *Persuasiveness* among participants who answered the hypothetical version of vaccination interest questions versus participants who reported their current interest in getting the vaccine. For simplicity, hereafter, we use *Persuasiveness-Hypothetical* and *Persuasiveness-Current* to refer to the persuasiveness measure for these two subsets of participants, respectively.
- Finally, as a minor difference, we did not measure the extent to which the text message made participants feel that getting the COVID-19 vaccine is easy, because this measure did not statistically significantly differ between conditions in the earlier online experiments.

3.2 Results

As pre-registered, we used ordinary least squares (OLS) regressions with robust standard errors to predict dependent variables. To estimate the main effect of ownership language, the key predictor is a binary variable indicating whether or not participants read one of the text messages containing the ownership language (*Ownership*). It equals 1 for participants in the *Ownership Reminder* or *Ownership Reminder with*

Video condition and 0 otherwise. To estimate the main effect of our video, the key predictor is a binary variable indicating whether or not participants read one of the text messages containing the video (*Video*). It equals 1 for participants in the *Basic Reminder with Video* or *Ownership Reminder with Video* condition and 0 otherwise. Since (1) the earlier online experiments did not identify robustly statistically significant interactions at the 5% level between the Ownership intervention and the Video intervention in predicting likelihood of scheduling a vaccination appointment, and (2) our primary interest lies in assessing the main effects of these two interventions, we did not pre-register to look at the interaction in this experiment.

All OLS regressions include the same set of control variables as in our analyses of the earlier online experiments, including an indicator for whether a participant was female ("Gender-Female"), an indicator for whether a participant's gender was either other or unknown ("Gender-Other"), participant age in years ("Age"), indicators for whether a participant was Hispanic/Latino ("Hispanic"), Black ("Black"), or Asian ("Asian"), an indicator for whether a participant either had other race or did not report race information ("Race-Other/Unknown"), as well as an indicator for whether participants did not respond to demographic questions ("Missing Demographics"). "Black", "Asian", and "Race-Other/Unknown" were coded as 0 if a patient was Hispanic or Latino. For 12 participants who responded to our primary dependent variables but did not provide demographics, we treated those people's gender and race/ethnicity as unknown, assigned the average value of age as their age, and added a control for whether people missed reporting demographics. All of the control variables were pre-registered.

All results reported in this section come from OLS regressions with the aforementioned controls, and we report two-sided p-values without adjustment for multiple comparisons.

3.2.1 Interest in Getting the Vaccine

Supplementary Figure 5 Panels A and C display the mean rating of *Hypothetical Interest in Getting the Vaccine* (Panel A) and *Current Interest in Getting the Vaccine* (Panel C) for each message based on raw data, overlaying on a dot plot. We note that the ratings were on average much lower than ratings observed in our earlier online experiments. This makes sense because this replication experiment recruited participants who still had not gotten vaccinated late April 2021 and were likely to have stronger vaccine hesitancy than participants in our earlier experiments conducted in February 2021.

Supplementary Table 35 Columns 1 and 3 report OLS regression results about *Hypothetical Interest in Getting the Vaccine* (Column 1) and *Current Interest in Getting the Vaccine* (Column 3). Both Columns indicate that adding the ownership language did not have a statistically significant effect at the 5% level (B = 0.04, SE = 0.16, p = 0.80 in Column 1 and B = 0.15, SE = 0.16, p = 0.36 in Column 3). Instead, people overall indicated a greater interest in getting the vaccine after receiving a message containing the video than after receiving a message without the video, regardless of whether we asked about their interest in a hypothetical manner (B = 0.60, SE = 0.16, $p < 0.001$; Column 1) or we asked about their current interest in a more direct manner (B = 0.43, SE = 0.16, p = 0.009; Column 3).

3.2.2 Message Persuasiveness

Supplementary Figure 5 Panels B and D display the mean rating of *Persuasiveness-Hypothetical* (Panel A) and *Persuasiveness-Current* (Panel C) for each message based on raw data, overlaying on a dot plot. Supplementary Table 35 Columns 2 and 4 report OLS regression results about *Persuasiveness-Hypothetical*

(Column 2) and *Persuasiveness-Current* (Column 4). Both columns indicate that adding the ownership language did not have a statistically significant positive main effect at the 5% level (B = 0.07, SE = 0.14, $p = 0.62$ in Column 2 and B = 0.09, SE = 0.14, $p = 0.52$ in Column 4). Instead, people rated the two messages containing the video as significantly more persuasive than the two messages without the video (B = 1.27, SE = 0.14, $p < 0.001$ in Column 2 and B = 1.34, SE = 0.14, $p < 0.001$ in Column 4).

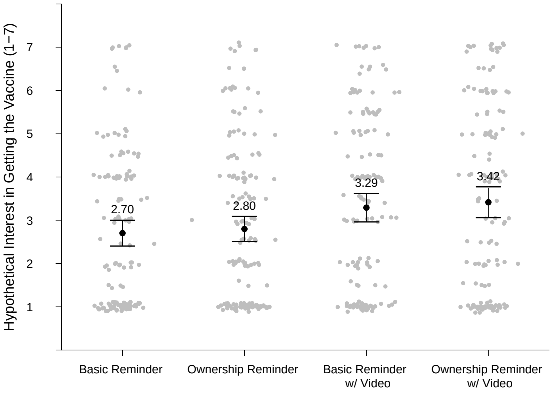

(a) Panel A: Hypothetical Interest in Getting the Vaccine

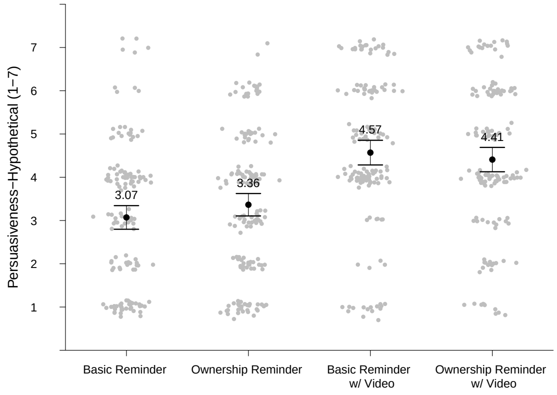

(b) Panel B: Persuasiveness-Hypothetical

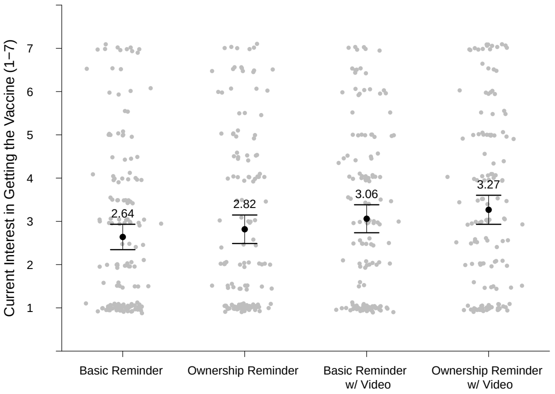

(c) Panel C: Current Interest in Getting the Vaccine

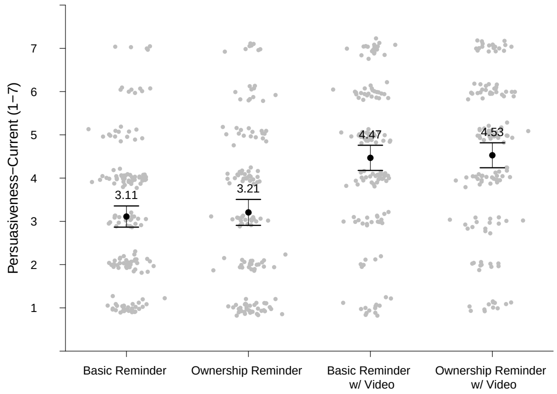

(d) Panel D: Persuasiveness-Current

Figure 5: Interest in Getting the Vaccine and Persuasiveness by Message and Measurement Methods

Figure Legend: The interest in getting the COVID-19 vaccine and message persuasiveness for each condition are depicted here for the online experiment conducted in April 2021 (N=1,178 participants on Amazon Mechanical Turk and Prolific). Gray dots represent individual observations, with jittering in both dimensions for increased visibility, while the black dots represent condition means. Panel A displays participants' interest in getting the vaccine upon receiving the message when they were asked in a hypothetical manner. Panel B displays participants' perceived persuasiveness of the text message when they were asked about their hypothetical interest in the vaccine. Panel C displays participants' interest in getting the vaccine upon receiving the message when they were asked about their current interest. Panel D displays participants' perceived persuasiveness of the text message when they were asked about their current interest in the vaccine. In all panels, error bars represent ±1 standard error of the corresponding mean. For Panels A and B, the number of observations in each condition (from left to right in each panel) is 141, 159, 144, and 142. For Panels C and D, the number of observations in each condition (from left to right in each panel) is 162, 145, 141, and 144.

Table 35: Effects of Text Messages on Interest in Getting the Vaccine and Persuasiveness

	Hypothetical Interest in Getting the Vaccine (1)	Persuasiveness-Hypothetical (2)	Current Interest in Getting the Vaccine (3)	Persuasiveness-Current (4)	Interest in Getting the Vaccine (5)	Persuasiveness (6)
Ownership	0.04	0.07	0.15	0.09	0.09	0.07
	(0.16)	(0.14)	(0.16)	(0.14)	(0.11)	(0.10)
Video	0.60***	1.27***	0.43**	1.34***	0.52***	1.30***
	(0.16)	(0.14)	(0.16)	(0.14)	(0.11)	(0.10)
Gender-Female	0.00	0.14	-0.13	0.08	-0.06	0.11
	(0.16)	(0.14)	(0.17)	(0.15)	(0.12)	(0.10)
Gender-Other	1.09	0.43	1.17	1.17	1.13	0.85
	(1.41)	(1.20)	(0.86)	(0.90)	(0.74)	(0.72)
Age	-0.02*	-0.01	-0.02**	0.01	-0.02***	-0.00
	(0.01)	(0.01)	(0.01)	(0.01)	(0.00)	(0.00)
Hispanic	0.34	0.19	0.06	0.21	0.24	0.20
	(0.36)	(0.30)	(0.48)	(0.42)	(0.29)	(0.24)
Black	0.01	0.32	0.20	0.36	0.09	0.35*
	(0.28)	(0.25)	(0.30)	(0.25)	(0.20)	(0.18)
Asian	0.96**	0.13	0.90**	0.50	0.93***	0.33
	(0.35)	(0.27)	(0.29)	(0.27)	(0.22)	(0.19)
Race-Other/Unknown	0.21	0.29	-0.47	-0.12	-0.17	0.05
	(0.35)	(0.31)	(0.28)	(0.27)	(0.22)	(0.20)
Missing Demographics	-0.82	-0.96	-1.09	-1.89	-0.99	-1.49
	(1.55)	(1.27)	(1.14)	(1.15)	(0.91)	(0.85)
Constant	3.26***	3.25***	3.51***	2.80***	3.38***	3.04***
	(0.31)	(0.29)	(0.32)	(0.29)	(0.22)	(0.21)
N	586	586	592	592	1178	1178
R^2	0.053	0.131	0.063	0.145	0.054	0.135

Legend: The table reports ordinary least squares (OLS) regressions predicting participants' interest in getting the COVID-19 vaccine after receiving the message (Columns 1, 3, and 5) and participants' perceived persuasiveness of the message (Columns 2, 4, and 6). Columns 1 and 2 focus on participants whose interest in the vaccine was solicited in a hypothetical manner. Columns 3 and 4 focus on participants whose current interest in the vaccine was solicited. In Columns 5 and 6 where all participants in this experiment are included, *Interest in the Vaccine* (*Persuasiveness*) equals either *Hypothetical Interest in the Vaccine* (*Persuasiveness-Hypothetical*) or *Current Interest in the Vaccine* (*Persuasiveness-Current*) depending on the questions participants were assigned to answer. *Ownership* is a dummy variable coded as 1 when participants received one of the text messages containing the ownership language. Its p-value is 0.798, 0.622, 0.355, 0.520, 0.405, and 0.486 in Columns 1-6, respectively. *Video* is a dummy variable coded as 1 when participants received one of the text messages containing the video. Its p-value is < 0.001 in Columns 1-2 and 4-6 and is 0.009 in Column 3. Control variables include an indicator for whether a participant was female ("Gender-Female"), an indicator for whether a participant's gender was either other or unknown ("Gender-Other"), participant age in years ("Age"), indicators for whether a participant was Hispanic/Latino ("Hispanic"), Black ("Black"), or Asian ("Asian"), an indicator for whether a participant either had other race or did not report race information ("Race-Other/Unknown"), as well as an indicator for whether participants did not respond to demographic questions ("Missing Demographics"). "Black", "Asian", and "Race-Other/Unknown" were coded as 0 if a participant was Hispanic or Latino. Robust standard errors are reported in parentheses. $^{*}p<0.05$; $^{**}p<0.01$; $^{***}p<0.001$ (all two-sided).

3.2.3 Mechanism for the Ownership Language

Next, we test how our ownership language changed people's psychological ownership of the vaccine. As pre-registered, we used the full sample for this analysis. Supplementary Table 36 reports the results of an OLS regression that predicts *Psychological Ownership* as a function of an indicator for whether people read a text messaging containing the ownership language and the aforementioned control variables. Consistent with the intention behind our design of the ownership language, the ownership language elevated participants' feeling that a COVID-19 vaccine was already theirs (B = 0.39, SE = 0.12, $p < 0.001$).

Table 36: Effects of Ownership Language on Psychological Ownership

Dependent Measure	Psychological Ownership
Ownership	0.39***
	(0.12)
Gender-Female	0.32**
	(0.12)
Gender-Other	0.66
	(0.79)
Age	0.00
	(0.01)
Hispanic	0.17
	(0.28)
Black	0.23
	(0.21)
Asian	0.30
	(0.22)
Race-Other/Unkown	-0.25
	(0.23)
Missing Demographics	-1.45
	(1.46)
Constant	3.33***
	(0.22)
N	1168
R^2	0.021

Legend: The table reports an ordinary least squares (OLS) regression predicting the extent to which the message made participants feel that the COVID-19 vaccine was already theirs. *Ownership* is a dummy variable coded as 1 when participants received one of the text messages containing the ownership language. Its p-value is < 0.001. Control variables include an indicator for whether a participant was female ("Gender-Female"), an indicator for whether a participant's gender was either other or unknown ("Gender-Other"), participant age in years ("Age"), indicators for whether a participant was Hispanic/Latino ("Hispanic"), Black ("Black"), or Asian ("Asian"), an indicator for whether a participant either had other race or did not report race information ("Race-Other/Unknown"), as well as an indicator for whether participants did not respond to demographic questions ("Missing Demographics"). "Black", "Asian", and "Race-Other/Unknown" were coded as 0 if a participant was Hispanic or Latino. Robust standard errors are reported in parentheses. $^{*}p<0.05$; $^{**}p<0.01$; $^{***}p<0.001$ (all two-sided).

3.2.4 Mechanisms for the Video

We next examine how the video may change people's beliefs and perceptions about COVID-19 and the vaccine. As pre-registered, we used the full sample for this analysis. Supplementary Table 37 Columns 1-6 report the results of OLS regressions that predict each belief or perception measure as a function of an indicator for whether people read one of the text messages containing the video and the aforementioned control variables. As shown in Column 1, our video significantly increased people's predicted likelihood of getting COVID-19 without the vaccine by 6.25 percentage points (B = 6.25, SE = 1.42, $p < 0.001$), suggesting that the video made people believe COVID-19 is more prevalent and easier to transmit. As shown in Column 2, *Infection Likelihood with Vaccine* did not significantly differ between people who were assigned to watch the video and those who were not assigned to watch the video at the 5% level (B = -1.96, SE = 1.29, p = 0.13). Furthermore, Column 3 shows that *Vaccine Effectiveness*—which we calculated by subtracting *Infection Likelihood with Vaccine* from *Infection Likelihood without Vaccine*—was significantly higher by 8.22 percentage points in the two conditions involving the video than in the other two conditions (B = 8.22, SE = 1.59, $p < 0.001$). This suggests that watching the video boosted people's belief that the vaccine could reduce their infection likelihood.

In addition, watching the video led participants to feel more worried about the possibility of transmitting COVID-19 to others (B = 0.36, SE = 0.12, p = 0.002; Column 4) and anticipated greater regret of not getting vaccinated (B = 0.36, SE = 0.13, p = 0.006; Column 5). Similar to our previous online experiments, our video did not significantly change trust in the safety and development process of the authorized vaccines at the 5% level (B = 0.09, SE = 0.09, p = 0.34; Column 6).

We also conducted a mediation analysis to explore which of these beliefs and perceptions drove the effect of the video on interest in getting the vaccine. Since our results are similar between participants who responded to vaccination intention measures in a hypothetical manner and those who reported their current interest in getting the vaccine, we used the full sample for the mediation analysis. We ran a multi-mediator model[54] that included *Infection Likelihood without Vaccine*, *Vaccine Effectiveness*, *Worry of Transmission*, *Anticipated Regret*, and *Trust in Vaccine* as potential mediators and kept the aforementioned demographic controls. We used 5,000 bootstrapped samples. We estimated a positive indirect effect of *Infection Likelihood without Vaccine* (B = 0.03, SE = 0.01), and the 95% bias-corrected CI excluded 0 ([0.005, 0.06]). We also estimated a positive indirect effect of *Worry of Transmission* (B = 0.05, SE = 0.02), and the 95% bias-corrected CI excluded 0 ([0.02, 0.10]). In addition, we estimated a positive indirect effect of *Anticipated Regret* (B = 0.15, SE = 0.05), and the 95% bias-corrected CI excluded 0 ([0.05, 0.26]). These results suggest that in this replication experiment, watching the video significantly boosted people's interest in getting the vaccine by enhancing their beliefs in their infection likelihood without the vaccine, elevating their worry of transmission to others, and increasing their anticipated regret of not getting vaccinated.

Table 37: Effects of Video on Beliefs and Perceptions

Dependent Measure	Infection Likelihood w/o Vaccine	Infection Likelihood w/ Vaccine	Vaccine Effectiveness	Worry	Anticipated Regret	Trust
	(1)	(2)	(3)	(4)	(5)	(6)
Video	6.25***	-1.96	8.22***	0.36**	0.36**	0.09
	(1.42)	(1.29)	(1.59)	(0.12)	(0.13)	(0.09)
Gender-Female	8.08***	6.08***	2.00	0.38**	0.16	-0.45***
	(1.43)	(1.33)	(1.63)	(0.12)	(0.13)	(0.10)
Gender-Other	5.75	-10.19**	15.95*	0.66	0.96	1.50*
	(7.31)	(3.26)	(6.87)	(0.68)	(0.71)	(0.65)
Age	-0.24***	0.00	-0.25***	-0.03***	-0.01*	-0.02***
	(0.06)	(0.06)	(0.07)	(0.00)	(0.01)	(0.00)
Hispanic	4.74	7.28*	-2.55	0.09	0.02	-0.02
	(3.57)	(3.50)	(4.69)	(0.29)	(0.31)	(0.22)
Black	2.10	7.37**	-5.27*	0.42	0.45*	-0.16
	(2.41)	(2.29)	(2.43)	(0.22)	(0.22)	(0.14)
Asian	7.45*	-6.26**	13.70***	0.34	1.22***	0.65***
	(3.02)	(2.16)	(3.05)	(0.24)	(0.25)	(0.19)
Race-Other/Unkown	-5.32	1.64	-6.96	-0.30	-0.01	0.00
	(2.79)	(2.76)	(3.65)	(0.24)	(0.26)	(0.20)
Missing Demographics	-5.86	9.65	-15.51	-1.58	-0.88	-1.79
	(10.76)	(8.43)	(9.30)	(0.84)	(1.01)	(1.02)
Constant	40.15***	21.00***	19.15***	4.08***	3.62***	3.83***
	(2.69)	(2.46)	(3.19)	(0.21)	(0.24)	(0.18)
N	1172	1172	1172	1172	1172	1169
R^2	0.065	0.041	0.059	0.050	0.034	0.060

Legend: The table reports ordinary least squares (OLS) regressions predicting *Infection Likelihood without Vaccine* in Column 1, *Infection Likelihood with Vaccine* in Column 2, *Vaccine Effectiveness* in Column 3, *Worry of Transmission* in Column 4, *Anticipated Regret* in Column 5, and *Trust in Vaccine* in Column 6. We multiplied participants' original responses by 100 for *Infection Likelihood without Vaccine* (ranging from 0 to 100), *Infection Likelihood with Vaccine* (ranging from 0 to 100), and *Vaccine Effectiveness* (ranging from -100 to 100). *Video* is a dummy variable coded as 1 when participants received one of the text messages containing the video. Its p-value is < 0.001, 0.128, < 0.001, 0.002, 0.006, and 0.335 in Columns 1-6, respectively. Control variables include an indicator for whether a participant was female ("Gender-Female"), an indicator for whether a participant's gender was either other or unknown ("Gender-Other"), participant age in years ("Age"), indicators for whether a participant was Hispanic/Latino ("Hispanic"), Black ("Black"), or Asian ("Asian"), an indicator for whether a participant either had other race or did not report race information ("Race-Other/Unknown"), as well as an indicator for whether participants did not respond to demographic questions ("Missing Demographics"). "Black", "Asian", and "Race-Other/Unknown" were coded as 0 if a participant was Hispanic or Latino. Robust standard errors are reported in parentheses. *p<0.05; **p<0.01; ***p<0.001 (all two-sided).

3.2.5 Heterogeneous Treatment Effects

Finally, as pre-registered, we explored how the effects of our interventions on vaccination intentions and persuasiveness differed across the different sub-populations, including participants who identified themselves as Democrats or Republicans, participants who did or did not receive the flu shot at least once during the two most recent seasons, participants with or without a bachelor's degree, participants who self-reported washing their hands at a high or low frequency when coming back home from outside, participants who self-reported wearing masks outside at a high or low frequency, and participants with or without children under 18. For the frequency with which people washed their hands when coming back home and the frequency with which people wore masks when outside, we pre-registered that we would do a median split. The median response is 5 for both questions, corresponding to "Always or almost always." Thus, we essentially separated participants into two buckets for each question based on whether or not they reported always washing their hands (or always wearing masks).

Since (1) our results are qualitatively similar between participants who responded to intention measures in a hypothetical manner and those who reported their current interest in getting the vaccine and (2) detecting differences between sub-populations requires a large sample, we used the full sample to explore the heterogeneous treatment effects.

Given that we largely did not see significant differences between sub-populations at the 5% level, we only report here heterogeneous treatment effects based on political affiliations, which may be of the most interest to readers (Supplementary Table 38). Results about other sub-sample analyses are available at https://osf.io/qn8hr/?view_only=cf7b2bc590054aee8c4a2bae99ef20c5.

Table 38: Effects of Text Messages on Interest in Getting the Vaccine and Persuasiveness for Democrats and Republicans

Dependent Measure	Interest in Getting the Vaccine			Persuasiveness		
Sample	Democrat	Republican	Both	Democrat	Republican	Both
	(1)	(2)	(3)	(4)	(5)	(6)
Ownership	0.07	0.00	0.04	0.17	-0.13	-0.10
	(0.18)	(0.15)	(0.15)	(0.15)	(0.16)	(0.16)
Video	0.69***	0.35*	0.34*	1.65***	0.99***	0.99***
	(0.18)	(0.15)	(0.15)	(0.14)	(0.16)	(0.16)
Democrat			1.77***			0.62***
			(0.20)			(0.18)
Ownership x Democrat			0.06			0.29
			(0.23)			(0.21)
Video x Democrat			0.35			0.64**
			(0.23)			(0.21)
Gender-Female	-0.04	-0.11	-0.09	-0.04	0.23	0.08
	(0.18)	(0.15)	(0.12)	(0.15)	(0.16)	(0.11)
Gender-Other	1.20*		1.26*	1.19		1.29*
	(0.53)		(0.56)	(0.62)		(0.63)
Age	-0.02	0.00	-0.01	0.00	0.01*	0.01
	(0.01)	(0.01)	(0.01)	(0.01)	(0.01)	(0.00)
Hispanic	-0.52	0.40	-0.20	-0.25	0.77	0.07
	(0.44)	(0.50)	(0.35)	(0.30)	(0.53)	(0.28)
Black	-1.05***	1.09	-0.77**	-0.28	0.81	-0.14
	(0.25)	(0.67)	(0.25)	(0.21)	(0.68)	(0.21)
Asian	0.29	0.46	0.41	-0.08	0.20	0.04
	(0.26)	(0.46)	(0.22)	(0.23)	(0.38)	(0.19)
Race-Other/Unknown	-0.33	-0.21	-0.27	0.25	0.01	0.12
	(0.39)	(0.24)	(0.22)	(0.29)	(0.30)	(0.21)
Missing Demographics	0.00	0.00	0.00	0.00	0.00	0.00
	(.)	(.)	(.)	(.)	(.)	(.)
Constant	4.51***	1.95***	2.34***	3.55***	2.25***	2.59***
	(0.38)	(0.26)	(0.24)	(0.32)	(0.32)	(0.25)
N	480	442	922	480	442	922
R^2	0.088	0.030	0.267	0.225	0.106	0.234

Legend: The table reports ordinary least squares (OLS) regressions predicting participants' interest in getting the COVID-19 vaccine after receiving the message (Columns 1-3) and their perceived persuasiveness of the text message (Columns 4-6), broken down by political affiliation. *Interest in the Vaccine* (*Persuasiveness*) equals either *Hypothetical Interest in the Vaccine* (*Persuasiveness-Hypothetical*) or *Current Interest in the Vaccine* (*Persuasiveness-Current*) depending on the questions participants were assigned to answer. Columns 1 and 4 focus on participants who self-identified as Democrats, Columns 2 and 5 focus on participants who self-identified as Republicans, and Columns 3 and 6 focus on the combined sample (excluding people who did not report identifying with either party). *Ownership* is a dummy variable coded as 1 when participants received one of the text messages containing the ownership language. Its p-value is 0.699, 0.991, 0.789, 0.251, 0.416, and 0.521 in Columns 1-6, respectively. *Video* is a dummy variable coded as 1 when participants received one of the text messages containing the video. Its p-value is < 0.001 in Column 1, 0.020 in Column 2, and 0.025 in Column 3, and < 0.001 in Columns 4-6. The interaction between *Ownership* and *Democrat* has a p-value of 0.788 in Column 3 and 0.171 in Column 6. The interaction between *Video* and *Democrat* has a p-value of 0.136 in Column 3 and 0.003 in Column 6. Control variables include an indicator for whether a participant was female ("Gender-Female"), an indicator for whether a participant's gender was either other or unknown ("Gender-Other"), participant age in years ("Age"), indicators for whether a participant was Hispanic/Latino ("Hispanic"), Black ("Black"), or Asian ("Asian"), an indicator for whether a participant either had other race or did not report race information ("Race-Other/Unknown"), as well as an indicator for whether participants did not respond to demographic questions ("Missing Demographics"). "Black", "Asian", and "Race-Other/Unknown" were coded as 0 if a participant was Hispanic or Latino. Robust standard errors are reported in parentheses. *p<0.05; **p<0.01; ***p<0.001 (all two-sided).

4 Comparing the First RCT with Online Experiments

The Extended Data Table 6 summarizes the differences between the first RCT and online experiments in their measures and sample characteristics, which may contribute to the discrepancy in findings between our online and field studies among other reasons. Also, to understand whether our failure to detect statistical significant effects of a given intervention in a given setting reflects a statistical power issue, and to quantify the discrepancy between our field and online studies, we report the 95% confidence interval, Cohen's d (or Cohen's h), and η_p^2 of each estimated effect and compare the patterns between field and online studies. For simplicity and for greater statistical power that could give us more precise estimates, we pooled all online experiments into a sample. Supplementary Table 39 shows the OLS regressions predicting vaccination intentions across participants in the three online experiments. We provide our interpretations of Extended Data Table 6 below.

In the online experiments, the point estimate of the ownership intervention is -0.06, and its 95% confidence interval excludes that the ownership intervention increased interest in getting the vaccine by 0.10 on a seven-point scale (i.e, the upper bound of the 95% confidence interval), which is less than one third of the increase in vaccination interest that the video intervention was estimated to generate (i.e., 0.38). The Cohen's d and η_p^2 for the ownership intervention are 0.02 and 0.0002, respectively. In comparison, the Cohen's d and η_p^2 for the video intervention are larger (0.16 and 0.007, respectively).

In the first RCT, when the outcome variable captures appointments at UCLA Health, the point estimate of the video intervention is -0.003, and the 95% confidence interval excludes that the video intervention increased appointment rates by 0.2 percentage points (i.e, the upper bound of the 95% confidence interval), which is less than one seventh of the increase in appointment rates that the ownership intervention was estimated to generate (i.e., 1.5 percentage points). The Cohen's h and η_p^2 for the video intervention are 0.007 and 0.00002, respectively. In comparison, the Cohen's h and η_p^2 for the ownership intervention are larger (0.046 and 0.0005, respectively).

Similarly, in the first RCT, when the outcome variable captures vaccinations at UCLA Health, the point estimate of the video intervention is -0.002, and the 95% confidence interval excludes that the video intervention increased vaccinations rates by 0.3 percentage points (i.e, the upper bound of the 95% confidence interval), which is less than one third of the increase in vaccination rates that the ownership intervention was estimated to generate (i.e., 1.1 percentage points). The Cohen's h and η_p^2 for the video intervention are 0.004 and $9.1 * e^{-06}$, respectively. In comparison, the Cohen's h and η_p^2 for the ownership intervention are larger (0.031 and 0.0002, respectively). Altogether, these results suggest that our online experiments have qualitatively different patterns from the first RCT regarding the effects of ownership and video interventions.

Table 39: Effects of Text Messages on Vaccination Intentions Pooled Across Online Experiments

	Vaccination Intentions
Ownership	-0.06
	(0.08)
Video	0.38***
	(0.08)
Gender-Female	-0.07
	(0.08)
Gender-Other	1.15**
	(0.39)
Age	-0.01*
	(0.00)
Hispanic	0.14
	(0.20)
Black	-0.38*
	(0.15)
Asian	1.08***
	(0.12)
Race-Other/Unknown	-0.17
	(0.18)
Missing Demographics	-0.95
	(0.57)
Constant	4.33***
	(0.16)
N	3181
R^2	0.034

Legend: The table reports ordinary least squares (OLS) regressions predicting participants' intention to get the COVID-19 vaccine after receiving the message across the three online experiments. For two experiments conducted in February 2021, vaccination intentions was measured as "How likely would you be to schedule a vaccination appointment after receiving this message from your healthcare provider?" (from 1 = Not at all Likely to 7 = Very Likely). For the experiment conducted in April 2021, vaccination intentions was measured as the average of participants' responses to two questions: (1) "How likely would you be to schedule a vaccination appointment after receiving this message from your healthcare provider?" (or "How likely are you to schedule a vaccination appointment today after reading this message from your healthcare provider?"; from 1 = Not at all likely to 7 = Very likely) and (2) "How much would you want the vaccine after receiving this message from your healthcare provider?" (or "How much do you want the vaccine now after receiving this message from your healthcare provider?"; from 1 = Not at all to 7 = Very much). *Ownership* is a dummy variable coded as 1 when participants received one of the text messages containing the ownership language. Its p-value is 0.437. *Video* is a dummy variable coded as 1 when participants received one of the text messages containing the video. Its p-value is < 0.001. Control variables include an indicator for whether a participant was female ("Gender-Female"), an indicator for whether a participant's gender was either other or unknown ("Gender-Other"), participant age in years ("Age"), indicators for whether a participant was Hispanic/Latino ("Hispanic"), Black ("Black"), or Asian ("Asian"), an indicator for whether a participant either had other race or did not report race information ("Race-Other/Unknown"), as well as an indicator for whether participants did not respond to demographic questions ("Missing Demographics"). "Black", "Asian", and "Race-Other/Unknown" were coded as 0 if a participant was Hispanic or Latino. Robust standard errors are reported in parentheses. *p<0.05; **p<0.01; ***p<0.001 (all two-sided).

5 Online Survey Comparing Beliefs and Perceptions Across People with Different Vaccination Intentions

We conducted an online survey in January 2021 to explore factors predicting the general public's vaccination intentions.

5.1 Method

5.1.1 Participants

Given that our RCTs targeted patients at UCLA Health, most of whom live in California, we aimed to recruit 500 survey respondents living in California. To speed up data collection and get a relatively more diverse sample, we recruited survey respondents from both MTurk and Prolific and used these platforms' location screening tools to identify California residents. Our final sample consists of 515 participants (48.93% female, $M_{age} = 33.91$). If a person took the survey more than once, we kept their first complete response.

5.1.2 Procedure and Measures

At the introduction, we informed participants that they should consider the RNA vaccines developed by Pfizer-BioNTech and Moderna (both of which had been authorized by the FDA for emergency use) when answering questions about COVID-19 vaccines during the survey. We collected participants' vaccination intentions, beliefs, and perceptions about COVID-19, attitudes toward the authorized COVID-19 vaccines, general preventative measures, and demographics. The complete survey is available at https://osf.io/qn8hr/?view_only=cf7b2bc590054aee8c4a2bae99ef20c5.

- *Vaccination Intentions*: Following a popular national survey conducted by Pew Research Center[41], we measured participants' vaccination intentions by asking, "If one of the COVID-19 vaccines were available to you today, would you get the vaccine?" Participants chose one from four options: "Definitely would get the vaccine," "Probably would get the vaccine," "Probably would not get the vaccine," and "Definitely would not get the vaccine." We asked participants to explain why they were unsure about whether to get the vaccine (if they indicated they probably would or would not get it) or why they would definitely not get the vaccine (if they indicated they would definitely not get it).

- *Prediction about Others' Intentions*: We told participants that a large sample of US adults were recently surveyed about their vaccination intentions. We asked participants to predict the percentage of U.S. adults who indicated that they would definitely or probably get the vaccine (from 0% to 100%).

- *Beliefs about Herd Immunity*: Participants indicated the percentage of Americans they thought need to get the COVID-19 vaccine in order to have society protected by herd immunity (from 0% to 100%). Note that the order in which participants reported the three aforementioned questions (about their vaccination intentions, others' intentions, and herd immunity) versus the following questions about their beliefs and perceptions about COVID-19 and the authorized vaccines were counterbalanced. The order did not make a meaningful difference and thus will not be discussed.

- *Infection Likelihood* and *Vaccine Effectiveness*: We asked participants to report their likelihood of getting infected with COVID-19 if they did not get the COVID-19 vaccine (*Infection Likelihood without*

Vaccine) and if they got the COVID-19 vaccine (*Infection Likelihood with Vaccine*) (from 0% = I certainly won't get infected with COVID-19 to 100% = I certainly will get infected with COVID-19). For each participant, we subtracted *Infection Likelihood with Vaccine* from *Infection Likelihood without Vaccine* to capture her belief in the effectiveness of the COVID-19 vaccine in reducing her chance of getting infected with the COVID-19 (*Vaccine Effectiveness*). The value of *Vaccine Effectiveness* in theory could range from -100% to 100%.

- *Infection Severity*: We used two measures to capture people's beliefs in the severity of their symptoms if they got infected with COVID-19: "In the unfortunate scenario you became infected with COVID-19, how ill do you think you would be?" (from 0 = No symptoms to 10 = Severely ill/hospitalized) and "In the unfortunate scenario you became infected with COVID-19, how confident are you that you can fully recover from it?" (from 0 = Not at all confident to 10 = Extremely confident). Participants' responses to these two questions were significantly correlated ($r = -0.59$, $p < 0.0001$) and thus were averaged (after the second item was reverse coded) to form a composite score of *Infection Severity*.

- *Vulnerability*: Participants rated the extent to which they agreed with the statement, "Without getting the COVID-19 vaccine, I would feel very vulnerable to COVID-19" (from 0 = Strongly disagree to 10 = Strongly agree).

- *Fear of Infections*: Participants rated the extent to which they agreed with the statement, "I am scared of getting COVID-19" (from 0 = Strongly disagree to 10 = Strongly agree).

- *Worry of Transmission*: Participants rated the extent to which they agreed with the statement, "The possibility of passing COVID-19 to other people worries me" (from 0 = Strongly disagree to 10 = Strongly agree).

- *Anticipated Regret*: Participants rated the extent to which they agreed with the statement, "I would regret not getting a COVID-19 vaccine if I end up getting infected with COVID-19" (from 0 = Strongly disagree to 10 = Strongly agree).

- *Trust in Vaccine*: We measured trust in the safety and development process of the vaccine by asking participants to rate their agreement with seven statements (from 0 = Strongly disagree to 10 = Strongly agree). The statements captured worries about side effects ("I am concerned about potential side effects of the COVID-19 vaccine"), the possibility of getting COVID-19 from the vaccine ("I worry that the COVID-19 vaccine will lead me to be infected with COVID-19"), and potential negative long-term impacts of the vaccine ("I worry that the COVID-19 vaccine will have long-term negative impacts on my health"). The statements also captured concerns about the speed of the vaccine authorization process ("I am concerned that the current COVID-19 vaccines were approved too quickly"), overall trust in the development of the vaccine ("I trust the research and development process of COVID-19 vaccines"), perceived safety of the vaccine ("I am confident that the research and development process of COVID-19 vaccine has produced a safe vaccine"), and hesitancy about the vaccine ("Before feeling comfortable to get the COVID-19 vaccine myself, I would want to wait for enough people to get the vaccine."). Participants' responses to these seven questions reached a high inter-item reliability (Cronbach's alpha = 0.91); thus, we averaged responses to these questions (after reverse coding responses to the negatively framed statements) to form a composite score of *Trust in Vaccine*. The higher one's composite score is,

the more they trusted the safety of vaccine and its development process. Regarding the statement that intended to capture participants' concern about the speed of FDA's approval, we realized in hindsight that we should have asked whether people were concerned that the vaccines were *authorized for use*, rather than *approved*, too quickly. Since this item was highly correlated with the other six items and the general population may not know the difference between FDA's approval and FDA's emergency use authorization, we kept this item. We corrected this mistake in the same measure for our online experiments, as reported in Section 3.

- *Vaccination Importance*: To evaluate how important getting vaccinated was to participants, we had them indicate agreement with four statements (from 0 = Strongly disagree to 10 = Strongly agree): (1) "Getting the COVID-19 vaccine is essential for my life to go back to normal"; (2) "Having enough people get the COVID-19 vaccine is essential for society to go back to normal"; (3) "Getting the COVID-19 vaccine is important for protecting myself"; and (4) "Getting the COVID-19 vaccine is important for protecting others around me." Participants' responses to these four questions reached a high inter-item reliability (Cronbach's alpha = 0.93); thus, we averaged responses to these questions to form a composite score of *Vaccination Importance*. The higher one's composite score is, the more they viewed vaccination as important for themselves and the society.

- *Prevalence of COVID-19*: To assess participants' beliefs in the prevalence of COVID-19, we asked, "How many Americans do you think, on average, test positive for COVID-19 every day in January 2021?" and "How many Americans do you think, on average, die from COVID-19 every day in January 2021?" For both questions, participants entered a number.

- *Personal Experience with COVID-19*: Participants reported whether they had ever been tested for COVID-19 and if yes, whether they had ever tested positive. Those who tested positive were further asked about what happened to them.

- *Demographics*: Participants reported their age, gender, race/ethnicity, highest level of education completed, pre-tax household income in 2020, and the state they were currently living in. Since 98% of participants reported that they indeed were living in California, we did not exclude participants based on their state of residence. Participants also reported the political party they identified the most with ("The Democratic Party", "The Republican Party", "I am independent", and "Other") and those who chose "I am independent" were further asked to indicate if they identified relatively more with the Democrat Party or the Republican Party or if they identified equally with both parties. We treat people who identified more with the Democratic (Republican) Party (regardless of whether they initially chose "I am independent") as Democrats (Republicans).

- *Second-hand Experience with COVID-19*: Participants reported if they personally knew anyone who was hospitalized or died as a result of COVID-19 and if they had ever been in close contact with someone who had COVID-19 at the time of the contact.

- *Familiarity with Authorized Vaccines*: Participants reported their familiarity for each of the authorized vaccines at the time the study was conducted (i.e., the Pfizer-BioNTec COVID-19 vaccine and the Moderna COVID-19 vaccine).

- *Preventative Measures*: Participants rated how often they wore a mask when going outside as well as how often they washed their hands when coming home from outside (from 1 = Never or almost never to 5 = Always or almost always). They also reported whether they got the flu shot in the current (2020-2021) flu season.

5.2 Results

Descriptive Statistics. We first report summary statistics about participants' vaccination intentions, predictions about others' intentions, belief about herd community, general familiarity with the vaccine, and experiences with COVID-19. In our sample, 51.84% of participants reported that they definitely would get the vaccine if it was available to them, 28.93% reported that they probably would get the vaccine, 11.07% probably would not get the vaccine, and the remaining 8.16% definitely would not get it.

Consistent with results of national surveys among US adults[36,41], we found that Democrats reported stronger intentions to get the vaccine than Republicans and that both Black and Hispanic/Latino participants indicated weaker intentions than White (excluding Hispanic or Latino) participants (see Supplementary Table 40). We conducted an OLS regression with robust standard errors to predict intent to get vaccinated (1 = definitely yes, 2 = probably yes, 3 = probably no, 4 = definitely no) as a function of whether a participant identified with the Democrat Party, the Republican Party (as the reference group), or neither. Note that a lower value of the outcome variable indicates stronger vaccination intentions. The difference between Democrats and Republicans was statistically significant (difference = -0.84, SE = 0.13, $p < 0.001$), suggesting that Democrats in our sample reported stronger vaccination intentions than Republicans. We also ran an OLS regression with robust standard errors to predict vaccination intentions as a function of whether a participant was Hispanic/Latino, Black (excluding Hispanic or Latino), Asian (excluding Hispanic or Latino), other race, or White (excluding Hispanic or Latino; as the reference group). The regression showed that Hispanic and Black (excluding Hispanic or Latino) participants reported weaker vaccination intentions than White (excluding Hispanic or Latino) participants (for Hispanic participants: B = 0.36, SE = 0.17, p = 0.03; for Black participants: B = 0.43, SE = 0.21, p = 0.04). The results are robust if we use OLS or logistics regressions to predict a binary indicator for whether or not participants definitely would get the vaccine. Nevertheless, we note that we sample had a small number of Hispanic and Black participants.

On average, participants predicted that 62.5% of American adults, when surveyed about their vaccination intentions, indicated they would definitely or probably get the vaccine (SD = 15.36%). This prediction is fairly accurate, relative to the actual percentage of American adults who indicated definitely or probably yes when surveyed by the Pew Research Center[41] at the end of 2020. Participants on average estimated that 76% of people would need to get vaccinated to reach herd immunity (SD = 20.52%). Participants tended to underestimate the prevalence and severity of COVID-19 infections. Specifically, among our survey respondents, the median estimate of daily confirmed cases in the U.S. in January 2021 was 30,000, and the median estimate of daily deaths caused by COVID-19 in the U.S. in January 2021 was 2,000; both were much lower than the actual numbers. According to https://covid.cdc.gov/covid-data-tracker/#trends_dailytrendscases, the average daily number of COVID-19 cases and deaths in the US reported to the Centers for Disease Control and Prevention was 233,181 and 3,002, respectively, during January 1-January 12, 2021 (the day prior to our survey).

People were similarly familiar with the Pfizer-BioNTech and Moderna vaccines: 7.06% of participants

Table 40: Vaccination Intentions Overall and by Political Affiliation and Race/Ethnicity

Vaccination Intentions	Full Sample	Democrats	Republicans	Other Political Orientation	
Definitely Yes	51.84%	62.19%	20.31%	31.40%	
Probably Yes	28.93%	27.12%	43.75%	25.58%	
Probably No	11.07%	8.22%	15.63%	19.77%	
Definitely No	8.16%	2.47%	20.31%	23.26%	
Sample	515	365	64	86	
Vaccination Intentions	White	Hispanic	Black	Asian	Other Race
Definitely Yes	56.56%	35.56%	21.05%	52.22%	56.00%
Probably Yes	23.98%	31.11%	52.63%	33.33%	24.00%
Probably No	9.50%	22.22%	15.79%	9.44%	12.00%
Definitely No	9.95%	11.11%	10.05%	5.00%	4.00%
Sample	221	45	19	230	50

Legend: The table reports the percentage of participants who chose "Definitely would get the vaccine" (*Definitely Yes*), "Probably would get the vaccine" (*Probably Yes*), "Probably would not get the vaccine (*Probably No*)", and "Definitely would not get the vaccine" (*Definitely No*) among the full sample of survey respondents as well as broken down by self-reported political affiliation (Democrats, Republicans, or other political orientation) and Race/Ethnicity (White-excluding Hispanic or Latino, Hispanic/Latino, Black-excluding Hispanic or Latino, Asian-excluding Hispanic or Latino, and other race-excluding Hispanic or Latino).

had not heard about the Pfizer-BioNTech vaccine, 53.53% had heard about it but had not read articles and information about it closely, 38.24% had closely read articles and information about it, and 1.18% had personal experiences with it (e.g., had received the vaccine or administered it to others as a healthcare worker); the choice share for the Moderna vaccine was 6.67%, 58.04%, 33.73%, and 1.57%, respectively.

Most (68.62%) of participants had not been tested for COVID-19. Among those who had been tested, 7.5% tested positive. About 30% of participants knew someone who were hospitalized or died as a result of COVID-19, and 13% had ever been in close contact with someone who had COVID-19 at the time of the contact.

Beliefs and Perceptions. Next, we compare beliefs and perceptions about COVID-19 and the authorized vaccines across participants based on whether they definitely would get the vaccine, were uncertain, or definitely would not get the vaccine. Supplementary Table 41 represents the mean and standard deviation of each measure for each of the three groups of participants. Given that we were primarily interested in developing a video to shift the intentions of people who were uncertain, we focus on performing statistical tests (two-sided two-sample student t-tests with equal variances) to compare people definitely would get the vaccine with those who were uncertain. Compared to those who were uncertain, people who definitely would get the vaccine believed they were more likely to get infected with COVID-19 if they did not get the vaccine and they would be less likely to get infected with COVID-19 if they got the vaccine, believed more strongly in the vaccine's effectiveness, felt more vulnerable to COVID-19, were more scared of getting infected, were more worried about transmitting the virus to others, anticipated feeling a greater degree of regret if they passed on the opportunity to get vaccinated and ended up being infected, and trusted the

Table 41: Comparisons of COVID-19-related Beliefs and Perceptions by Vaccination Intentions

Measure	Definitely Yes	Uncertain	Definitely No	Definitely Yes vs. Uncertain (p-value of two-sided *t*-test and Cohen's *d*)
Infection Likelihood w/o Vaccine	57.47% (24.61%)	48.14% (24.97%)	30.78% (26.93%)	p<0.0001, d=0.38
Infection Likelihood w/ Vaccine	16.11% (20.75%)	19.91% (20.16%)	37.60% (31.94%)	p=0.047, d=0.19
Vaccine Effectiveness	41.36% (29.13%)	28.24% (25.35%)	-6.81% (36.12%)	p<0.0001, d=0.48
Infection Severity	4.92 (2.14)	4.61 (2.16)	2.95 (2.32)	p=0.12, d=0.14
Vulnerability	8.13 (2.20)	6.26 (2.78)	2.21 (3.12)	p<0.0001, d=0.76
Fear of Infections	7.51 (2.62)	6.47 (2.97)	2.90 (3.53)	p<0.0001, d=0.37
Worry of Transmission	8.61 (2.00)	7.69 (2.67)	2.83 (3.52)	p<0.0001, d=0.40
Anticipated Regret	9.10 (1.82)	7.21 (2.69)	1.26 (2.46)	p<0.0001, d=0.85
Trust in Vaccine	8.46 (1.61)	5.56 (1.83)	2.74 (1.45)	p<0.0001, d=1.70

Legend: The table reports the mean value and standard deviation (in parentheses) for *Infection Likelihood without Vaccine*, *Infection Likelihood with Vaccine*, *Vaccine Effectiveness*, *Infection Severity*, *Vulnerability*, *Fear of Infections*, *Worry of Transmission*, *Anticipated Regret*, and *Trust in Vaccine* for participants who chose "Definitely would get the vaccine" (*Definitely Yes*), participants who chose either "Probably would get the vaccine" or "Probably would not get the vaccine" (*Uncertain*), and participants who chose "Definitely would not get the vaccine" (*Definitely No*). The scale of *Infection Likelihood without Vaccine* and *Infection Likelihood with Vaccine* is 0% to 100%, and The scale of *Vaccine Effectiveness* is -100% and 100%. The scale of other variables are 0-10. The last column reports the p-value of two-sided two-sample *t*-test that compares each variable between "Definitely Yes" participants and "Uncertain" participants, along with the corresponding Cohen's *d*. Degree of freedom is 469 for the two-sided two-sample *t*-test for all variables except for "Trust in Vaccine" (whose *t*-test has a degree of freedom of 467).

safety and development process of the vaccine more. The two groups of participants did not differ in their beliefs about how severe their symptoms would be if they got infected with COVID-19.

The COVID-19 Video. Based on these results, we decided to create a video with two major components. We aimed to first highlight the threat of COVID-19 (i.e., illustrating the problem) and then immediately propose vaccination as an easy solution. To that end, the first part of the video highlights the prevalence of COVID-19 (e.g., by presenting the number of confirmed cases in Los Angeles and worldwide) and the virus's ease of transmission. Our goal was to make viewers feel that their chance of being infected is high, correcting potential misconceptions about their likelihood of getting infected and transmitting the virus to others, and increasing feelings of vulnerability and worry of spreading the virus. The second part of the video presents vaccination as an easy solution by reassuring viewers about the safety and effectiveness of the authorized vaccines. Specifically, we explain that the authorized vaccines have gone through rigorous evaluations after large-scale clinical trials involving volunteers of all genders/ethnicities/ages/occupations and that these trials have found that the authorized vaccines can reduce symptomatic COVID-19 by up to 95%. The goal of this portion of the video is to shift beliefs about the effectiveness of the vaccine and instill a sense of trust in the vaccine. In addition, the solution part of our video intends to elicit anticipated regret by truthfully telling viewers that 80% of California residents in a recent survey said they would regret not being vaccinated if they ended up getting infected. The number 80% came from this survey where we found that 78.56% of participants gave a rating above the midpoint of the scale in response to the question about anticipated regret.

Supplementary References

50. Dmitrienko, A., Offen, W. W. & Westfall, P. H. Gatekeeping strategies for clinical trials that do not require all primary effects to be significant. *Stat. Med.* **22**, 2387–2400 (2003).
51. Caserotti, M. *et al.* Associations of COVID-19 risk perception with vaccine hesitancy over time for Italian residents. *Soc. Sci. Med.* **272**, 113688 (2021).
52. Levay, K. E., Freese, J. & Druckman, J. N. The demographic and political composition of mechanical turk samples. *SAGE Open* **6**, 2158244016636433 (2016).
53. Pedersen, M. J. & Favero, N. Social distancing during the COVID-19 pandemic: Who are the present and future non-compliers? *Public Adm. Rev.* **80**, (2020).
54. Hayes, A. F. Introduction to mediation, moderation, and conditional process analysis: A regression-based approach. New York, NY: Guilford Press (2017).
