# Supplementary material for: Behavioural nudges increase COVID-19 vaccinations
Source: Nature. 2021 Aug 2;597(7876):404–9. doi: 10.1038/s41586-021-03843-2 (PMC8443442; doi:10.1038/s41586-021-03843-2)
Supplement: Supplementary file 2 — Reporting Summary [file 41586_2021_3843_MOESM2_ESM.pdf]

nature portfolio

Corresponding author(s): Daniel Croymans

Last updated by author(s): 2021/7/5

Reporting Summary

Nature Portfolio wishes to improve the reproducibility of the work that we publish. This form provides structure for consistency and transparency in reporting. For further information on Nature Portfolio policies, see our Editorial Policies and the Editorial Policy Checklist.

Statistics

For all statistical analyses, confirm that the following items are present in the figure legend, table legend, main text, or Methods section.

n/a	Confirmed	
☐	☒	The exact sample size (*n*) for each experimental group/condition, given as a discrete number and unit of measurement
☐	☒	A statement on whether measurements were taken from distinct samples or whether the same sample was measured repeatedly
☐	☒	The statistical test(s) used AND whether they are one- or two-sided<br>*Only common tests should be described solely by name; describe more complex techniques in the Methods section.*
☐	☒	A description of all covariates tested
☐	☒	A description of any assumptions or corrections, such as tests of normality and adjustment for multiple comparisons
☐	☒	A full description of the statistical parameters including central tendency (e.g. means) or other basic estimates (e.g. regression coefficient) AND variation (e.g. standard deviation) or associated estimates of uncertainty (e.g. confidence intervals)
☐	☒	For null hypothesis testing, the test statistic (e.g. *F*, *t*, *r*) with confidence intervals, effect sizes, degrees of freedom and *P* value noted<br>*Give P values as exact values whenever suitable.*
☒	☐	For Bayesian analysis, information on the choice of priors and Markov chain Monte Carlo settings
☒	☐	For hierarchical and complex designs, identification of the appropriate level for tests and full reporting of outcomes
☐	☒	Estimates of effect sizes (e.g. Cohen's *d*, Pearson's *r*), indicating how they were calculated

Our web collection on statistics for biologists contains articles on many of the points above.

Software and code

Policy information about availability of computer code

Data collection	Data about vaccination behaviors and characteristics of participants in randomized controlled trials were collected from UCLA Health records. Data about online participants' responses to text messages and perceptions of COVID-19 and the vaccines were collected via Qualtrics.
Data analysis	Data analysis was conducted in Stata 14 and R 4.1.0.

For manuscripts utilizing custom algorithms or software that are central to the research but not yet described in published literature, software must be made available to editors and reviewers. We strongly encourage code deposition in a community repository (e.g. GitHub). See the Nature Portfolio guidelines for submitting code & software for further information.

Data

Policy information about availability of data

All manuscripts must include a data availability statement. This statement should provide the following information, where applicable:

- Accession codes, unique identifiers, or web links for publicly available datasets
- A description of any restrictions on data availability
- For clinical datasets or third party data, please ensure that the statement adheres to our policy

The two RCTs were pre-registered at clinicaltrials.gov (First RCT: https://clinicaltrials.gov/ct2/show/NCT04800965; Second RCT: https://clinicaltrials.gov/ct2/show/NCT04801524). The three online experiments were pre-registered at aspredicted.org (Online Experiment 1: https://aspredicted.org/blind.php?x=u2ng5c, Online Experiment 2: https://aspredicted.org/blind.php?x=ae3ci5, and Online Experiment 3: https://aspredicted.org/blind.php?x=7wf9er and https://aspredicted.org/blind.php?x=u82hy5). The data analyzed in this paper about randomized controlled trials was provided by UCLA Health and contains protected health information. To protect patient privacy, we cannot publicly post individual-level data. Qualified researchers with a valuable research question and relevant approvals including ethical approval can request access to the de-identified data about these trials from the corresponding author. A formal contract will be signed and an independent

data protection agency should oversee the sharing process to ensure the safety of the data. Data about our online experiments and vaccination intention survey are available at: https://osf.io/qn8hr/?view_only=cf7b2bc590054aee8c4a2bae99ef20c5.

Field-specific reporting

Please select the one below that is the best fit for your research. If you are not sure, read the appropriate sections before making your selection.

☐ Life sciences ☒ Behavioural & social sciences ☐ Ecological, evolutionary & environmental sciences

For a reference copy of the document with all sections, see nature.com/documents/nr-reporting-summary-flat.pdf

Behavioural & social sciences study design

All studies must disclose on these points even when the disclosure is negative.

Study description

Study designs are outlined in the Methods section. In two randomized controlled trials (RCT), we varied whether patients received a text reminder as well as the type of reminder they got, and assessed whether they subsequently scheduled an appointment for the COVID-19 vaccine and eventually obtained the vaccine. In three online experiments, we presented participants with one of four text reminders used in our first RCT, and assessed their interest in getting the COVID-19 vaccine and their perceived persuasiveness of the reminder. These data are all quantitative experimental.

Research sample

The research sample for the RCTs consist of UCLA Health patients who were eligible for the COVID-19 vaccine in January and February 2021, were 18 years old or older, and had not obtained the first dose of COVID-19 vaccine anywhere (as far as UCLA Health could tell) or made a first-dose vaccine appointment at UCLA Health at the time of enrollment in our RCTs. The analysis sample included in the first RCT has 93.354 patients who were enrolled by February 23, 2021 and fit our pre-registered exclusion criteria (43.3% male, 53.5% White (excluding Hispanic or Latino), average age=72.8, s.d.=10.3). The analysis sample for the second RCT includes 67,092 patients who were enrolled by February 23, 2021 and fit our pre-registered exclusion criteria (43.5% male, 52.6% White (excluding Hispanic or Latino), average age=73.7, s.d.=10.0). We chose UCLA patients as our study sample because UCLA Health is one of the largest healthcare systems in California (which allows us to assess a large patient population) and was supportive of evaluating the effectiveness of reminders in promoting COVID-19 appointment scheduling and uptake. Our RCT sample is not representative. See Extended Data Table 7 for a comparison of demographics and vaccination rates between our RCT sample, UCLA Health patients in general, Los Angeles County residents, and California residents.

The research sample for the online experiments consists of US participants from Amazon Mechanical Turk (MTurk) and Prolific Academic (Prolific) who (1) were 18 years old or older, (2) had not taken the COVID-19 vaccine or scheduled an appointment for the COVID-19 vaccine at the time of our studies, and (3) passed a Captcha and an attention check question at the beginning of a given study. Across the two online experiments conducted in February 2021, our final sample after data exclusion (described below) consists of 2,003 participants (51.0% female, 71.8% White (excluding Hispanic or Latino), 51.8% Democrats, average age=37.9, s.d.=13.4). Our online experiment conducted in April 2021 consists of 1,178 participants (53.4% female, 71.6% White (excluding Hispanic or Latino), 40.8% Democrats, average age=36.7, s.d.=12.0) after data exclusion (described below). These samples are not representative and are "convenience samples" from online survey platforms that social scientists commonly use.

Sampling strategy

For the RCTs, we enrolled all UCLA Health patients who met the eligibility criteria by the end of February 23, 2021 (a pre-registered date). The exact sample size could not be determined before RCTs started since we did not know in advance how many patients would fit the eligibility criteria by our pre-registered stopping date. Based on conversations with UCLA Health, we did expect to have at least 30,000 patients enrolled in the first RCT by the end of February 23, 2021, so we knew we had at least an 80% power to detect a 2-percentage-point difference between the Holdout arm and the Follow-Through Text Reminder arm, assuming that the Holdout arm would have a 50% baseline (two-sided proportion test, alpha = 0.05). In the end, our analysis of the first RCT includes 93,354 patients, providing an 80% power to detect a 1.44-percentage-point difference between the Holdout arm and the Text Reminder arm, assuming that the Holdout arm would have a 50% baseline. We were more uncertain about sample size for the second RCT (since we did not know how many people would still choose not to get vaccinated after already receiving a text reminder). Thus, we pre-registered analysis plans contingent on the number of patients enrolled in the second RCT by the end of February 23, 2021. In the end, our analysis of the second RCT includes 67,092 patients, providing an 80% power to detect a 1.54-percentage-point difference between the Holdout arm and the Follow-Through Text Reminder arm, assuming that the Holdout arm would have a 50% baseline. In both RCTs, all patients who fit our pre-registered inclusion/exclusion criteria were enrolled, and there was no sampling from a larger eligible pool.

For the online experiments, we aimed to have at least 800 participants in each experiment, in order to have an 80% power to detect a main effect of video or ownership framing that is as large as Cohen's d of 0.2 (two-sided, alpha = 0.05). The sampling procedure was convenience based: Participants opted into our studies; and once our target sample size was hit for a given study, the study was closed.

Data collection

For the RCTs, data was collected from UCLA Health; no researchers were present for data collection, as enrollment and text message delivery were implemented by UCLA Health and an outside vendor who were blind to the hypotheses. For online experiments, data was collected using online survey software Qualtrics, which completed the randomization into separate experimental conditions; researchers were blind to experimental condition at the data collection stage.

Timing

Participants for the first RCT were enrolled from February 1, 2021 to February 23, 2021. Participants for the second RCT were enrolled from February 9, 2021 to February 23, 2021. Data on vaccination records and participant characteristics were extracted on May 25, 2021. For online experiments, data were collected from 2/21/2021 to 2/23/2021 for the first experiment, from 2/24/2021 to 2/25/2021 for the second experiment, and from 4/23/2021 to 4/29/2021 for the third experiment. More details about each sample can be found in Methods and the Supplementary information.

Data exclusions	For the first RCT, among 132,337 patients enrolled from February 1, 2021 to February 23, 2021, we excluded 33,533 patients who obtained the first dose somewhere before the first reminder date, 5,392 patients who made the first-dose appointment at UCLA Health before 3pm PST, and 58 patients who were below 18 years old. For the second RCT, among 102,675 patients enrolled from February 9, 2021 to February 23, 2021, we excluded 35,127 patients who obtained the first dose somewhere before the second reminder date, 408 who made the first-dose appointment at UCLA Health before 3pm PST, and 48 who were below 18 years old. These exclusions were all pre-registered and implemented using the latest records extracted on May 25, 2021.<br><br>The first online experiment excluded participants who reported having technical problems with the video, or did not complete our pre-registered dependent variables. The second experiment had the same exclusion criteria but additionally excluded Prolific participants who had taken a similar study on Amazon Mechanical Turk. The third experiment had the same exclusion criteria as the second experiment. All of these criteria were pre-registered, except that for the first experiment, we did not pre-register to exclude participants who reported having technical problems with the video. However, since we combined the first and second experiments (both conducted in February concurrently to our RCTs) in our main analysis, we adopted this exclusion criterion (which was pre-registered for the second experiment) consistently for both experiments.
Non-participation	For the RCTs, all participants who fit our eligibility criteria were automatically enrolled, and nobody dropped out. For online experiments, no participants requested to withdraw their responses.
Randomization	Participants in the first RCT were randomly assigned at a 4:1 ratio to either the Follow-Through Reminder arm or the Holdout arm. Patients in the Follow-Through Reminder arm were randomly assigned with an equal probability to one of four subarms: basic reminder, basic reminder with video, ownership reminder, and ownership reminder with video. Participants in the second RCT were randomly assigned at a 6:1 ratio to either the Follow-Through Reminder arm or the Holdout arm. Patients in the Follow-Through Reminder arm were randomly assigned with an equal probability to one of six subarms: basic self, basic prosocial, early access self, early access prosocial, fresh start self, and fresh start prosocial.<br><br>Participants in the online experiments were randomly assigned with an equal probability to read one of the four text messages from our first RCT. In the third experiment, participants were also randomly assigned with an equal probability to answer one of two types of vaccination intention measures.

Reporting for specific materials, systems and methods

We require information from authors about some types of materials, experimental systems and methods used in many studies. Here, indicate whether each material, system or method listed is relevant to your study. If you are not sure if a list item applies to your research, read the appropriate section before selecting a response.

Materials & experimental systems

n/a	Involved in the study	
☒	☐	Antibodies
☒	☐	Eukaryotic cell lines
☒	☐	Palaeontology and archaeology
☒	☐	Animals and other organisms
☐	☒	Human research participants
☐	☒	Clinical data
☒	☐	Dual use research of concern

Methods

n/a	Involved in the study	
☒	☐	ChIP-seq
☒	☐	Flow cytometry
☒	☐	MRI-based neuroimaging

Human research participants

Policy information about studies involving human research participants

Population characteristics	See above, the Methods, and Supplementary Information for more information about participant characteristics
Recruitment	Our RCTs are part of the vaccination outreach effort at UCLA Health. Starting from January 19, 2021, UCLA Health invited patients who were eligible for the COVID-19 vaccine at the time to get vaccinated. UCLA Health sent out invitations to patients in batches. On the first weekday following the initial invitation, eligible patients were automatically enrolled in the first RCT. On the first weekday after the eighth day following the initial invitation, eligible patients were automatically enrolled in the second RCT. Eligibility criteria are described above in Behavioral & Social Sciences study design. Note that since the infrastructure needed to run the RCTs was not ready until February 2021, patients who received the initial invitation during January 19-29, 2021 were enrolled in the first RCT on February 1, 2021 and in the second RCT on February 9, 2021. All other batches of patients were enrolled in the first and/or second RCT based on the aforementioned timeline. Regarding self-selection bias, all patients who fit our enrollment inclusion/exclusion criteria were automatically enrolled and randomized to condition; since patients could not withdraw from the RCTs, no patient was lost to follow up, and treatment could not affect the inclusion/exclusion criteria for our analysis sample, randomization and causal inference are maintained for our analysis sample. See Extended Data Tables 1 and 3 for balance check across conditions.<br><br>For the first online experiment, participants were recruited on MTurk and Prolific from 2/21/2021 to 2/23/2021 in exchange for $0.90 and $1.10, respectively. For the second online experiment, participants were recruited on Prolific from 2/24/2021 to 2/25/2021 in exchange for $1.10. For the third experiment, participants were recruited on MTurk from 4/23/2021 to 4/29/2021 in exchange of $0.90 or $1.00 (We boosted the pay to $1.00 on the third day of our data collection to attract

	more respondents) and Prolific from 4/28/2021 to 4/29/2021 in exchange of $1.10. Regarding self-selection bias, due to informed consent procedures and the use of brief advertisements, people may have chosen to participate based on their knowledge of or interest in our survey topic. This is true for any survey study that involves participant consent. Because participants were randomly assigned to condition, it is unlikely self-selection would result in the effects observed in our online experiment.
Ethics oversight	This research was deemed to comply with all relevant ethical regulations. The Institutional Review Board at the University of California Los Angeles approved the protocols of our randomized controlled trials (reference number 21-000268) and determined that a waiver of informed consent was appropriate. All online experiments and the vaccination intention survey were conducted under approval of the Institutional Review Board at Carnegie Mellon University (reference number IRBSTUDY2015_00000482), and informed consent was obtained from all online study participants as part of the enrollment process.

Note that full information on the approval of the study protocol must also be provided in the manuscript.

Clinical data

Policy information about clinical studies

All manuscripts should comply with the ICMJE guidelines for publication of clinical research and a completed CONSORT checklist must be included with all submissions.

Clinical trial registration	The First RCT: https://clinicaltrials.gov/ct2/show/NCT04800965; The Second RCT: https://clinicaltrials.gov/ct2/show/NCT04801524
Study protocol	Detail about the full trial protocol (the exact content of text messages, access to the video, and access to the survey where the video was embedded) is available in Methods and Supplementary Information.
Data collection	Please see above in the Behavioral & Social Sciences Study Design
Outcomes	Our pre-registered primary outcome measure indicates whether patients scheduled a first-dose appointment at UCLA Health within six days of the first (second) reminder date. We pre-registered this time window because UCLA Health targeted additional outreach efforts to patients who had not scheduled their vaccination appointment six days after the second reminder date and we wanted to use a consistent time window for the two RCTs. Our secondary outcome measure in this paper is whether patients obtained the vaccine at UCLA Health within four weeks of the first (second) reminder date. We chose this window because UCLA Health generally only allowed patients to schedule an appointment for less than four weeks ahead. Consistent with this practice, 96.25% of the first-dose appointments made by patients in the analysis sample of the first RCT occurred within four weeks from the day they were scheduled. In the pre-registrations, we listed additional secondary outcome variables; we explained in Supplementary Information why we did not focus on these in this paper.
